# Impact of combined hormonal contraceptives and metformin on metabolic syndrome in women with hyperandrogenic polycystic ovary syndrome and obesity: The COMET-PCOS randomized clinical trial

**DOI:** 10.1371/journal.pmed.1004662

**Published:** 2025-12-08

**Authors:** Anuja Dokras, Christos Coutifaris, Alan T. Remaley, Nehal N. Mehta, Martin P. Playford, Allen R. Kunselman, Christy C. Stetter, William C. Dodson, Richard S. Legro

**Affiliations:** 1 Department of Obstetrics and Gynecology, University of Pennsylvania, Philadelphia, Pennsylvania, United States of America; 2 National Heart, Lung, and Blood Institute, Bethesda, Maryland, United States of America; 3 George Washington University School of Medicine and Health Sciences, Washington, District of Columbia, United States of America; 4 Department of Public Health Sciences, Pennsylvania State College of Medicine, Hershey, Pennsylvania, United States of America; 5 Department of Obstetrics and Gynecology, M.S. Hershey Medical Center, Hershey, Pennsylvania, United States of America; Dept. Of Obstetrics and Gynecology, NORWAY

## Abstract

**Background:**

The risk-to-benefit ratio of using combined oral contraceptive pills (COCPs) and/or metformin for comprehensive management of polycystic ovary syndrome (PCOS) in women with obesity is unclear. As there is a lack of robust evidence on the impact of these first-line medications on cardiovascular disease (CVD) risk, we compared the effect of COCPs, metformin or both on prevalence of metabolic syndrome (MetS) in participants with hyperandrogenic PCOS and hypothesized that COCPs would increase prevalence of MetS while metformin would decrease prevalence of MetS.

**Methods and findings:**

We conducted a multicenter, double-blind, double-dummy, randomized trial (COMET-PCOS) in participants between ages ≥18 and ≤40 years and body mass index (BMI) ≥25 kg/m^2^ and ≤ 48 kg/m^2^ with hyperandrogenic PCOS (defined by the Rotterdam criteria). Participants were randomized 1:1:1 to 24 weeks of low-dose COCPs (20 μg ethinyl estradiol/0.15 mg desogestrol), metforminXR (2,000 mg), or both (Combined). The primary outcome, assessed by intention-to-treat analysis, was the effect of the different treatment groups on the prevalence of MetS at the end of study. The analytical model included site, race, and the presence or absence of MetS at the screening visit as covariates. The secondary outcomes included changes in each component of MetS (TG, HDL-C, BP, WC, and fasting glucose levels) over the study period. Of the 240 participants randomly assigned, 20 out of 79 in the COCP group, 16 out of 81 in the metformin group, and 17 out of 80 in the combined group dropped out of the study. A total of 169 participants (70.4%) completed the trial between January 2018 and June 2023 (mean age: 29.5 years; mean BMI: 35.6 kg/m^2^; 70% were White and 23% were Black). The overall prevalence of MetS was 31% at baseline and comparable across groups. At the end of the study, the prevalence of MetS was 26.2% (17/65) in the metformin group, 28.6% (17/59) in the Combined group, and 28.8% (17/59) in COCP group with no significant difference in trend of MetS prevalence between groups (adjusted *p* = 0.26). Waist circumference (mean change (MC) −2.23 cm; 95% CI [−3.98, −0.49]; *p* = 0.01), BMI (MC −0.49 kg/m^2^; 95% CI [−0.88, −0.10]; *p* = 0.01), and android fat mass measured by DXA (MC −167 g; 95% CI [−264, −71[; *p* < 0.001) decreased in the COCP group over the study period whilst there was no statistically significant changes in these parameters in the metformin only group when compared to baseline.. In the metformin and Combined groups, the majority of participants (>64%) reported diarrhea, while 24.1% in the COCP group reported uterine bleeding. The main methodologic limitation of the study is the potential lack of power to detect differences in secondary outcomes.

**Conclusions:**

In participants with hyperandrogenic PCOS and overweight/obesity, low-dose COCPs effectively managed PCOS symptoms without increasing prevalence of MetS. Our findings challenge the current practice of using metformin alone or with COCPs for lowering cardiometabolic risk.

**Trial registration:**

ClinicalTrials.Gov Identifier: NCT03229057.

## Author summary

**Why was this study done?:**

- Women with PCOS who are overweight or obese often take birth control pills (COCPs) and/or metformin, but it’s unclear how these medications affect long-term metabolic health.
- Previous research lacked strong evidence on whether these medications reduce or increase cardiovascular risk and no study evaluated metabolic syndrome (MetS), a risk factor for heart disease and diabetes.
- This study aimed to find out whether COCPs, metformin, or both affect MetS in women with PCOS.

**What did the researchers do and find?:**

- 240 women with PCOS and elevated BMI were randomly assigned to take COCPs, metformin, or both for 24 weeks.
- The percentage of women with MetS at the end of the study was not significantly different between the three groups (26.2% metformin, 28.6% combined, 28.8% COCP)
- COCPs use for 24 weeks reduced weight, waist size, and central body fat compared to baseline
- Metformin caused diarrhea in most users and did not decrease the prevalence of MetS even in those who tolerated the maximum dose

**What do these findings mean?:**

- Low-dose COCPs manage PCOS symptoms and did not raise the risk of MetS in this group.
- These results suggest metformin use alone or along with COCPs may not be necessary for reducing metabolic risk in all women with PCOS
- Longer studies are needed to confirm the long-term safety and to better understand side effects and adherence.

## Abstract 3

Anuja Dokras and colleagues compare the effects of oral contraceptive pills, metformin, and a combination of both on metabolic syndrome prevalence in patients with high-risk hyperandrogenic polycystic ovary syndrome.

## Introduction

Polycystic ovary syndrome (PCOS), the most common endocrine disorder in reproductive-age women, is associated with gynecologic, dermatological, psychological, and cardiometabolic comorbidities [1]. Its prevalence ranges from 8%–20% worldwide with approximately 10 million women affected in the U.S. PCOS has a tremendous personal and societal burden and an estimated annual economic cost of $15 billion [2].

For decades, combined oral contraceptive pills (COCPs) have been used as first-line treatment of PCOS to manage irregular menses, hirsutism, and to decrease the risk of endometrial cancer [3]. In the general population, COCP use may increase blood pressure (BP), triglyceride (TG) levels, and possibly weight [4,5]. Use of COCPs may therefore lead to an unacceptable risk-to-benefit ratio for the PCOS population [6], specifically those with hyperandrogenism and obesity, due to an increased prevalence of cardiovascular disease (CVD) risk factors such as hypertension, diabetes, and dyslipidemia in this population [7–9]. In a secondary analysis, one study showed increased risk of metabolic syndrome (MetS) in women with PCOS after using COCPs for 16 weeks [10]. However, there is no data from longer-duration, randomized clinical trials demonstrating this increased composite risk.

Despite the high prevalence of obesity in the PCOS population (50%–70%) and higher prevalence of CVD risk factors in those with hyperandrogenic PCOS, there is also no clear guidance on the optimal management of this PCOS population. Given the role of insulin resistance and compensatory hyperinsulinemia in the development of hyperandrogenism, metformin is commonly prescribed as monotherapy for the treatment of PCOS [11] starting in adolescence or early adulthood for management of insulin resistance, weight, and CVD risk [11,12]. This practice, supported by a recent meta-analysis, however, is based on evidence from primarily low-quality studies showing modest and inconsistent numeric changes of unclear clinical significance in CVD biomarkers [13]. The inclusion of both normoandrogenic and hyperandrogenic participants across all BMI categories in prior studies may have contributed to these mixed observations as individuals with hyperandrogenic PCOS are at increased CVD risk [9,14]. Lack of evidence-based recommendations for the optimal target population or length of use has resulted in many young patients being prescribed metformin for prolonged periods [3]. On the other hand, a recent Cochrane review reported that metformin is less effective than COCPs in regulating menses, improving hirsutism, or decreasing testosterone in PCOS, and its use is associated with significant gastrointestinal (GI) side effects [15]. While it is possible that metformin combined with COCP use may be a beneficial approach as the two medications target distinct mechanisms [3,11], rigorous studies examining the combined impact of these medications on CVD risk especially MetS, in this high-risk population are lacking.

These clinical conundrums have resulted in markedly different prescribing patterns: gynecologists prefer COCPs, endocrinologists prefer metformin, and some patients are prescribed both [16,17]. It is not surprising that patients worldwide express significant frustrations as they desire evidence-based guidance for comprehensive management of PCOS symptoms and associated co-morbidities [18]. To our knowledge, no study has examined the impact of COCPs and metformin on MetS, a condition that signifies the presence of underlying insulin resistance, a common precursor for type 2 diabetes and CVD [19]. Therefore, we conducted a randomized trial to compare the impact of COCPs, metformin, and combination of both on the primary outcome of MetS (COMET-PCOS study) in overweight/obese, hyperandrogenic PCOS individuals and performed in-depth evaluation of each component of MetS as secondary outcomes.

## Methods

### Study design and participants

The COMET-PCOS trial was a multicenter, double-blind, double-dummy, parallel, randomized, clinical trial conducted at the University of Pennsylvania [Penn] and Penn State Health M.S. Hershey Medical Center [PSU], USA between January 15, 2018, and June 28, 2023. The study protocol (S1 Text) was approved by the Penn Institutional Review Board (IRB) which also served as the central IRB (IRB approval number 826447). This study was conducted in accordance with the Declaration of Helsinki and international ethical guidelines. All participants provided written informed consent. An independent data safety and monitoring committee monitored the scientific integrity and the safety of participants. The trial followed the Consolidated Standards of Reporting Trials (CONSORT) guidelines (S1 CONSORT Checklist). There was no direct patient or public involvement in the design of this trial.

Eligible participants were between ages ≥18 and ≤40 years and body mass index (BMI) ≥25 kg/m^2^ and ≤48 kg/m^2^, with PCOS defined by the modified Rotterdam criteria [3]. As individuals with hyperandrogenic PCOS are associated with increased metabolic risk [9,14], only participants with confirmed diagnosis based on clinical (Ferriman Galwey sore) or biochemical hyperandrogenism (elevated total testosterone) and a history of oligomenorrhea (8 or fewer menses per year) or polycystic ovaries on ultrasound were included. Key exclusion criteria were type 1 and 2 diabetes, poorly controlled hypertension, current pregnancy, untreated thyroid disease or hyperprolactinemia, liver or renal disease, use of weight loss medications, or absolute contraindications to the use of COCPs or metformin (full eligibility criteria S1 Table). All participants were deemed to be in good general health by the investigators and willing to avoid pregnancy for the duration of the study. Participants were recruited from the gynecology clinics within both health systems and through social media advertisements. Race and ethnicity were self-reported.

### Randomization and masking

Participants were randomized in a 1:1:1 ratio to continuous COCP (20 μg ethinyl estradiol/0.15 mg desogestrol) and placebo, extended-release metformin (XR) and placebo, or COCP and metformin (combined) daily for 24 weeks. The randomization scheme used variable-size permuted blocks of 3 and 6 and was stratified by site (Penn/PSU), race (Black/non-Black), and the presence/absence of MetS [20] at the screening visit. The random allocation sequence generated by the biostatistician using SAS software, version 9.4 (SAS Institute, Cary, NC) was unknown to the investigators, participants, and study coordinators who enrolled the participants. Placebo pills identical in appearance were administered to participants randomized to OCP or metformin-only groups in order to maintain study blinding.

### Procedures

Once eligibility was confirmed, participants were consented by study coordinators and randomized to one of the 3 groups they received study medications. All medications were self-administered, and participants were asked to take the low-dose COCPs with a less androgenic third-generation progestin (deforester 0.15 mg) once daily for 24 weeks. Extended-release metformin was used and initiated in a step-up fashion starting at 500 mg/day for 5 days and gradually increasing to 2,000mg/day. Participants were provided guidance to take the medications with food and if dose escalation resulted in GI side-effects, the investigators could lower the dose by one tablet till symptoms resolved. After randomization, follow-up visits occurred at 4, 8, 16, and 24 weeks. Urine pregnancy tests were checked, adverse events assessed, study logs reviewed for medication adherence, and new medications and study logs were dispensed at each visit. At 2-, 12-, and 20-week adverse events, menstrual history, medication compliance were also reviewed on the phone. All participants received lifestyle counseling at the randomization and 16-week visit based on the Diabetes Prevention Program. The automated self-administered 24-hour dietary recall (ASA-24) food recall tool was used to assess dietary intake at 12 weeks and end of study. All participants were asked to use barrier contraception during this study and provided with condoms. The protocol deviation report (S2 Text) includes a separate section for remote visits conducted due to the COVID-19 pandemic and approved by the IRB. There were no protocol violations.

### Outcomes

The primary outcome was trend in the prevalence of MetS in the three groups at the end of study period. MetS was defined as presence of ≥3 of the following 5 criteria: TG ≥150 mg/dl, high-density lipoprotein-cholesterol (HDL-C) <50 mg/dl, BP ≥130/ ≥ 85 mmHg or use of anti-hypertensive medications, waist circumference (WC) ≥88 cm (≥80 cm for Asian participants), and fasting glucose ≥100 mg/dl [20]. Secondary endpoints were change in MetS and its components (TG, HDL-C, BP, WC, and fasting glucose levels) from baseline to end of study. Additional secondary outcomes included: change in features of PCOS (hyperandrogenism), body fat distribution, serum lipoproteins, insulin sensitivity, 75 g oral glucose tolerance test, PCOS-specific quality of life measures (PCOSQ) between randomization and end of study visits (S2 Table). Of note, menstrual frequency was added as a post hoc outcome as prior studies have suggested that metformin use may increase menstrual frequency. However, COCPs were prescribed continuously thereby not permitting a comparison between groups. Differences in dietary intake were assessed at 12 and 24 weeks using ASA-24. Safety assessments included adverse events, vital signs, and clinical and laboratory data. Exploratory outcomes related to depression and cognitive testing will be presented elsewhere.

### Statistical analysis

For the primary outcome, a randomized sample size of 240, including a 15% drop-out rate [10], provided 80% statistical power to detect a linear trend in the prevalence of MetS over the 3 groups at the end of the trial, using a two-sided test for linear trend with a significance level of 0.05. Based on prior studies, we assumed a 30% baseline prevalence of MetS [21] with a decrease to 26% in the metformin group, and an increase to 40% in the combined group and 50% in the COCP group at the end of intervention [10].

The analyses invoked the intent-to-treat paradigm for all outcomes. All analytical models included the 3 randomization stratification factors as covariates, except for models assessing the change over time for MetS or its components which included recruitment site and race as covariates. For analysis of the primary outcome, binary logistic regression was used and a contrast was constructed from this model to test for linear trend in MetS over the three treatment arms at the end of study defined by the last completed standard visit ≥16 weeks or early termination visit after 12 weeks. Secondary outcomes were analyzed with longitudinal models that included treatment arm, visit number, and their interaction, as well as the stratification factors, as fixed effects. Changes over time in MetS and its components were assessed via binary logistic regression using generalized estimating equations (GEE); the effect sizes were quantified using odds ratios (ORs) and 95% confidence intervals (CIs). For continuous secondary outcomes, linear mixed models were used to assess differences in change in the responses over time between groups. Residual diagnostics from mixed models were examined and, when necessary, log-transformations of the response were used to meet parametric modeling assumptions. Contrasts were constructed to quantify change from baseline as a difference in estimated marginal means with 95% CIs, or in the event of log-transformation was used a ratio of geometric marginal means (RGM). All hypothesis tests were two-sided. As secondary outcomes were considered exploratory, no adjustments for multiple hypothesis testing were performed and point estimates and 95% CIs are provided. No interim analyses were performed. All statistical analyses were based on the statistical analysis plan (S2 Text). We performed a post-hoc sensitivity analysis for our primary outcome based on presence or absence of MetS at baseline. We also conducted a sensitivity analysis of the primary outcome of trend over the 3 treatment groups based on the subset of subjects who reached the full dosage of metformin. The trial data were monitored by an independent data monitoring committee and the trial was registered at ClinicalTrials.Gov Identifier: NCT03229057 on 07/13/2017 URL clinicaltrials.gov

## Results

### Participants

**Fig 1:**
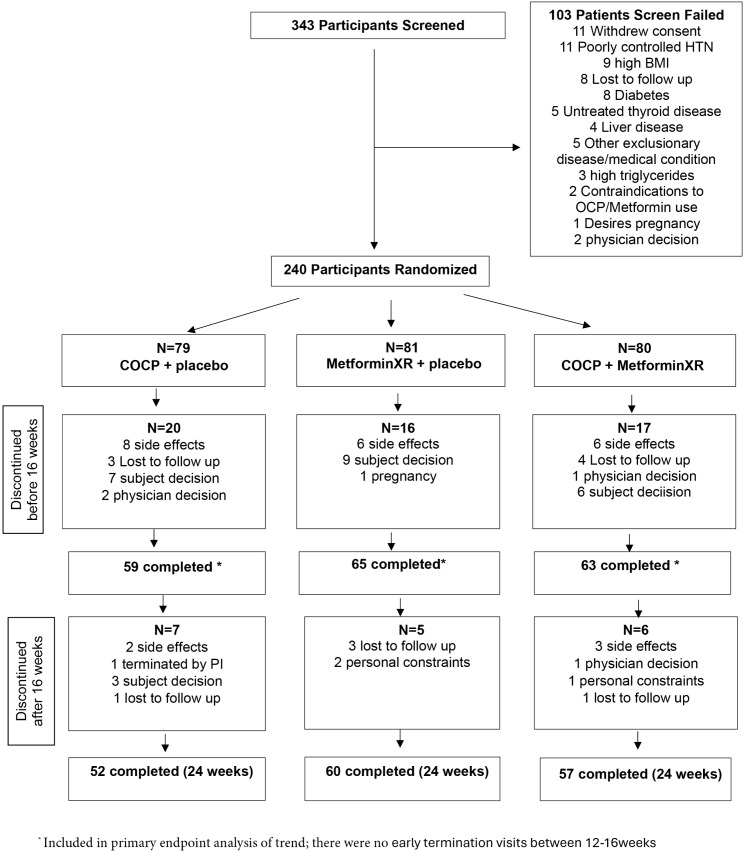
Trial participant flow chart for COMET PCOS trial with participants randomized to continuous oral contraceptive pills (COCP), extended-release metformin (Metformin XR), or both (COCP + metformin XR).

A total of 343 participants were screened, 240 were randomized and 169 participants (70.4%) completed the trial between January 2018 and June 2023, with similar discontinuation rates between groups (Fig 1). The demographic and clinical characteristics at baseline were well balanced across groups and are shown in Tables 1 and S3. The mean age of the participants was 29.5years, mean BMI was 35.6 kg/m^2^, and 70% of the participants were White and 23% were Black. The overall prevalence of MetS at baseline was 31%.

**Table 1:** Baseline demographic and clinical characteristics of participants.

	COCP (*N* = 79)	Metformin (*N* = 81)	Combined (*N* = 80)
**Demographics**			
Age mean (SD) years	29.1 (4.8)	30.1 (5.3)	29.2 (5.4)
Hispanic ethnicity: *N* (%)	11 (13.9%)	17 (21.0%)	14 (17.5%)
Race: *N* (%)			
* White*	53 (67.1%)	59 (72.8%)	57 (71.3%)
* Black*	19 (24.1%)	16 (19.8%)	19 (23.8%)
* Asian*	5 (6.3%)	3 (3.7%)	3 (3.8%)
* Native Hawaiian/Other Pacific Islander*	0 (0.0%)	1 (1.2%)	0 (0.0%)
* More than one race*	2 (2.5%)	2 (2.5%)	1 (1.3%)
Menses’ per year mean (SD)^a^	4.7 (3.2)	5.7 (3.8)	4.8 (3.3)
Nulliparous: *N* (%)	56 (70.9%)	57 (70.4%)	58 (72.5%)
Current smoker: *N* (%)	6 (7.6%)	5 (6.2%)	5 (6.3%)
Hypertension medications: *N* (%)	7 (8.9%)	2 (2.5%)	2 (2.5%)
H/o depression/anxiety: *N* (%)	42 (53.2%)	50 (62.5%)	46 (57.5%)
Depression/anxiety medications: *N* (%)	21 (26.6%)	26 (32.1%)	18 (22.5%)
Prediabetes (based on HbA1c): *N* (%)	27 (34.1%)	27 (33.1%)	27 (33.7%)
Metabolic syndrome (%)	24 (30.4%)	25 (31.3%)	25 (31.2%)
**Biometric parameters**			
Weight mean (SD) kg	96.7 (17.6)	94.8 (16.5)	98.0 (17.3)
BMI mean (SD) kg/m^2^	35.2 (5.6)	35.8 (5.8)	35.9 (5.9)
Waist circumference mean (SD) cm	108.0 (12.8)	107.4 (15.6)	109.0 (16.4)
Systolic BP mean (SD) mmHg	116.8 (9.8)	116.9 (10.1)	117.0 (8.4)
Diastolic BP mean (SD) mmHg	76.8 (7.3)	77.7 (6.7)	76.9 (6.3)
**PCOS diagnosis parameters**			
Total testosterone mean (SD) ng/dL	43.7 (19.6)	42.8 (19.4)	46.6 (23.3)
Free testosterone mean (SD) pg/mL^b^	6.1 (3.4)	5.6 (3.0)	6.2 (3.3)
SHBG median (IQR) nmol/L	32.3 (22.6, 44.2)	34.0 (22.6, 47.9)	32.6 (23.2, 44.1)
Ferriman-Gallwey Hirsutism Score mean (SD)	15.4 (7.5)	16.0 (7.5)	16.5 (7.7)
AMH median (IQR) ng/mL	5.9 (4.2, 10.4)	5.3 (3.6, 7.6)	6.7 (4.2, 9.7)
Antral Follicle Count median (IQR)^c^	21.0 (16.5, 27.0)	20.0 (14.5, 25.0)	23.8 (18.5, 29.0)
Ovarian Volume median (IQR) cm^3^^c^	10.6 (8.5, 14.7)	11.9 (8.5, 14.7)	12.1 (9.3, 17.6)
**Oral Glucose Tolerance test (oGTT)** ^d^			
Fasting Glucose median (IQR) mg/dl	93.0 (87.0, 101.0)	91.0 (86.0, 99.0)	93.0 (83.0, 99.0)
Fasting Insulin median (IQR) uU/ml	16.8 (12.5, 26.3)	15.1 (10.0, 22.8)	15.6 (11.3, 26.4)
2-hour Glucose median (IQR) mg/dl	113.0 (92.0, 130.0)	110.0 (93.0, 133.0)	105.0 (84.0, 127.0)
2-hour Insulin median (IQR) uU/ml	77.6 (53.7, 172.3)	75.4 (38.2, 150.7)	91.3 (42.8, 155.1)
AUC Glucose median (IQR) mg/dl * hour	263.1 (219.3, 294.6)	262.6 (224.5, 286.0)	245.4 (220.5, 290.0)
AUC Insulin median (IQR) uU/ml * hour	226.6 (134.1, 362.7)	185.7 (131.0, 284.4)	214.9 (141.2, 312.9)
Matsuda’s Insulin Sensitivity Index (ISI) median (IQR)	2.2 (1.3, 3.8)	2.5 (1.7, 4.0)	2.5 (1.5, 4.2)
HOMA-IR median (IQR)	2.2 (1.6, 3.3)	1.9 (1.3, 3.0)	2.1 (1.5, 3.4)
**DXA parameters** ^e^			
Total Area mean (SD) cm^2^	2087.2 (206.6)	2033.6 (163.6)	2070.8 (158.6)
Fat mass mean (SD) kg	42.8 (11.1)	42.0 (10.3)	43.5 (10.8)
Lean mass mean (SD) kg	52.4 (8.2)	51.2 (7.1)	52.2 (7.2)
Total Mass (Fat+Lean) mean (SD) kg	95.2 (17.4)	93.3 (16.2)	95.7 (16.4)
% Fat mean (SD)	44.5 (5.2)	44.6 (4.5)	45.0 (4.6)
Android Fat mean (SD) g	3703.0 (1269.5)	3683.1 (1280.1)	3774.2 (1372.0)
Gynoid Fat mean (SD) g	7085.2 (1859.7)	6888.7 (1662.6)	7260.5 (1718.3)
Android/Gynoid Ratio mean (SD)	0.53 (0.13)	0.53 (0.13)	0.52 (0.13)

To convert the values for cholesterol, high-density lipoprotein (HDL) cholesterol and low-density lipoprotein (LDL) cholesterol to millimoles per liter, multiply by 0.02586. To convert the values for triglycerides to millimoles per liter, multiply by 0.01129. To convert the values for glucose to millimoles per liter, multiply by 0.05551. To convert the values for testosterone to nanomoles per liter, multiply by 0.034, To convert the values for insulin to picomoles per liter, multiply by 6.95

^a^Menses data were missing in 4 participants in the COCP group, 2 participants in the metformin group and 1 participant in the combined group.

^b^Free testosterone values were missing for 1 participant in the COCP group, 2 participants in the metformin group and 3 participants in the combined group.

^c^Average of both ovaries. Ovarian volume calculation was missing for 1 participant in the COCP group, while antral follicle count data were missing for 4 participants in the COCP group, 2 in the metformin group and 2 in the combined group.

^d^The 2 h oGTT glucose and insulin values were missing in 2 participants in the metformin group and 1 participant in the combined group. Seven participants in the COCP group, 3 participants in the metformin group and 6 participants in the combined group were missing calculation of AUC from the oGTT. Six participants in the COCP group, 3 in the metformin group, and 5 in the combined group were missing Matsuda’s ISI.

^e^DXA parameters were missing in 2 participants in the metformin group and 2 participants in the combined group

**Abbreviations:** DXA, Dual-energy X-ray absorptiometry; HOMA-IR, homeostatic model assessment of insulin resistance; AUC, area under the curve; AMH, anti mullerian hormone; SHBG, sex hormone binding globulin; BP, blood pressure; BMI, body mass index.

### Primary outcome

The unadjusted prevalence of MetS at the end of the study was 26.2% in the metformin group (17/65), 28.6% in the combined group (18/63), and 28.8% in the COCP group (17/59); the test for a linear trend over the 3 groups, adjusted for the 3 randomization factors, was not significant (OR for linear trend = 1.32 95% CI [0.82, 2.15], *p* = 0.26).

### Secondary outcomes

#### Change in MetS compared to baseline.

**Fig 2:**
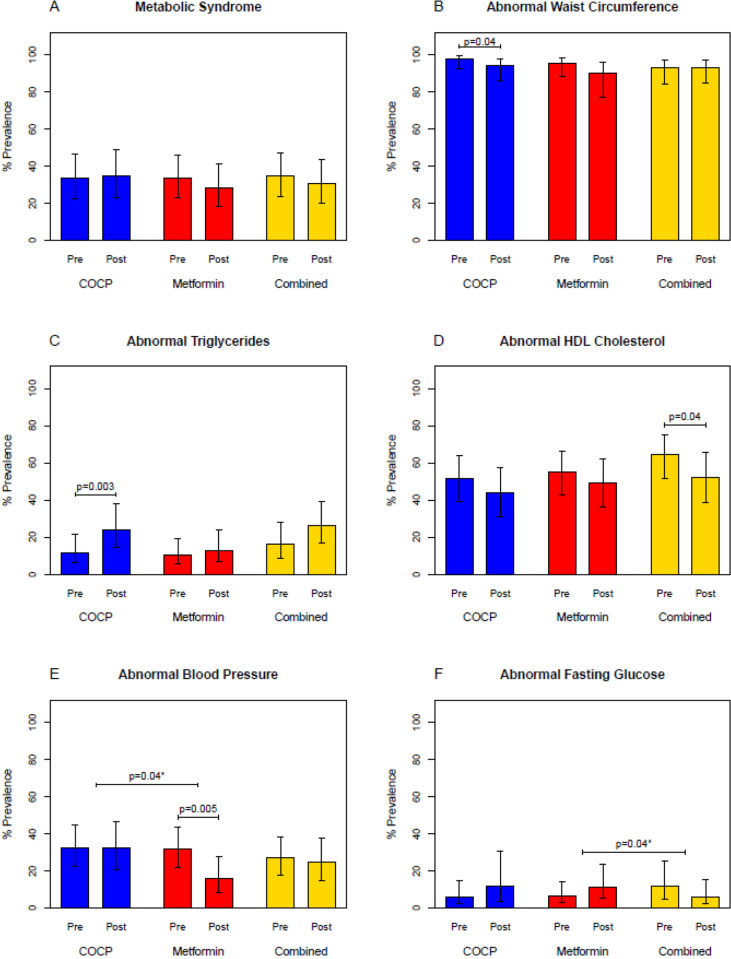
Metabolic syndrome and individual components at baseline and end of study. Panel **A** shows the change in prevalence of MetS between baseline values (pre) and at the end of study (post) in the combined oral contraceptive pills (COCP) group, metformin group, and combined group. MetS was defined as presence of at least 3 of the following 5 criteria: TG ≥150 mg/dl, HDL-C <50 mg/dl, BP ≥130/ ≥ 85 mmHg or use of anti-hypertensive medications, waist circumference ≥88 cm (≥80 cm for Asian participants), and fasting glucose ≥100 mg/dl. Panels **B**–**F** show the change in prevalence of abnormal criterion for individual components of MetS. *P*-values for significant differences within and between groups are displayed; the significant differences in the change (pre to post) between groups are denoted with an asterisk. Error bars represent the 95% confidence interval (CI) for the proportion. Estimates for these proportions, 95% CIs, and *p*-values for comparisons, were obtained via binary logistic regression to account for repeated measurements per subject and using GEE adjusting for race (Black/non-Black) and recruitment site (Penn/PSU).

There were no significant changes in the prevalence of MetS between baseline and study completion within any group and no significant between-group differences (Table 2 [unadjusted results shown in S4 Table], Fig 2). When examining between-group differences for individual components, only change in BP criterion was significant between COCP and metformin groups (OR 1.00 (95% CI [0.56, 1.78] versus OR 0.41 (95% CI [0.22, 0.77], *p* = 0.04) and change in fasting glucose criterion between metformin and the combined group (OR 1.83 (95% CI [0.93, 3.58] versus OR 0.49 (95% CI [0.16, 1.47], *p* = 0.04).

**Table 2:** Change in prevalence of metabolic syndrome (MetS) and its components between baseline (pre) and end of study visits (post).

	COCP Post vs. Pre		Metformin Post vs. Pre		Combined Post vs. Pre		^a^COCP vs. Metformin	^a^COCP vs. Combined	Metformin vs. Combined^a^
	**OR (95% CI)**	***P*-value**	**OR (95% CI)**	***P*-value**	**OR (95% CI)**	***P*-value**	***P*-value**	***P*-value**	***P*-value**
**Metabolic syndrome**	1.05 (0.67, 1.66)	0.82	0.78 (0.44, 1.38)	0.39	0.83 (0.47, 1.49)	0.53	0.41	0.53	0.87
**MetS abnormal criterion** **definition**									
Waist circumference ≥88 cm (≥80 cm for Asian participants)	0.38 (0.15, 0.97)	0.04	0.45 (0.20, 1.02)	0.06	1.00 (0.42, 2.41)	0.99	0.80	0.14	0.19
Triglyceride ≥150 mg/dl	2.37 (1.34, 4.18)	0.003	1.26 (0.69, 2.32)	0.45	1.85 (1.00, 3.44)	0.05	0.14	0.56	0.39
HDL <50 mg/dl	0.74 (0.48, 1.13)	0.16	0.79 (0.48, 1.31)	0.36	0.61 (0.39, 0.97)	0.04	0.82	0.57	0.46
Blood pressure ≥130/85 mmHg or use of anti-hypertensive medications	1.00 (0.56, 1.78)	1.00	0.41 (0.22, 0.77)	0.005	0.89 (0.48, 1.66)	0.72	0.04	0.79	0.08
Fasting Glucose ≥100 mg/dl^b^	2.02 (0.84, 4.88)	0.12	1.83 (0.93, 3.58)	0.08	0.49 (0.16, 1.47)	0.21	0.86	0.05	0.04

Odds ratios were obtained via binary logistic regression using GEE adjusting for race and site and calculated as [odds at end of study]/[odds at baseline].

^a^comparison of the ORs between groups.

^b^ype 1 and 2 diabetes was an exclusion criterion

**Abbreviation:** HDL, high density cholesterol.

#### Change in PCOS symptoms.

Mean changes in body weight-related outcomes namely WC (−2.23 cm (95% CI [−3.98, −0.49], *p* = 0.01), BMI (−0.49 kg/m^2^ (95% CI {−0.88, −0.10}, *p* = 0.01) (Table 3 [unadjusted results shown in S5 Table)) and PCOSQ-weight domain scores (0.72 points (95% CI [0.37, 1.07], *p* < 0.001) (S5 Table) were seen in the COCP group along with a decrease in the MetS WC criterion (OR 0.38 (95% CI [0.15, 0.97], *p* = 0.04) (Table 2) with no statistically significant change from baseline within the metformin group. Correspondingly, dual-energy X-ray absorptiometry (DXA) measurements showed a decrease in total mass (mean change −1.74 kg (95% CI {−2.79, −0.70], *p* = 0.001) and android fat (mean change −167g (95% CI [−264, −71], *p* < 0.001) in the COCP group with no change within the metformin group (Table 3). Of note, there were no differences between groups in the Healthy Eating Index (HEI) scores derived from the ASA-24 at 12 and 24 weeks (S6 Table). For outcomes related to hyperandrogenism, a decrease in total and free testosterone levels was observed within the COCP and combined groups compared to the metformin-only group (Table 3). There was also a corresponding increase in sex hormone binding globulin (SHBG) levels and improvement in the PCOSQ hair domain scores in the COCP groups (S7 Table).

**Table 3:** Change in Secondary Outcomes from Baseline to End of Study Visits within each group and comparison between OCP, metformin, and combined groups.

	COCP (*n* = 79)		Metformin (*n* = 81)		Combined (*n*-80)		COCP vs. Metformin	COCP vs. Combined	Metformin vs. Combined
	**Change from Baseline** **(95% CI)** ^a^	***P*-value**	**Change from Baseline** **(95% CI)** ^a^	***P*-value**	**Change from Baseline** **(95% CI)** ^a^	***P*-value**	***P*-value**	***P*-value**	***P*-value**
**Biometric parameters**									
Weight (kg)	−1.40 (−3.02, 0.23)	0.09	−0.24 (−1.77, 1.29)	0.75	−1.31 (−2.86, 0.25)	0.10	0.31	0.94	0.34
BMI (kg/m^2^)	−0.49 (−0.88, −0.10)	0.01	−0.09 (−0.46, 0.28)	0.65	−0.47 (−0.85, −0.09)	0.01	0.14	0.95	0.15
Waist circumference (cm)	−2.23 (−3.98, −0.49)	0.01	−1.66 (−3.32, 0.00)	0.05	−2.44 (−4.12, −0.75)	0.005	0.64	0.87	0.52
Systolic BP (mmHg)	2.68 (0.53, 4.83)	0.01	0.66 (−1.40, 2.71)	0.53	3.21 (1.13, 5.30)	0.003	0.18	0.73	0.09
Diastolic BP (mmHg)	1.91 (0.44, 3.39)	0.01	0.18 (−1.23, 1.59)	0.80	1.51 (0.08, 2.93)	0.04	0.10	0.70	0.19
**PCOS diagnosis parameters**									
Total Testosterone^b^	0.63 (0.55, 0.71)	<0.001	0.88 (0.77, 0.99)	0.03	0.68 (0.60, 0.77)	<0.001	<0.001	0.35	0.006
Free Testosterone^b^	0.29 (0.23, 0.35)	<0.001	0.81 (0.66, 0.99)	0.04	0.36 (0.30, 0.44)	<0.001	<0.001	0.10	<0.001
SHBG^b^	3.37 (2.91, 3.90)	<0.001	1.07 (0.93, 1.23)	0.34	2.70 (2.34, 3.12)	<0.001	<0.001	0.04	<0.001
Ferriman-Gallwey Score	−1.72 (−2.77, −0.66)	0.002	−1.61 (−2.61, −0.61)	0.002	−2.82 (−3.83, −1.81)	<0.001	0.89	0.14	0.09
AMH^b^	0.69 (0.63, 0.75)	<0.001	1.01 (0.93, 1.10)	0.77	0.76 (0.69, 0.83)	<0.001	<0.001	0.14	<0.001
Antral Follicle Count^b^	0.77 (0.68, 0.88)	<0.001	1.04 (0.92, 1.18)	0.49	0.75 (0.67, 0.85)	<0.001	0.001	0.75	<0.001
Total Ovarian Volume^b^	0.61 (0.53, 0.71)	<0.001	1.00 (0.87, 1.16)	0.96	0.70 (0.60, 0.80)	<0.001	<0.001	0.21	<0.001
**Oral Glucose Tolerance Test (oGTT)**									
Fasting Glucose^b^	0.97 (0.95, 1.00)	0.04	0.99 (0.97, 1.02)	0.55	0.97 (0.95, 1.00)	0.04	0.27	0.91	0.31
2-hour Glucose^b^	1.13 (1.05, 1.22)	0.001	1.00 (0.93, 1.07)	0.92	1.15 (1.07, 1.23)	<0.001	0.01	0.78	0.006
AUC Glucose^b^	1.08 (1.02, 1.14)	0.004	1.00 (0.95, 1.05)	0.98	1.04 (0.99, 1.09)	0.13	0.04	0.28	0.30
Fasting Insulin^b^	1.04 (0.89, 1.22)	0.64	1.13 (0.96, 1.31)	0.13	1.03 (0.88, 1.20)	0.75	0.47	0.91	0.40
2-hour Insulin^b^	1.09 (0.90, 1.33)	0.37	0.85 (0.70, 1.02)	0.08	1.17 (0.97, 1.42)	0.09	0.07	0.61	0.02
AUC Insulin^b^	1.07 (0.96, 1.21)	0.23	0.93 (0.83, 1.04)	0.18	1.03 (0.92, 1.15)	0.62	0.07	0.59	0.19
Matsuda’s Insulin Sensitivity Index^b^	0.94 (0.83, 1.07)	0.33	1.01 (0.90, 1.14)	0.86	0.93 (0.83, 1.05)	0.23	0.40	0.92	0.33
HOMA-IR^b^	1.04 (0.89, 1.22)	0.61	1.05 (0.90, 1.22)	0.52	1.01 (0.87, 1.18)	0.85	0.94	0.81	0.75
**DXA parameters**									
Fat mass (kg)	−0.83 (−1.65, −0.01)	0.05	−0.48 (−1.24, 0.28)	0.21	0.06 (−0.70, 0.82)	0.88	0.54	0.12	0.32
Lean mass (kg)	−0.93 (−1.44, −0.41)	<0.001	−0.06 (−0.54, 0.42)	0.80	−1.24 (−1.72, −0.76)	<0.001	0.02	0.38	<0.001
Total Mass (kg) (Fat+Lean)	−1.74 (−2.79, −0.70)	0.001	−0.55 (−1.51, 0.42)	0.27	−1.17 (−2.14, −0.20)	0.02	0.10	0.43	0.37
% Fat	0.00 (−0.53, 0.53)	0.99	−0.29 (−0.78, 0.20)	0.24	0.55 (0.06, 1.04)	0.03	0.42	0.14	0.02
Android Fat (g)	−167 (−264, −71)	<0.001	−86 (−176, 4)	0.06	−58 (−148, 32)	0.20	0.22	0.10	0.67
Gynoid Fat (g)	−130 (−270, 9)	0.07	−43 (−171, 86)	0.52	−5 (−134, 124)	0.94	0.36	0.19	0.69
Android/ Gynoid fat ratio	−0.013 (−0.023, −0.003)	0.01	−0.008 (−0.017, 0.001)	0.09	−0.007 (−0.017, 0.002)	0.13	0.46	0.39	0.90

^a^Change based on estimated marginal means adjusted for the randomization stratification factors of site, race, and metabolic syndrome.

^b^Data were log-transformed for analysis and changes reported as ratio of geometric marginal means.

**Abbreviations:** DXA, Dual-energy X-ray absorptiometry; HOMA-IR, homeostatic model assessment of insulin resistance; AUC, area under the curve; AMH, anti mullerian hormone; SHBG, sex hormone binding globulin;

BP, blood pressure; BMI, body mass index.

#### Change in insulin sensitivity and glucose tolerance.

There were no differences in insulin sensitivity (Homeostatic Model Assessment of Insulin Resistance (HOMA-IR) or Matsuda’s index) within the groups (Table 3). Although there was no significant change in prevalence of abnormal glucose criterion within the three groups (Table 2), the 2-hour oGTT increased in the COCP group (RGM 1.13; 95% CI [1.05, 1.22]; p = 0.001) and addition of metformin in the combined group did not mitigate the change (RGM 1.15; 95%CI [1.07, 1.23]; *p* < 0.001).

#### Change in lipoproteins.

Total HDL particles increased significantly compared to baseline within the COCP and combined groups compared to the metformin group (S5 Table). Although more participants in the COCP group met the abnormal TG criterion at the end of the study (OR 2.37 (95%CI [1.34, 4.18], *p* = 0.003) (Table 2 and Fig 2), there were no significant differences in total TG rich lipoproteins (TRLP) measured by NMR between groups (S4 Table, pages 41–43). Total LDL particles and small-LDL particles increased within the COCP and combined groups.

### Post hoc sensitivity analysis

We performed a subgroup analysis for our primary outcome based on presence or absence of MetS at baseline. In the subgroup with MetS at baseline (prevalence 100%), prevalence of MetS at end of study decreased in all groups; 55% metformin,57% combined, and 80% COCP groups, with OR =2.33, 95% CI [1.01, 5.37], *p* = 0.047. For the subgroup without MetS at baseline, the prevalence of MetS increased in all groups at the end of the study; metformin 13%, Combined 14% and COCPs 11% (OR 0.92, 95% CI [0.48–1.7], *p* = 0.79). The transitions between MetS status at baseline and end of study in each group are shown in S7 Table. We also conducted a sensitivity analysis of the primary outcome of trend over the 3 treatment groups based on the subset of subjects who reached the full dosage of metformin. The prevalence of MetS at the end of study was 21.2% in the metformin group (11/52), 27.9% in the combined group (12/43), and 28.3% in the COCP only group (17/59) (OR 1.42, 95% CI [0.83–2.4], *p* = 0.19).

We also examined change in menstrual frequency in the metformin only group. The number of menses over the study period increased compared to number of menses pre-intervention, with a mean difference of 0.42 (95% CI [−0.05, 0.89], *p* = 0.08), whilst use of COCPs offered continuous endometrial suppression in the other two groups. Of note, an improvement in PCOS emotion scores was noted in all groups compared to baseline (S5 Table).

### Safety outcomes

A majority of participants in both metformin groups reported diarrhea (>64%, Table 4) and reported significantly more abdominal pain, cramping, and vomiting compared to the COCP only group. At week 16, only 75% of participants receiving metformin were dispensed the maximum dose of 2,000 mg/day. More participants in the COCP group reported abnormal vaginal bleeding compared to both the metformin groups (Table 4). Across the 3 treatment arms, approximately 8% of participants discontinued the study prior to 16 weeks due to medication-related side effects (Fig 1). Overall, all three groups had similar rates of serious adverse events (S6 Table, page 44).

**Table 4:** Key safety measures including adverse events.

	COCP (*N* = 79)	Metformin (*N* = 81)	Combined (*N* = 80)	*P*-value^a^
**All Adverse Events**				
Any event	71 (89.9%)	76 (93.8%)	78 (97.5%)	0.13
Serious adverse event	2 (2.5%)	3 (3.7%)	3 (3.8%)	1.00
* Hospitalizations*	1 (1.3%)	3 (3.7%)	2 (2.5%)	0.87
* Significant event requiring intervention*	1 (1.3%)	0 (0.0%)	1 (1.3%)	0.55
**Individual Adverse Events (not serious, more than 5% within any treatment arm)**				
Gastrointestinal symptoms:				
* *Diarrhea	27 (34.2%)	52 (64.2%)	52 (65.0%)	<0.001
* *Nausea	29 (36.7%)	34 (42.0%)	37 (46.3%)	0.47
* *Abdominal pain/cramping	10 (12.7%)	27 (33.3%)	20 (25.0%)	0.01
* *Vomiting	8 (10.1%)	20 (24.7%)	18 (22.5%)	0.04
* *Abdominal bloating	10 (12.7%)	9 (11.1%)	9 (11.3%)	0.94
* *Dyspepsia	4 (5.1%)	5 (6.2%)	12 (15.0%)	0.06
* *Decreased appetite	3 (3.8%)	3 (3.7%)	7 (8.8%)	0.29
* *Flatulence	6 (7.6%)	9 (11.1%)	4 (5.0%)	0.36
Uterine Symptoms:				
* *Bleeding/Spotting	19 (24.1%)	2 (2.5%)	7 (8.8%)	<0.001
* *Dysmenorrhea^b^	0 (0%)	3 (4.7%)	5 (6.3%)	0.09
Breast symptoms	7 (8.9%)	2 (2.5%)	5 (6.3%)	0.20
Dizziness	3 (3.8%)	6 (7.4%)	4 (5.0%)	0.68
Headache/migraine	21 (26.6%)	22 (27.2%)	21 (26.3%)	1.00
Mood swings	10 (12.7%)	3 (3.7%)	3 (3.8%)	0.06
Joint/muscle pain	4 (5.1%)	5 (6.2%)	3 (3.8%)	0.88
Upper respiratory infection	16 (20.3%)	14 (17.3%)	8 (10.0%)	0.18
COVID infection/symptoms after exposure	7 (8.9%)	3 (3.7%)	4 (5.0%)	0.35
Fatigue	4 (5.1%)	9 (11.1%)	6 (7.5%)	0.38

^a^Freeman–Halton test.

^b^Severe dysmenorrhea reported by patient.

## Discussion

In this randomized clinical trial, we hypothesized that the use of COCP would increase prevalence of MetS and its components and metformin alone or in combination with COCPs would mitigate this risk in individuals with hyperandrogenic PCOS. However, we observed no significant difference in the prevalence of MetS at the end of treatment with COCPs, metformin, or the combination and no changes in prevalence of MetS within each group when compared to baseline. In addition, COCP use was associated with improvements in patient-prioritized concerns related to PCOS namely, symptoms of hyperandrogenism, weight, and body fat distribution [18]. Collectively, these findings support the use of low-dose COCPs alone as first-line therapy for comprehensive treatment of hyperandrogenic patients who are overweight or obese. Our results further challenge the use of metformin alone or with COCPs in all patients with PCOS and obesity [3], as metformin showed limited additive benefit in our primary and exploratory outcomes related to CVD risk and was associated with a significantly higher prevalence of side effects.

COCPs decrease bioavailable androgens by suppressing pituitary gonadotropin secretion and increasing SHBG. Compared to the 30–35 μg ethinyl estradiol COCP used in most published studies in women with PCOS [13,15], our choice of a lower dose 20 μg ethinyl estradiol COCP aligned with current PCOS guidelines [3] and may explain some of our favorable findings in the COCP alone group. More importantly, despite using a low-dose COCP, we observed significant suppression of androgens and increase in SHBG compared to baseline in our hyperandrogenic study population. As androgen excess predisposes to central obesity [22], it is possible that greater suppression of androgens in the COCP group may have resulted in the decrease in DXA-measured android fat and abnormal waist circumference MetS criterion noted in this group. This favorable outcome may have contributed to the neutral impact of COCPs on our primary outcome of MetS including the fasting glucose criterion. Additionally, we did not observe significant changes in insulin sensitivity indices as shown in some prior studies [23]. Of note, as most prior studies included women with both normoandrogenic and hyperandrogenic PCOS, they may not have observed the benefits attributed to androgen suppression explaining differences in some outcomes when compared to our study [13]. As serum androgens are associated with incident CVD events in postmenopausal women [24], it is plausible that suppression of androgens from an early age in PCOS may have long-term benefits. This hypothesis will, however, need to be explored in longer intervention studies.

To further characterize CVD risk, we performed deep lipid phenotyping. Prior studies show mixed results; COCPs may increase TG and HDL-C levels whereas metformin may have favorable effects on LDL-C [13,25]. In our study, there was an increase in prevalence of the abnormal TG criterion of MetS at the end of the study within the COCP group. On the other hand, our findings demonstrate that COCP use was associated with increased anti-atherogenic HDL-C particle numbers compared to metformin, reflecting the effect of estrogen on hepatic lipase activity [26]. We also did not observe significant change in atherogenic large TRLP within the COCP group. We previously reported an increase in HDL-C efflux capacity after 16-week low-dose COCP use, and those findings correlated with decrease in testosterone levels [27]. In the setting of these beneficial though exploratory findings, the long-term significance of the increase in sLDL-C particles in COCP groups needs to be further explored.

Treating insulin resistance with metformin for CVD risk reduction in PCOS is controversial. Evidence supporting the use of metformin compared to COCP is derived from low-grade, short-duration studies that show a decrease in insulin resistance and serum TG levels [13]. In our study, we did not find a decrease in either prevalence of MetS or an improvement in markers of IR in the metformin only group. Our study length was longer than most in the literature, and our findings may reflect the impact of a significant percentage of participants not tolerating the maximum dose of metformin by 16 weeks. Interestingly, we also did not observe significant changes in the glucose criterion for MetS or oGTT values in the metformin group, likely a reflection of normoglycemia at baseline in the majority of the study participants. We did observe a decrease in the prevalence of the abnormal BP criterion in the metformin group although this was not supported by a change in the mean systolic or diastolic BP values at the end of the study. Furthermore, we did not observe a significant change in BMI, WC criterion, or fat distribution in the metformin-only group, despite the common use of metformin for this indication [11,12]. Prior studies that report modest weight loss with metformin use, typically include intensive lifestyle changes [28]. It should be highlighted that in our study, we used a higher dose of metformin compared to most studies in PCOS [13], and provided real-world nutritional counseling recognizing that most people are unable to engage in prolonged dietary and exercise changes. It was not surprising that similar to prior studies we found that metformin alone was inferior for management of PCOS features, such as hyperandrogenism and menstrual irregularity, compared to both COCP groups [13,15]. The exploratory lipid phenotyping data also did not show overall benefit to using metformin alone. As shown in prior studies, we also observed that the GI side-effects lowered patient compliance and acceptance of higher doses [13,29] further decreasing support for metformin use, alone or combined. Instead, assessment of CVD risk including elucidating a history of gestational diabetes and family history of diabetes, as recommended in the international PCOS guidelines [3], can be used to risk-stratify patients and individualize therapies.

Our study has many strengths. First, recognizing the low prevalence of CVD events in a reproductive age population and based on our prior findings [10], we proposed a pragmatic approach by assessing MetS as our primary outcome. Thereby, we have added new data to the existing literature where most studies have only evaluated changes in numeric values of CVD biomarkers such as glucose, insulin, and lipids. Second, given that PCOS is a heterogeneous condition and the individuals with hyperandrogenic PCOS are at risk for CVD, our study was specifically designed to offer counseling for this highly prevalent group. Third, we designed a double-blind, double-dummy trial that included a metformin-only group, as providers continue to prescribe metformin as single-agent therapy [11,12,28]. We also included the combined group as it has been hypothesized that use of both medications could offer comprehensive management by targeting distinct mechanisms. Fourth, contrary to several studies that have evaluated the effects of COCPs containing cyproterone acetate (a progestin not available in the US) [13], we selected a low-dose COCP with a third-generation progestin [3,30] aligning with current guidelines and allowing the generalizability of our findings. Fifth, given the high prevalence of psychological symptoms in PCOS and patient concerns regarding the impact of medications on these outcomes, we assessed changes in mood and quality of life scores and noted an improvement in all groups. Finally, we performed a detailed assessment of secondary outcomes including CVD risk phenotyping and included previously reported outcomes such as PCOS features [15] and medication side effects. Although the benefits of COCPs on gynecological and dermatological symptoms have been demonstrated [15], comprehensive evaluations of the effects of COCPs and metformin on these outcomes within this RCT further add to the strength of our study.

Our study limitations include a higher than anticipated drop-out rate that could have contributed to our findings of lack of significant trend in MetS prevalence at the end of study. Since there are no prior studies, to our knowledge, to compare the effects of first-line treatments for PCOS on MetS prevalence, our sample size and drop-out rates were based on smaller studies in heterogenous PCOS populations [10]. The medication side-effect related dropout rate was similar to that described in the general population, however, the COVID-19 pandemic may have contributed to the higher drop-out rate in all treatment groups related to participant decision. As discussed above, our choice of a low-dose COCP and inclusion of the hyperandrogenic patient population only may have resulted in the smaller-than-expected impact on MetS observed at the end of our study, suggesting that much larger studies will be needed in the future. Despite the observed dropout rate, our study has the largest sample size compared to published studies assessing impact of COCPs, metformin, and both on individual CVD risk factors and will thereby guide patient counseling [13]. We designed a 24-week study as changes in MetS components are typically evident within 3–6 months of initiating these medications [13,15,28]. However, it is possible that the impact of COCPs and metformin over longer durations may differ. Of note, in one study extending to 12 months, there were no significant differences reported in glucose, insulin, or HOMA-IR values with the same COCP used in our study [30]. Type 2 diabetes was a study exclusion as metformin is commonly used for its management, however, our inclusion of participants with prediabetes allows for the generalizability of our findings to the reproductive age PCOS population. Although our study population reflects the general US population, our findings may not be generalizable to all racial and ethnic groups. Our findings provide comprehensive information on impact of these medications on lipoproteins and support prior data on insulin sensitivity and glucose tolerance; however, we recognize that interpretation of multiple secondary outcomes is limited and potentially lack power to detect differences.

The COMET-PCOS trial addresses a major gap in the literature and challenges current practice patterns such as prolonged first-line use of metformin monotherapy over COCPs for managing PCOS symptoms and decreasing CVD risk. In individuals with hyperandrogenic PCOS and overweight/obesity, we report that monotherapy with a low-dose COCP offers a patient-centric approach as it did not increase the prevalence of MetS whilst decreasing serum androgens, hirsutism score, and android fat, protecting the endometrium and improving quality of life scores. Furthermore, lack of long-term data with clinically relevant outcomes and unfavorable side effect profile, should caution clinicians from using metformin routinely in combination with first-line COCP therapy in patients with PCOS. In this era of precision medicine, future studies should examine the long-term impact of COCPs, including their effect on atherosclerotic events and venous thromboembolism, in hyperandrogenic women with PCOS and preexisting metabolic syndrome. Future studies should examine a personalized approach related to the management of weight loss or insulin resistance by investigating the benefits of newer medications (e.g., glucagon-like peptide-1 receptor agonists) in this population.

## Supporting information

S1 CONSORT Checklist Hopewell S, Chan AW, Collins GS, Hróbjartsson A, Moher D, Schulz KF, and colleagues. CONSORT 2025 Statement: updated guideline for reporting randomised trials. BMJ. 2025; 388:e081123. https://dx.doi.org/10.1136/bmj-2024-081123. This is an Open Access article distributed under the terms of the Creative Commons Attribution License (https://creativecommons.org/licenses/by/4.0/).
(DOCX)

S2 SPIRIT Checklist Chan A-W, Boutron I, Hopewell S, Moher D, Schulz KF, and colleagues. SPIRIT 2025 statement: updated guideline for protocols of randomised trials. BMJ 2025;389:e081477. https://dx.doi.org/10.1136/bmj-2024-081477. Chan A-W and colleagues. This is an Open Access article distributed under the terms of the Creative Commons Attribution License (https://creativecommons.org/licenses/by/4.0/).
(DOCX)

S1 Text Study protocol with changes. (DOCX)

S2 Text Statistical analysis plan. (DOCX)

S3 Text Protocol deviation report. (DOCX)

S1 Table Eligibility criteria. (DOCX)

S2 Table Assessment of secondary outcomes. (DOCX)

S3 Table Baseline characteristics of participants related to additional secondary outcomes. (DOCX)

S4 Table Change in prevalence of metabolic syndrome (MetS) and its components between baseline (pre) and end of study visits (post) (unadjusted). (DOCX)

S5 Table Change in Secondary Outcomes from Baseline to End of Study Visits within each group and comparison between OCP, metformin, and combined groups (unadjusted). (DOCX)

S6 Table **(A)** Dietary intake based on ASA-24 data collected at week 12 and end of study Visit. **(B)** shows unadjusted values.
(DOCX)

S7 Table **(A)** Change in additional secondary outcomes from baseline to end of study within each group and comparison between OCP, metformin, and combined groups. **(B)** shows unadjusted values.
(DOCX)

S8 Table Serious adverse events. (DOCX)

S9 Table Change in MetS prevalence based on presence or absence of MetS at baseline. (DOCX)

## Abbreviations

ASA-24: automated self-administered 24-hour dietary recall
BMI: body mass index
BP: blood pressure
CI: confidence interval
COCPs: combined oral contraceptive pills
CVD: cardiovascular disease
DXA: dual-energy X-ray absorptiometry
GEE: generalized estimating equations
GI: gastrointestinal
HDL-C: high-density lipoprotein-cholesterol
IRB: Institutional Review Board
LDL-C: low density lipoprotein cholesterol
MC: mean change
MetS: metabolic syndrome
OR: odds ratio
PCOS: polycystic ovary syndrome
PCOSQ: PCOS-specific quality of life measures
RGM: geometric marginal means
TG: triglyceride
TRLP: TG rich lipoproteins
WC: waist circumference
