# Supplementary material for: Impact of combined hormonal contraceptives and metformin on metabolic syndrome in women with hyperandrogenic polycystic ovary syndrome and obesity: The COMET-PCOS randomized clinical trial
Source: PLoS Med. 2025 Dec 8;22(12):e1004662. doi: 10.1371/journal.pmed.1004662 (PMC12697981; doi:10.1371/journal.pmed.1004662)
Supplement: S1 Text — (DOCX) [file pmed.1004662.s003.docx]

The COMET-PCOS trial- Comparing the effects of Oral Contraceptive Pills versus Metformin in the medical management of overweight/obese women with Polycystic Ovary Syndrome

**(COMET-PCOS)**

Principal Investigator (1): Anuja Dokra, MD, PhD

Principal Investigator (2): Richard S. Legro, MD

Version 10.1

**Supported by:**

**R01 from the NIH**

**Study Intervention Provided by: No outside support**

**Sponsor of IND(IDE): Not Applicable**

Prepared by:

The University of Pennsylvania Women’s Health Clinical Research Center

Perelman School of Medicine

3701 Market Street

Philadelphia, PA 19104

(215) 615-0085

Last updated: November 30, 2021

**Protocol Subcommittee**

| Name | [E-mail](mailto:lamarc@mail.nih.gov) |
| --- | --- |
| Anuja Dokras, MD | [ADokras@obgyn.upenn.edu](mailto:ADokras@obgyn.upenn.edu) |
| Richard Legro, MD | [rsl1@psu.edu](mailto:rsl1@psu.edu) |
| Christos Coutifaris, MD | CCoutifaris@uphs.upenn.edu |

**Table of Contents**

[**1** **Study Synopsis 7**](#_Toc495299341)

[1.1 Objectives 7](#_Toc495299342)

[1.2 Hypothesis 7](#_Toc495299343)

[1.3 Patient Population 7](#_Toc495299344)

[1.4 Study Design 7](#_Toc495299345)

[1.5 Treatment 7](#_Toc495299346)

[1.6 Primary efficacy parameter 7](#_Toc495299347)

[1.7 Secondary efficacy parameters 8](#_Toc495299348)

[1.8 Statistical Analysis 8](#_Toc495299349)

[1.9 Anticipated time to completion 8](#_Toc495299350)

[1.10 Regulatory Compliance 8](#_Toc495299351)

[**2** **Study Objectives 9**](#_Toc495299352)

[2.1 Primary Aim 9](#_Toc495299353)

[2.2 Secondary Aim 9](#_Toc495299354)

[2.3 Tertiary Aims 9](#_Toc495299355)

[**3** **Background 10**](#_Toc495299356)

[3.1 Rationale 10](#_Toc495299357)

[3.2 Treatment of PCOS 10](#_Toc495299358)

[3.3 Metabolic Syndrome in PCOS 11](#_Toc495299359)

[3.4 Effect of OCP on MetS 11](#_Toc495299360)

[3.5 HDL-C function and lipoprotein analysis in PCOS 12](#_Toc495299361)

[3.6 Impact of OCP vs. metformin on HDL-C function and lipoprotein analysis in PCOS 12](#_Toc495299362)

[3.7 PCOS and adipocyte dysfunction 13](#_Toc495299363)

[3.8 OCP use in women with PCOS 13](#_Toc495299364)

[3.9 Metformin as an alternative treatment for women with PCOS 14](#_Toc495299365)

[3.10 OCP vs. metformin 14](#_Toc495299366)

[3.11 OCP vs. metformin vs. OCP + metformin 14](#_Toc495299367)

[3.12 Effects of OCP vs. metformin vs OCP + metformin on individual components of MetS 15](#_Toc495299368)

[3.13 Dyslipidemia 15](#_Toc495299369)

[3.14 Visceral adiposity 15](#_Toc495299370)

[3.15 Adipokine and cytokine secretion 16](#_Toc495299371)

[**4** **Study Design 18**](#_Toc495299372)

[4.1 Overview 18](#_Toc495299373)

[*4.1.1* *Treatment Design 18*](#_Toc495299374)

[*4.1.2* *Study Population 18*](#_Toc495299375)

[**5** **Selection and Enrollment of Subjects 19**](#_Toc495299376)

[5.1 Inclusion Criteria 19](#_Toc495299377)

[5.2 Exclusion Criteria 19](#_Toc495299378)

[5.3 Study Termination Criteria 20](#_Toc495299379)

[5.4 Study Enrollment Procedures 20](#_Toc495299380)

[*5.4.1* *Recruitment 20*](#_Toc495299381)

[5.5 Procedures for Tracking Sources of Subjects and their Disposition 21](#_Toc495299382)

[5.6 Obtaining Informed Consent 21](#_Toc495299383)

[5.7 Intervention Group Assignment 22](#_Toc495299384)

[**6** **Study Interventions 22**](#_Toc495299385)

[6.1 Interventions, Administration and Duration 22](#_Toc495299386)

[6.2 Concomitant Interventions 25](#_Toc495299389)

[6.3 Adherence Assessment 25](#_Toc495299390)

[**7** **Clinical and Laboratory Evaluations 25**](#_Toc495299391)

[7.1 Schedule of Evaluations 25](#_Toc495299392)

[7.2 Timing of Evaluations 26](#_Toc495299393)

[4. Review Adverse Events 28](#_Toc495299396)

[6. Collect medication 28](#_Toc495299397)

[4. Fasting blood draw and safety labs 29](#_Toc495299398)

[6. PCOSQ, CESD-R, STAI 30](#_Toc495299399)

[10. Review Adverse Events 30](#_Toc495299400)

[11. Collect Logs and medication 30](#_Toc495299401)

[12. Cognitive Questionnaires 30](#_Toc495299402)

[**8** **Study Risk and Benefits 30**](#_Toc495299403)

[8.1 Risk 30](#_Toc495299404)

[8.2 Protection Against Risks 32](#_Toc495299405)

[8.3 Potential Benefits of the Proposed Research to Human Subjects and Others 34](#_Toc495299406)

[8.4 Importance of the Knowledge to be Gained 34](#_Toc495299407)

[**9** **Statistical Considerations 35**](#_Toc495299408)

[9.1 General Design Issues 35](#_Toc495299409)

[9.2 Randomization 35](#_Toc495299410)

[9.3 Outcomes 35](#_Toc495299411)

[*9.3.1* *Primary Outcome Measurements 35*](#_Toc495299412)

[*9.3.2* *Secondary Outcome Measurements 35*](#_Toc495299413)

[9.4 Sample Size and Accruals 35](#_Toc495299414)

[*9.4.1* *Sample Size and Power Calculations 35*](#_Toc495299415)

[*9.4.2* *Statistical Analysis 36*](#_Toc495299416)

[*9.4.3* *Accrual 37*](#_Toc495299417)

[**10** **Data Collection, Monitoring and Adverse Experience Reporting 37**](#_Toc495299418)

[10.1 Records to be kept 37](#_Toc495299419)

[*10.1.1* *Maintenance/Retention of site records 38*](#_Toc495299420)

[*10.1.2* *Data Security 38*](#_Toc495299421)

[10.2 Adverse Event Reporting 38](#_Toc495299422)

[*10.2.1* *Serious Adverse Events 38*](#_Toc495299423)

[10.3 Data Monitoring 39](#_Toc495299424)

[10.4 Study Monitoring 39](#_Toc495299425)

[**11** **Human Subjects Protection 40**](#_Toc495299426)

[11.1 Institutional Review Board (IRB) Review and Informed Consent 40](#_Toc495299427)

[11.2 Subject Confidentiality 40](#_Toc495299428)

[11.3 Study Modification/Discontinuation 40](#_Toc495299429)

[11.4 Data and Safety Monitoring Board 41](#_Toc495299430)

[**12** **References 42**](#_Toc495299431)

[Figure 1. Prevalence of MetS in women with PCOS, geographically matched controls and aged matched NHANES population 10](file:///F:\Downloads\COMET-PCOS\2017.3.7_COMET-PCOS_Protocol.doc#_Toc476666316)

[Table 2: Randomization Fasting Blood Tests 27](#_Toc476666317)

**Acronyms**

| American College of Cardiology | **ACC** |
| --- | --- |
| Adverse Event | **AE** |
| Cardiovascular Disease | **CVD** |
| Data Monitoring Committee | **DMC** |
| Diabetes Mellitus | **DM** |
| Data Safety Monitoring Board | **DSMB** |
| Insulin Resistance | **IR** |
| Lifestyle Modification | **LSM** |
| Metabolic Syndrome | **MetS** |
| Oral Contraceptive Pills | **OCP** |
| Polycystic Ovary Syndrome | **PCOS** |
| Oral Glucose Tolerance Test | **OGTT** |

**Study Synopsis**

**Objectives**

To determine the effect of Oral Contraceptive Pills (OCP) verses Metformin verses OCP and Metformin on the prevalence of Metabolic Syndrome (MetS) and its components in overweight/obese women with Polycystic Ovary Syndrome (PCOS).

**Hypothesis**

OCPs increase the risk of MetS specifically by producing an atherogenic lipoprotein phenotype, increasing blood pressure and/ or body weight while metformin modestly decreases MetS risk by decreasing body weight and improving lipid phenotype. The combination of OCP and metformin (OCP, through lowering androgens, and metformin, through improvement in insulin sensitivity) will likely decrease the prevalence of MetS, thereby altering the risk profile for the development of diabetes and possible cardiovascular disease (CVD) in young women with PCOS.

**Patient Population**

The population will consist of 240 overweigh/obese women with hyperandrogenic PCOS, age 18-40 years old. Subjects must have a body mass index (BMI) between 25-48 kg/m². Subjects will be diagnosed with PCOS defined by the Rotterdam criteria.

**Study Design**

This will be a three-arm, double-blind, double-dummy, multicenter, prospective, randomized clinical trial comparing OCP + placebo vs. Metformin + placebo vs. OCP + Metformin on the prevalence of MetS in women with PCOS. This 6-month study will consist of a screening visit, followed by 5 study visits. No longer term follow-up is planned.

**Treatment**

The intervention will consist of randomizing subjects to one of three arms. Subjects will either be assigned to OCP + Placebo, Metformin + Placebo or OCP + Metformin. Metformin will be initiated in a step-up fashion using extended release pills as they are associated with fewer gastrointestinal side effects. Subjects will begin with one tablet of metformin every night for 5 days, eventually building up to 4 tablets every night, with the maximum dose of metformin being 2000 mg. In regards to OCP, previous randomized clinical trials (RCTs) have shown that 20mcg ethinyl estradiol/norethindrone 1.0 mg was well tolerated. The study will utilize a 20mcg OCP but a less androgenic third generation progestin (desogestrel 0.15mg) with potentially lesser impact on lipids and insulin sensitivity. All subjects with no menses the 3 months prior to screening will be given medroxyprogesterone acetate, at the screening visit, after a negative pregnancy test (in order to induce menses). Placebo pills will be administered to individuals randomized to OCP or metformin only in order to maintain study blinding. Subjects will undergo 6 in person study visits and life style modification counseling regarding diet and exercise. Patient contact will be made via the subject’s preferred contact method after randomization and at the end of each month when there is no in person visit to ensure study compliance with medications, keeping study logs and to review side effects.

**Primary efficacy parameter**

Determine the prevalence of MetS after randomizing to OCP, metformin or OCP+metformin for 6 months. MetS will be defined by NCEP ATPIII criteria as the presence of at least 3 of the following 5 criteria: TG≥150mg/dl, HDL-C<50mg/dl, BP≥130/≥85mmHg, WC>88cm and fasting glucose≥100mg/dl.

**Secondary/Tertiary efficacy parameters**

Assess change in HDL-C function, serum apolipoproteins, lipid particle size and number, body fat distribution, BMI, serum adipokines, HbA1c, glucose and insulin sensitivity, serum markers of inflammation, free fatty acids, androgens, quality of life parameters, cognitive testing and predictive factors for change in prevalence on MetS.

Subjects will have the option to consent to storage of their blood for future additional analysis as related to the disease of interest. No research/future analysis will take place, prior to gaining IRB approval.

**Statistical Analysis**

The primary analysis will use an intent-to-treat paradigm, wherein all randomized subjects are included according to their randomized treatment arm. Data will be summarized using descriptive statistics for continuous variables and frequency statistics for categorical variables. For the primary outcome of the presence of MetS at the end of the trial, logistic regression will be used with independent variables that include terms for the treatment arm and the 3 randomization stratification factors as covariates, with a contrast constructed to test for linear trend over the three treatment arms. For secondary continuous outcomes, linear mixed-effect models will be fit to assess differences between the treatment arms with respect to changes in these outcomes over time. The independent variables in the model will be treatment arm, time, the interaction of treatment and time and the 3 randomization stratification factors as covariates.

**Anticipated time to completion**

A total of 5 years (2017-2022) is anticipated. 3.5-year enrollment period (based on 3 subjects per site/month x 2 sites), 6-month treatment period followed by time for analyses and interpretation of the data.

**Regulatory Compliance**

A data safety and monitoring board, led by Dr. Kathleen Hoeger, has been established to ensure that patient safety and clinical study data and regulatory requirements are met regarding the Food and Drug Administration (FDA) code for federal regulations*. This trial is registered on* [*http://www.clinicaltrials.gov*](http://www.clinicaltrials.gov)

**Study Objectives**

**Primary Aim**

Our primary goal is to determine the effect of 6 months’ treatment with OCP vs. metformin vs. OCP + metformin on prevalence of MetS and its components in overweight / obese women. Implicit in the primary aim is clearly defining MetS, by NCEP ATPIII criteria as the presence of at least 3 of the following 5 criteria: TG≥150mg/dl, HDL-C<50mg/dl, BP≥130/≥85mmHg, WC>88cm and fasting glucose≥100mg/dl; and the goal of tracking safety of our interventions throughout the study (through safety lab evaluations, vital signs and diaries).

**Secondary Aim**

The study will assess secondary outcomes such as defining the effects of OCP (lowering androgens) verses metformin (improving insulin sensitivity) verses OCP + metformin on body fat distribution, glucose tolerance, adipokines and markers of inflammation.

1. Assess change in HDL-C function by measuring reverse cholesterol efflux capacity.
2. Compare changes in serum apoliproteins and lipid particle size and number.

**Tertiary Aims**

1. Compare changes in total and visceral body fat distribution (DXA) and serum adipokines in the 3 arms and correlate with changes in serum and androgens and markers of insulin sensitivity.
2. Identify changes in serum markers of inflammation and free fatty acids.
3. Compare changes in anxiety, depression and quality of life parameters in all 3 arms.
4. Compare changes in cognitive function scores in all 3 arms.
5. Comparing response to treatment with presence of DNA polymorphisms

**Background**

**Rationale**

**Treatment of PCOS**

Controversy exists regarding the optimal, long term management of overweight/obese women with polycystic ovary syndrome not attempting pregnancy. It is well known that these women are at increased risk for development of Type 2 diabetes, dyslipidemia, metabolic syndrome and possibly cardiovascular disease due to insulin resistance (IR) and maybe hyperandrogenism. In addition, the extent that racial differences in body fat distribution and dyslipidemia contribute to the differential burden of chronic disease in PCOS is unclear. Although preventive treatments with oral contraceptive pills or metformin are widely used, international surveys show prescribing patterns differ amongst treating physicians (i.e. gynecologist, endocrinologist, or pediatricians). In addition to different effects on menstrual cyclicity and hyperandrogenism, it is now clear that these different medical approaches also have varied metabolic effects possibly leading to adverse health consequences. For example, in a NICHD-funded study, the University of Pennsylvania, PCOS Center recently uncovered a greater than 2-fold increase in MetS after treatment with low dose OCP for 4 months in overweight/obese women with PCOS.

The few randomized clinical trials (RCTs) comparing OCP versus metformin use in overweight/obese women with PCOS show that with improvement in menstrual irregularity and hirsutism, OCP may increase metabolic risk by increasing triglycerides, blood pressure, body fat and weight. Metformin on the other hand, improves metabolic profile by decreasing serum glucose, insulin, triglycerides and body weight, although it has modest effects on menstrual irregularity and hyperandrogenism. These studies have several limitations including short study period, small numbers and the inclusion of Caucasian women only. Moreover, none have evaluated the effect of interventions on the composite risk of MetS or examined underlying mechanisms leading to change in metabolic risk. It is clear that there are no evidence-based recommendations for the optimum and comprehensive medical management of overweight/obese women with PCOS. The working hypothesis states that OCP, through lowering androgens, and metformin, through improvement in insulin sensitivity, will affect the prevalence of MetS thereby altering the risk profile for the development of diabetes and possible CVD in these young women.

Figure 1. Prevalence of MetS in women with PCOS, geographically matched controls and aged matched NHANES population


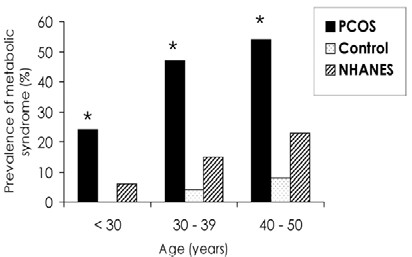


**Metabolic Syndrome in PCOS**

In 2005, we reported that the age-adjusted prevalence of MetS was higher in women with PCOS (47.3%, 95% CI 35.3-56.9%) compared to geographically matched controls (4.3 %, 95% CI 1.9-7.6%, p < .001). Even young women (< 30 years) had a high prevalence of MetS (Fig. 1). PCOS phenotypes with hyperandrogenemia have a significantly higher prevalence or MetS compared to controls1,2,3. African Americans (AA) in the general population have less visceral adipose tissue (VAT) and lower TG levels compared to Caucasians4,5and our recent data shows significantly lower visceral fat measured in AA women compared to white women with PCOS. (Table1).

| **Table 1. Differences in BMI-matched AA and White women with PCOS Mean (SD)** | **AA**  N=48 | **White**  N=48 | Difference of means | P  value |
| --- | --- | --- | --- | --- |
| Triglycerides mg/dl | 96 (66-120) | 117 (96.5-156.8) | 0.7 (0.6, 0.9) | 0.01 |
| % Fat in largest Visceral Fat region | 46.2 (5.4) | 48.7 (5.1) | -2.5 (-4.3, -0.7) | 0.01 |
| % Fat in middle Visceral Fat region | 41.2 (5.1) | 44.4 (4.9) | -3.2 (-4.8, -1.6) | <.001 |
| % Fat in inner-most Visceral Fat region | 39.1 (4.9) | 43.0 (4.9) | -3.9 (-5.5, -2.3) | <.001 |

Despite lower TG levels and less visceral adiposity, we found that AA women with PCOS ages 20-34 years have significantly increased risk of MetS compared to White women6 indicating racial disparity in cardiometabolic risk factors (Table 2). Inclusion of AA women in studies examining metabolic outcomes in PCOS is therefore critical and we will stratify our randomization by race.

| **Table 2. Racial Disparity in MetS** | N | **Metabolic Syndrome** | BMI≥30  kg/m2 | TG≥ 150  mg/dL | HDL  ≤ 50 mg/dL | BP≥ 130/85  mmHg | Glucose≥ 100 mg/dL |
| --- | --- | --- | --- | --- | --- | --- | --- |
| PCOS White | 244 | 22.6 % | 51.7% | 24.6% | 35.6% | 31.9% | 4.9% |
| **PCOS Black** | 67 | **40 %**** | **72.7% **** | **10.9%** * | **76.6% **** | **45.5% *** | **18.8%**** |
| NHANES white | 250 | 14.9% | 66.1% | 15.5% | 39.9% | 3.3% | 9.0% |
| NHANES black | 157 | 16.6% | 75.4%* | 9.9% | 42.9% | 10.6%* | 8.3% |

**Effect of OCP on MetS**

The OWL PCOS Study – We have recently completed an NIH funded study, OWL- PCOS at PENN and Hershey examining the effects of pretreatment with low dose OCP (20ug ethinyl estradiol) versus weight loss interventions for 16 weeks, on pregnancy rates in overweight/obese women with PCOS7. Surprisingly, the OCP arm showed a significant increase in MetS [OR=2.47; 95% CI 1.42, 4.27]; with no change in the intensive Lifestyle modification with pharmacotherapy arm [OR=1.18; 95% CI 0.63, 2.19)] or Combined arms [OR=0.72; 95% CI 0.44, 1.17]. The conversion rate to MetS was 28% in the OCP arm with significant increase in TG levels, and trend towards increase in BP and fasting glucose levels. Interestingly, we noted a significant decrease in VAT after treatment with OCP (Table 3) but an increase in glucose AUC. Our study suggests early onset metabolic dysfunction only in the OCP group, and this was attenuated in the Combined group with the addition of lifestyle modification associated with weight loss. We have previously shown that women with PCOS have a high risk for depressive disorders (OR 5.11 95% CI 1.26- 20.69; P<.03)8 and this risk persists over time9. In the OWL-PCOS study there was in fact an improvement in depressive symptoms and health related quality of life scores with OCP treatment7. These findings underscore the need for a clinical trial to comprehensively compare the effectiveness of therapeutic alternatives with specific focus on metabolic outcomes in addition to gynecological, dermatological and mood changes. Understanding the associated pathophysiological changes will add great value to this clinical trial.

| **Table 3. Effect of OCP on Visceral Adipose Tissue** | **Mean Change from Baseline (95% CI)** | **P-value** |
| --- | --- | --- |
| Fat Tissue in largest Visceral Fat region (g) | -66.3 (-127.1, -5.5) | 0.03 |
| Fat Tissue in middle Visceral Fat region (g) | -47.9 (-91.8, -4.1) | 0.03 |
| Fat Tissue in inner-most Visceral Fat region (g) | -40.0 (-78.4, -1.5) | 0.04 |

**HDL-C function and lipoprotein analysis in PCOS**

There is increasing evidence showing measures of HDL-C function are more useful than HDL-C levels as predictors of CVD risk10,11. In collaboration with Dr. Nehal Mehta, Chief of Inflammation and CV Medicine at NIH/NHLBI, we reported no difference in HDL-C levels but a significant reduction in cholesterol efflux capacity in PCOS women12 (Table 4). We found a significant negative association between testosterone levels and ApoA1 and HDL-C efflux. Women with PCOS also had significant elevation in the atherogenic particles, large VLDL and small LDL, independent of obesity12.

| **Table 4. Standard lipid and Lipoprotein Analysis** | PCOS n=124 | Controls n=67 | Difference of means | P value |
| --- | --- | --- | --- | --- |
| Total Cholesterol mg/dL | 192.5±37.9 | 189.7±34.5 | -2.8(-13.9, 8.2) | 0.6 |
| **HDL-C mg/dL** | **54.7 ±16.1** | **57.5± 17.9** | **2.8 (-2.2, 7.9)** | **0.2** |
| LDL-C mg/dl | 167.1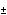50.8 | 154.9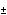43.9 | -12.7(-27.4,2.07) | 0.09 |
| Triglycerides mg/dl | 146.5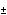92.9 | 112.2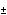69.9** | -34.3 (-60.4,-8.2) | 0.01 |
| **HDL-C function- Cholesterol efflux capacity (IQR)** | **0.96**  **(0.86-1.06)** | **1.05 (0.91-1.18)*** | **0.07 (0.17, 0.12)** | **0.005** |
| **NMR spectroscopy**  Large VLDL nmol/L | 4.04±3.7 | 2.37±1.73 | -1.6 (-2.7,-0.63) | 0.002 |
| VLDL particle size nm | 49.58±6.04 | 46.78± 5.28 | -2.7(-4.7,-0.88) | 0.004 |
| Small LDL nmol/L | 652.91 ±367.95 | 434.3± 280.7 | -218.57(-330,-106) | 0.0002 |

**Impact of OCP vs. metformin on HDL-C function and lipoprotein analysis in PCOS**

We have preliminary data using samples from the OWL PCOS study7 (OCP arm) and PPCOS1 study (metformin arm)111 showing significant increase in HDL-C efflux with both OCP and metformin treatments. (Table 5) However, this benefit is offset in the OCP group by a significant atherogenic effect, increased sLDL and VLDL particles.

| **Table 5. Impact of OCP versus metformin on lipids** | OWL PCOS-OCP arm n=34 | | PPCOS1-Metformin arm n=98 | |
| --- | --- | --- | --- | --- |
|  | Mean Change from Baseline (95% CI) | P-value | Mean Change from Baseline (95% CI) | P-value |
| **HDL-C efflux** | **0.10 (0.05, 0.16)** | **0.001** | **0.08 (0.04, 0.11)** | **<.0001** |
| HDL-Cmg/dl | 3.6 (0.1, 7.1) | 0.04 | 2.2 (-0.2, 4.5) | 0.07 |
| HDL-Particles | 4.2 (1.9, 6.5) | 0.0004 | 0.9 (-0.6, 2.5) | 0.23 |
| LDL-Cmg/dl | 11.7 (0.6, 22.7) | 0.04 | 4.5 (-2.9, 11.8) | 0.23 |
| **LDL-particles nmol/L** | **222.8 (98.6, 347.1)** | **0.001** | 51.4 (-31.4, 134.2) | 0.22 |
| **Small LDL nmol/L** | **190.1 (113.1, 267.1)** | **<.0001** | 13.3 (-38.4, 64.9) | 0.61 |
| **Large VLDL nmol/L** | **206.2 (90.5, 321.9)** | **0.001** | 47.8 (-29.3, 125.0) | 0.22 |

**PCOS and adipocyte dysfunction**

We have reported significantly lower serum adiponectin and higher leptin12, hsCRP13 and FFA levels12 in women with PCOS reflecting adipocyte dysfunction. Adipose tissue is a major source of FFA and inflammatory cytokines14,15which are drivers of CRP production in the liver 16,17.

Polycystic ovary syndrome (PCOS), which affects 5-15% of reproductive age women, has been linked to a high prevalence of cardiometabolic risk factors, namely type 2 diabetes (DM), obesity, dyslipidemia and hypertension2. The co-occurrence of these risk factors suggests the existence of metabolic syndrome (MetS), a clinically validated marker for identifying high-risk subjects predisposed to cardiovascular disease (CVD)18,19. The CVD risks of Mets and DM have been shown to be higher in women20,21and more importantly the association between MetS and CVD is shown to be more pronounced in younger women warranting aggressive preventive therapy14

. In our own studies, the prevalence of MetS in women with PCOS is significantly increased from a young age23 and is higher in the hyperandrogenic phenotype3. In a meta-analysis, including 2,256 PCOS women and 4,130 controls, the OR for MetS was 2.2 (95% CI 1.36-3.56) independent of obesity24.

Longitudinal studies in this population demonstrate a persistent increase in risk of DM, dyslipidemia and hypertension over a twenty-year period10, an increase in serum markers of CVD25,26and evidence of subclinical atherosclerosis27. In addition, perimenopausal and post-menopausal women with symptoms suggestive of PCOS have a higher risk of CV events28,29further linking PCOS to CVD later in life. Finally, it should be highlighted that, in addition to health consequences, these metabolic comorbidities along with gynecological and dermatologic concerns, contribute significantly to the financial (~$4.36 billion annually) and emotional burden of PCOS30,31. Collectively, these data support the notion that PCOS is associated with significant CV risk starting in early reproductive life and possibly continuing through menopause. It is striking that evidence based data on treatments for overweight/obese women with PCOS not seeking pregnancy is descriptive, limited and mixed. In fact, there are conflicting clinical guidelines from several medical societies32,33,34for first line management recommending either oral contraceptive pills (OCP) or metformin (insulin sensitizer). Surprisingly, the increased risk of MetS in this ‘at risk’ population as a consequence of taking these medications was unknown till recently. In the OWL-PCOS study, an NIH funded RCT21 we recently reported an increased risk of MetS (OR=2.47; 95% CI 1.42, 4.27) in obese women treated with OCP for 16 weeks. It is clear that comprehensive treatments addressing common complaints such as menstrual irregularity and hirsutism whilst improving metabolic risk are urgently needed. It is therefore proposed COMET PCOS, a randomized clinical trial to compare the effects of OCP vs. metformin vs. OCP+metformin on the risk of MetS in young overweight/obese women with PCOS**.**

**OCP use in women with PCOS**

OCP are effective first line therapy as they regulate menses and improve acne and hirsutism by significantly lowering bioavailable androgens in PCOS35,36. Decreasing androgens with OCP may have additional but relatively unexplored benefits in PCOS as elevated androgens may adversely affect insulin sensitivity, adipocyte function and fat distribution37. Some studies in women without PCOS suggest that OCP use is associated with glucose intolerance38,39hypertension40, elevated CRP levels41,42and dyslipidemia (especially hypertriglyceridemia) 43,44. Therefore, it can be hypothesized that OCP use may increase CVD risk especially given the high prevalence of obesity (50-80%) and hypertriglyceridemia in PCOS2. However, in a meta-analysis, treatment with OCP was associated with paradoxical changes in lipids, increase in triglycerides (TG) and high-density lipoprotein (HDL-C) levels, with no significant change in fasting glucose, low density lipoprotein (LDL-C) and insulin resistance (IR) in women with PCOS45. Further the impact of only one abnormal metabolic risk factor versus clustering of metabolic components on long term risk of DM and CVD in this population is unknown. None of these studies have examined MetS, a validated predictor of cardiometabolic risk, as a composite outcome. In the OWL-PCOS study pre-conception treatment of obese women with OCP resulted in an increased risk of MetS7.

**Metformin as an alternative treatment for women with PCOS**

Women with PCOS have a greater degree of insulin resistance (IR) compared to age and weight matched controls46 with defects in insulin-mediated glucose transport47,48 and GLUT4 production49,50. Given the role of hyperinsulinemia/IR in the development of hyperandrogenism and disordered folliculogenesis, metformin is the most extensively used insulin sensitizing drug and often first line treatment of PCOS51,52. In the general population, it decreases the risk of DM and CV events in adults53,54and in children and adolescents with IR or pre-diabetes metformin improves insulin sensitivity55 and may reduce BMI56. Metformin use in PCOS improves the frequency of menses and decreases androgens but less effectively compared to OCP making it less desirable as a first line agent57. However, metformin has a favorable impact on metabolic profile by decreasing fasting insulin and hsCRP levels, blood pressure53,58 and is associated with modest weight loss in women with PCOS 59,60,61,62. These studies, which are limited by size, randomization and short treatment duration, although suggestive of improvement in metabolic risk, are not conclusive. Lifestyle modification, another first line treatment in PCOS, is associated with poor adherence and sustainability. In a meta-analysis (608 women) use of metformin with lifestyle changes was associated with significantly lower BMI and improved menstruation compared to lifestyle changes alone63.

**OCP vs. metformin**

Very few RCTs have examined the effects of metformin versus OCP specifically in overweight/obese women with PCOS. A Cochrane systematic review (4 studies, 104 subjects) concluded that treatment with OCP is associated with improvement in menstrual pattern and serum androgen levels compared with metformin; but metformin use decreases fasting insulin and TG levels compared with OCP use64,65,66,67,68. All these studies were conducted in Europe using a progestin that is not available in the US (cyproterone acetate) limiting the generalizability of these findings. These studies do not provide evidence for any single treatment of choice and lack data on important clinical outcomes such as the development of MetS or DM69.

**OCP vs. metformin vs. OCP + metformin**

It is suspected that the combination of OCP+metformin may be an appropriate comparator to address gynecological, dermatologic and metabolic end points in PCOS. Only one clinical trial has included three arms namely OCP *vs* metformin *vs* OCP+metformin but included lean and obese women (n=65) with PCOS70 showing improvement in body fat distribution in the OCP+metformin arm71.

This clinical trial examined traditional CV markers, lacked mechanistic data and most importantly was not powered to provide clear recommendations. Overall these limited data present a clinical conundrum and underscore the need to answer important clinical questions; do OCP increase the risk of MetS in overweight/obese women with PCOS, can metformin mitigate these effects and therefore will the combination of OCP+metformin offer the best outcomes for improving menstrual regularity, decreasing hyperandrogenism and improving CV risk?

Further complicating matters, PCOS treatment practice patterns vary depending on the physician (i.e. pediatrician, gynecologist or endocrinologist) treating adolescent and adult women with PCOS not seeking fertility. In physician surveys metformin was noted to be preferentially used by Australian and European endocrinologists72,73while pediatricians and gynecologists practicing in the US frequently prescribed OCP as first line therapy74. Besides competing guidelines from several international medical Societies, 32,33,34,75 there are no formal recommendations for CV risk stratification prior to prescribing these medications1. Although the best therapeutic approach for MetS is lifestyle modification, such programs alone have high dropout rates necessitating pharmacotherapy to achieve and maintain weight loss76. It is therefore critical to identify appropriate medical therapy whilst avoiding medications that might alter the risk of MetS in this young but high risk population. The overall goal of COMET-PCOS is to provide practitioners with evidence for the selection of the most appropriate treatment for overweight/obese women with PCOS by carefully defining the clustering of metabolic risks and benefits associated with three interventions - OCP, metformin or OCP+metformin.

**Effects of OCP vs. metformin vs OCP + metformin on individual components of MetS**

There is some controversy regarding the predictive value of clustering of risk factors i.e. MetS versus the individual risk factors being independently associated with DM/CVD77. Therefore, evaluating the effects of OCP and/or metformin on each component of MetS can provide critical and novel mechanistic insights about the pathways altered by these treatments.

**Dyslipidemia**

Although PCOS is associated with low HDL-C levels and high TG1 and OCP use increases both HDL-C and TG levels, it is not clear how the interaction of these diametrically opposite alterations impacts the potential risk of coronary artery disease (CAD). While it may be beneficial that the estrogenic component of OCP increases HDL-C78,79, drugs such as the cholesterol ester transfer protein inhibitor torcetrapib and niacin that increase HDL-C, have failed to reduce CV events in clinical trials80,81. Two large studies recently indicated that HDL-C function may be a better marker of CVD outcomes independent of HDL-C levels10,11. We were the first to report significant reduction in cholesterol efflux capacity, a metric of HDL-C function, in PCOS12. While no gender differences have been described in HDL-C efflux82, it is unclear if hyperandrogenemia, IR or inflammation affects HDL-C function in PCOS83,84. Both hypertriglyceridemia, the commonest lipid abnormality in PCOS, and loss of function mutations of Apo C3 are also predictive of CHD85,86. In IR states, adipocytes increase free fatty acid (FFA) production14 and the liver subsequently increases TG and VLDL synthesis15 providing the substrate for increased atherogenic small (s)LDL particles87,88. Lipoprotein subclass profiles measured by nuclear magnetic resonance (NMR) spectroscopy are not fully apparent on a conventional lipid panel. sLDL and sHDL are associated with incident DM89 and IR90,91and are superior to standard LDL-C measurements in prediction of future CV events92. Of note, metformin therapy improved NMR measured lipoprotein profile in the Diabetes Prevention Study93**.** Therefore, as a secondary aim evaluating the effects of OCP, metformin and OCP+metformin on detailed lipid phenotyping will provide novel data to define the precise atherogenic impact of each study arm.

**Visceral adiposity**

Waist circumference is independently and strongly associated with DM, particularly in women94,95. Young women with PCOS have a twofold increased risk of DM9 independent of obesity96. It is however unclear if visceral adiposity in PCOS is similar97,98,99or increased 100,101,102 compared to controls. Studies examining change in body fat distribution after medical treatment in PCOS are also few and contradictory. While androgens may predispose to central body fat distribution103, OCP use, despite a decrease in androgens, has been associated in some studies with an increase in total and visceral fat mass104,105,106. On the contrary, metformin increases insulin-mediated glucose uptake in visceral adipose tissue, thereby increasing the re-esterification of FFA107,108. However, clinical studies in PCOS also show mixed results on the effects of metformin on visceral adiposity70,109,110. These controversies underscore the need to systematically evaluate change in visceral fat distribution with OCP and/or metformin use in order to understand its contribution to risk factors such as dyslipidemia, inflammation and altered glucose tolerance.

**Adipokine and cytokine secretion**

Visceral adiposity is associated with adipocyte dysfunction, characterized by inflammation and impaired adipokine secretion. Leptin secretion rises in parallel with fat expansion in adipocytes111 and elevated leptin levels have been described in PCOS compared to weight matched controls112. Adiponectin, on the other hand, which has insulin-sensitizing, anti-atherogenic and anti-inflammatory properties, and is inversely associated with MetS and independently predicts DM113, has been shown in a meta-analysis to be significantly lower in PCOS114. This observation was independent of obesity and testosterone, but associated with IR. The impact of metformin or OCP therapy on changes in adiponectin are mixed67,68,69,115,116 possibly related to differential actions on the liver and adipose tissue. Adipocytes also secrete inflammatory cytokines and a meta-analysis confirmed significantly higher levels of hsCRP in PCOS25. Metformin treatment is associated with decreased hsCRP117 and IL-6 levels in PCOS65,118. Precisely defining the effects of OCP and/or metformin use on adipokines and inflammatory cytokines will better assess the impact of these treatments on underlying pathophysiological processes associated with adipocyte dysfunction.

In summary, evaluation of MetS risk modification associated with treatments such as OCP and/or metformin in a high risk PCOS population is urgently needed and will have a major impact on current clinical practice.

**Implications for the COMET-PCOS Trial**

This project brings together a multidisciplinary exceptionally well-qualified team with demonstrated expertise in reproductive endocrinology, cardiometabolic risk assessment and inflammation and clinical trials to study two major health issues namely, PCOS and MetS. Our study includes four highly innovative components:

**First** Due to lack of evidence there is currently no consensus regarding the medical management of overweight/ obese women with PCOS. OCP and metformin, though currently in use, have differing treatment outcomes and safety profiles, resulting in conflicting guidelines for use in PCOS. COMET-PCOS will be the first 3 arm clinical trial to be adequately powered to provide evidence for single agent versus combination therapies for medical management of PCOS. It will have significant impact on current clinical practice paradigms much like the impact of the PPCOS1 trial (Clomid versus metformin versus clomid+metformin)111.

**Second** Based on our recent findings from OWL-PCOS we have included MetS as a composite endpoint to provide comprehensive assessment of early CVD risk in this young population.

**Third** We have included innovative mechanistic secondary aims and will apply novel concepts to the assessment of dyslipidemia, the commonest metabolic abnormality in PCOS. We will measure HDL-C function (cholesterol efflux capacity) and lipoprotein particle size and number using NMR spectroscopy to clearly define the atherogenic impact of the three therapeutic interventions.

**Fourth** Recognizing that there are racial disparities in metabolic components in PCOS, we will stratify the randomization in our primary aim by race. This will provide unique observations of potential importance.

**Study Design**

**Figure 2: COMET-PCOS Study Flowchart**

*
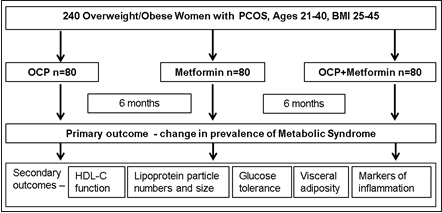
*

**Overview**

The flowchart (**Figure 2**) summarizes this study which will consist of medical treatments commencing with randomization at equal allocation (1:1:1) to three interventions OCP, metformin and OCP+metformin for a duration of 6 months. The primary outcome will be change in prevalence of Mets in each group. The secondary outcomes include assessment of HDL-C efflux, lipoprotein particle size and number, glucose tolerance, body fat distribution and measurements of markers of inflammation.

**Treatment Design**

This will be a three-arm, multi-center, prospective randomized trial of three types of medication treatment in overweight/obese women with hyperandrogenic PCOS who will be randomized to either OCP + placebo, metformin + placebo or OCP + metformin. All subjects will also receive lifestyle modification counseling regarding diet and exercise.

The appropriate study candidates will be recruited from the University of Pennsylvania (Penn) or Pennsylvania State University, Hershey Medical Center (PSU). Recruited subjects will meet the inclusion and exclusion criteria detailed below. Monitoring of this trial at both sites will be conducted by the Penn designated monitor for the Women’s Health Clinical Research Center at the University of Pennsylvania, with progress reports provided to the Data and Safety Monitoring Board (DSMB) no less than every twelve months in order to review trial progress and subject safety.

**Study Population**

Two-hundred forty (240) overweight/obese women with hyperandrogenic PCOS not seeking pregnancy, age 18-40 years, will be enrolled in the participating sites. The overall goal of the inclusion/exclusion criteria is to identify a population of healthy women who are between 25-48 kg/m². Subjects will have a history of androgen excess and chronic anovulation or PCO appearing ovaries. If existing medical records are used to verify inclusion or exclusion criteria, the site should keep a copy of these in the source documents. A one-month wash-out period (6 month wash out in the case of injectable hormonal contraceptive) will be required for medications prior to screening (most common OCP and metformin). Medications that subjects are required to wash-out are included in the MOP and questions can be directed to the Principal Investigator at each site.

**Selection and Enrollment of Subjects**

**Inclusion Criteria**

1. Women ≥ 18 to ≤ 40 years of age (at the time of screening), with hyperandrogenic PCOS.
2. Subjects will be diagnosed with PCOS defined by the most up to date Rotterdam criteria based on:
   1. Androgen excess

And

- 1. polycystic ovaries.

Or

- 1. A history of chronic anovulation or spontaneous periods.

1. BMI ≥ 25 kg/m² to ≤ 48 kg/m² obtained at screening visit.
2. In good general health according to the investigators discretion
3. Willing to avoid pregnancy for the duration of the study.

**Exclusion Criteria**

1. Current pregnancy or desire of pregnancy during course of study
2. Currently breastfeeding
3. Known 21 hydroxylase deficiency or any form of congential adrenal hyperplasia (CAH)
4. Untreated thyroid disease (TSH ≤0.45 mlU/mL and ≥ 4.5 mlU/mL)
5. Untreated hyperprolactinemia (2 Levels≥30 ng/ml at least one week apart)
6. Type 1 or type 2 Diabetes Mellitus currently receiving anti-diabetic agents, (subjects may wash out from metformin if taking the drug for another indication.)
7. Liver disease (AST/ALT≥2 times normal or a total bilirubin ≥2.5 mg/dL)
8. Renal disease (BUN≥30 mg/dL or serum creatinine ≥1.4 mg/dL)
9. Anemia (hemoglobin ≤10 mg/dL)
10. current history of alcohol abuse (≥ 22 drinks/week in the past 3 months)
11. Poorly controlled hypertension defined as average systolic blood pressure >= 150 mm Hg or average diastolic ≥100 mm Hg obtained on three measurements obtained 5 minutes apart. If treated, average systolic blood pressure ≥140 mm Hg or average diastolic ≥90 mm Hg
12. Patients with a history of, or suspected cervical carcinoma, endometrial carcinoma
13. TG≥250mg/dl
14. Current Use of lipid lowering or weight loss agents
15. Current use of hormonal contraceptives such as oral contraceptives, depo progestin, or hormonal implants
16. Participation in any study of an investigational drug or device or biological agent within 30 days
17. Suspected adrenal or ovarian tumor secreting androgens
18. Suspected Cushing’s syndrome
19. Bariatric surgery procedure in the recent past (≤12 months)
20. Absolute contraindications to the use of hormonal contraceptives or metformin (details in MOP)

22. subjects who are unable to comply with the study procedures, (In the opinion of the investigator, )

******Note:*** *the study will allow rescreening of subjects. When subjects are re-screened a new consent form will be signed and a new study number will be assigned.*

**Study Termination Criteria**

1. Development of diabetes as defined in the manual of procedures
2. Development of hypertension as defined in the manual of procedures
3. Unable to tolerate study medications due to side effects
4. Pregnancy (women found to be pregnant will be referred to prenatal care)
5. Miss more than 2 consecutive visits (unless excused by the PI)

**Study Enrollment Procedures**

**Recruitment**

We do not anticipate difficulty with the completion of this trial as our study team has demonstrated the ability to recruit a similar population in several previous trials (PCOS studies completed by UPenn and PSU: OWL-PCOS/PPCOS1 /PPCOS2). At both sites we have expert investigators in PCOS to improve external validity and to help overcome any possible recruitment barriers. Subjects recruited at UPenn are drawn from a four state area including Pennsylvania, New Jersey, Delaware, and Maryland. Dr. Dokras directs the Penn PCOS center which is located at 2 sites (Philadelphia city and in a suburban location - Radnor) and she sees approximately 1000 visits/year exclusively for PCOS. UPenn also has a high patient volume in Reproductive Endocrinology with 22,000 patient visits per year. Hershey Medical Center is the only academic medical center between Philadelphia and Pittsburgh with a large referral area. They have recruited subjects from areas as far away as Scranton/Wilkes Barre (90 miles) to the northeast, and Altoona (150 miles) to the West, Harrisburg (12 miles), York (30 miles), Lancaster (20 miles), State College (90 miles), Williamsport (90 miles), and Allentown (85 miles). The location of the two sites, University of Pennsylvania (UPenn) in an urban setting, and Hershey Medical Center (PSU) in a rural/suburban setting will allow us to recruit from a representative sample of the population.

*Clinical Practices of Investigators*

Women presenting to the clinical practices of Dr. Dokras, Dr. Mainigi, and Dr. Legro for consultation for PCOS (after clinical confirmation of their potential eligibility for admission into the study based on inclusion and exclusion criteria) will be approached to participate by the research coordinator or non-care providing physician. In the recruitment visit, study details including the intervention will be explained. Risks and benefits will be thoroughly discussed and consents given to the patient prior to any study procedures being performed. The drugs being utilized in this study are commonly used in current practice and may be familiar to the women interested in participating in the study.

*Hospital/Local Health Care Referrals*

Subjects will be recruited at each site from individual practice(s) of the investigators as noted above as well as faculty/resident clinics. Ongoing contact with practice and faculty members as well as with residents will be made by the investigators and coordinators, reminding them of the inclusion criteria, importance of the study, etc. In addition, the investigators will describe the study to members of other departments in the hospital, primarily family practice, medical endocrinology, and gynecology who also see and treat these patients. Contact with local physicians will be made and/or grand rounds will be given to disseminate information about the study. Letters will be mailed or emailed to potential subjects which provides details of the study and contact information of the study team (to allow for subject to reach out for more information). Penn Datastore as well as electronic medical record (EMR) recruitment tools such as SlicerDicer and best practice announcements (BPA) (and PSU, CHOP equivalents) may be utilized to provide a list of potential subjects based on clinical practice information.

*Referrals from Study Participants*

Study participants often refer friends, acquaintances and colleagues to be potential participants.

A subject referral-based payment structure will be added to increase recruitment. Prior study participants who have given their permission for re-contact that are not Penn Employees will be contacted to give them the opportunity to refer individuals to our study. For each individual referred who qualifies as eligible, the prior participant will get $25. The referral period will last until the study team determines the budget to support this type of referral is no longer available. The study team will inform the prior participants at that time of the ending of the referral-based payment program.

*Previous Study Participants*

The trial will be offered to women who have previously participated in clinical trials offered by the study team, who have consented to be contacted for future participation via their preferred contact method (after confirmation of their potential eligibility for admission into the study based on inclusion and exclusion criteria).

*Local Advertisements*

Advertisements will be placed in local newspapers and run on local television networks / radio near Penn and PSU and will be continued on a regular basis if response is good. SEPTA advertising through regional rail, Market-Frankford line and Broad Street Line has been a top source of recruitment in the pass and may be utilized again.

*Contact with PCOS support groups*

Contact will be made with both national and local support groups to spread information about the study through informational brochures and/or participation in local meetings if necessary.

*Web sites*

The study will be prominently displayed on the local (Penn, PSU, CHOP) web site. Additionally, each center will have a web page devoted to this study with general as well as contact information. Information will also be available at clinicaltrials.gov. Social Media and Craigslist will also be utilized, as this has been a proven source of recruitment in the past. For ads placed on social media, specifically Facebook and Instagram, they will be one-way adds and the study team will not directly interact with subjects on social media. The ad will highlight a contact number for the interested individual to call to discuss the study. Social media ads will be monitored by the University of Pennsylvania study coordinator to ensure they are properly being featured and not causing inappropriate comments. This monitoring will occur bi-weekly. If any problems arise with the social media ad they will be immediately removed however this is not anticipated as they are one-way ads. This type of advertising has been previously used for studies within the department and has been well received.

*IRB Approval*

It is expressly acknowledged that all informational material that could be construed to be advertising will be approved by the central IRB prior to dissemination.

**Procedures for Tracking Sources of Subjects and their Disposition**

We will track all contacts from subjects interested in the study. We will develop a pre-screening list that documents date and point of contact, eligibility based on telephone screening or survey, and follow-up if subject meets prescreening and is interested in further participation. The consent form may be mailed or emailed prior to the screening visit which is the next point of contact.

**Obtaining Informed Consent**

Once potential women have been prescreened, they will be referred to the site clinical coordinator or his/her designee for a screening visit. The consent process will be conducted according to each site’s standard operating procedures.

Inclusion and exclusion criteria will be reviewed. After the study has been completely explained to the woman, she will be given the informed consent documents to review. Some individuals may wish to complete the informed consent process at the time of this discussion. In these cases, the informed consent documents will be signed once all questions are resolved. In other cases, the subjects may wish to take the consent forms home for further consideration. In these cases, the coordinator will confirm the woman’s willingness to be contacted, and set up a tentative timeframe to be back in touch with the subjects. The consent can be signed once all questions have been answered to the satisfaction of the potential subject. A signed informed consent document, approved by the central IRB, will be confirmed on all subjects prior to the baseline evaluation.

In order to be eligible for enrollment and randomization, the woman must be confirmed to meet all inclusion and exclusion criteria described above.

**Intervention Group Assignment**

After screening is completed the information will be uploaded in RedCAP electronically, and randomization will be performed by IDS at the time of the randomization visit. Subjects and study coordinators will not be informed of their treatment assignment.

**Study Interventions**

**Interventions, Administration and Duration**

Study Medication: All subjects will be randomized to receive either OCP + placebo, Metformin + placebo or OCP + Metformin for 24 weeks. All medications will be self-administered. Once eligibility has been confirmed and subjects have been randomized, the site staff will distribute appropriate medications during each visit. The subject will receive enough pills to last until the next visit (additional pills for each arm will be distributed at the start of the study for situations if/when the visit window must be utilized; subjects will receive appropriate instructions regarding the extra medication). Subjects will be required to wash out of any excluded medications prior to enrollment in the study.

Medication Compliance: Medication bottles will be returned at visits 3 through 6 unless a remote visit is being conducted at the discretion of the PI. IDS will perform pill reconciliation during these visits to measure medication compliance. Medication compliance can also be assessed by reviewing the returned subject diaries. Compliance is assessed during the monthly visits on site or remote contact with the subject. Non-compliant subjects will be reminded of the importance of taking the medication properly.

Life Style Modification (LSM): In addition to taking study medication, all subjects will receive counseling (through the use of handouts) about diet and exercise at the randomization and 16 week visit. The diet is based on the lifestyle modification protocol of the look AHEAD study and POWER-UP trial. The study will follow the Diabetes Prevention Program recommendations for increasing physical activity. A similar protocol was utilized in OWL-PCOS and similar written materials will be provided to all subjects in the current trial. Assessment of dietary intake will be based on NCI created automated self-administered 24-hour (ASA24) food recall tool. This will allow us to access variability in dietary intakes in the 3 arms of the study.

Cognitive Testing: All subjects will undergo cognitive functioning testing via the NIH Toolbox, a comprehensive set of neuro-behavioral measurements that quickly assesses cognitive, emotional, sensory, and motor function. Audio-recording of cognitive testing is optional (all recordings will be deleted after transcription). The NIH Toolbox is assessing cognition for subject (age3-85), thus eliminating a ceiling effect. Staff will be trained to administer the tests. Training is performed online via the NIH Toolbox website. Any additional training or oversite will be done by a Clinical Neuropsychologist. During testing staff will audio record cognitive testing with subjects to verify answers given in a timely matter. All audio recordings will be consented for by the subject prior to recording and will remain on file for less than 24 hours at which point they will be deleted. One patient recording from each certified staff member will go to the Clinical Neuropsychologist to verify standardization. A description of the neuropsychological assessments includes:

Neuropsych assessments:

- *Controlled Oral Word Association (COWA):* subjects must spontaneously name as many words as possible beginning with a given letter within a 60 second span.
- *Category fluency:* subjects must spontaneously name as many objects as possible that fit a given category within a 60 second span.
- *Logical memory:* subject is read the first story and recalls as many details as possible. The subject is then read a second story and must recall as many details as possible about that story; second story and recall is repeated. Following a delay of 25-35 minutes, the subject must again recall as many details of each story as possible.
- *Digit Span:* subject is read a string of numbers and must repeat the numbers back to the examiner in either the exact same order (DS forward) or in reverse order (DS backward). Number strings increase in length by one number with each successful turn, and discontinued when patient makes an error. Score is the longest string a patient can successfully repeat back.
- *Digit symbol substation:* Testing page shows a key at the top, which contains the numbers 1-9, each paired with a special symbol. Below the key, subjects must fill in the numbers that match a grid of special symbols as quickly as possible, based on the key above.
- *Rey-Osterreith Figure:* subject copies the complex figure from the stimulus page. The stimulus is removed and the patient then immediately redraws as much of the figure as they can (immediate recall score).
- *Pegboard:* subject is tested with both dominant and non-dominant hands on how quickly they can fill in a pegboard. Each peg has a groove, which must be perfectly matched with the groove of the hole in order to fit. Score based on time to complete.
- *Trail making:* Numbers are scattered over a page and the subject must connect the numbers in numerical order; score derived from both number of errors and time to complete. Executive function can be tested by requiring patients to connect numbers in an alternating fashion between two colors, i.e. yellow 1-pink 2-yellow 3- pink 4, etc. Score still derived from both errors and time to complete.
- *Visual Puzzles:* Subjects are shown a 2D item that they are to imagine as a puzzle made from 3 pieces. The subject indicates which 3 pieces fit next to each other without stacking to make the given item. Each item has a 30 second time limit, with a discontinuation rule of 3 errors in a row.  The score is based on time and errors.

Urine Pregnancy Test: A urine pregnancy test will be performed for all subjects at every visit. If a positive result is found, the subject will be informed by site staff of positive result and offered resources regarding prenatal care or alternative options. In the case of a positive pregnancy result, subjects will be withdrawn from the study. In the case of a subject becoming pregnant, the subject will have the choice to consent to allow the study team to track until outcome of the pregnancy.

Vital Signs and Biometrics**:** Vital signs and biometrics for this study include but are not limited to, measuring height and weight, blood pressure, pulse, waist and hip circumference. Vital signs and biometrics will be collected at every visit except height which will only be recorded at the screening visit.

Blood pressure and pulse will be measured in sitting position at all visits after the subject has rested for at least five minutes. Three blood pressure measurements will be taken 5 minutes apart and then averaged.

Laboratory Evaluations: Blood will be collected at Screening, Randomization, week 16 and week 24 visits (early termination visit). Subjects will be required to be fasting prior to Screening, Randomization, week 16 and week 24 visit (nothing to eat or drink besides water for a minimum of 8 hours prior to blood draw). Subjects who have used hormonal contraception must wait at least 30 days prior to the collection of screening and fasting blood l tests. Screening blood tests that were completed within the last year can be substituted for laboratory tests collected at screening visit.

Transvaginal Ultrasound: Transvaginal ultrasound examinations will be performed at screening if indicated, randomization and 24 week visit (or early termination visit). The transvaginal ultrasound examinations will be performed according to the standard procedure for the center and include finding regarding the uterus, the endometrium and the ovaries. If a transvaginal probe is not tolerated, then transabdominal ultrasound may be used. The same procedures were used in the OWL-PCOS study and are well known by the centers.

F-G Score: The Ferriman-Gallwey score is a measure for quantifying hirsutism in women. The Primary Investigator (or designee, which may be the subject) will assess each subject’s hirsutism using the Ferriman-Gallwey scale at Screening, Randomization (if applicable and/or necessary) and 24 week visit. During the assessment for hirsutism, the PI (or designee) will also assess the subject’s acne.

DXA: DXA scans will be completed on each patient at Randomization Visit and 24 week visit. The scanning will include a whole body scan, the lumbar spine and both hips.

Oral Glucose Test: OGTT will be performed at the randomization and at 24 week visit. Prior to the OGTT subject must fast overnight for minimum of 8 hours. If subject is not fasting, the blood work must be rescheduled. Patients will receive 75 g of an oral glucose solution at time zero (0) and blood will be sampled every 30 minutes for 2 hours for glucose, and insulin levels.

PCOSQ: The PCOSQ is a self-administered questionnaire for measuring health-related quality of life in women with PCOS. The PCOSQ will be administered to subjects at Randomization and 24 week visit.

STAI: The STAI (State-Trait Anxiety index) is a questionnaire for accessing trait anxiety and state anxiety. The STAI will be administered to subjects at Randomization and 24 week visit.

CES-D: The CES-D is a screening test for depressive symptoms. The CES-D will be administered to subjects at Randomization and 24 week visit.

FSDS-R: The FSDS-R (The Female Sexual Distress Scale-Revised) is a screening questionnaire for measuring sexually related personal distress in women with Female Sexual Dysfunction (FSD)

FSFI: The FSFI (Female Sexual Function Index) is a brief questionnaire measure of sexual function in women. It was developed for the specific purpose of assessing domains of sexual function (e.g. sexual arousal, orgasm, satisfaction, pain) in clinical trials.

ASA24: The Automated Self-Administered 24-Hour Dietary Assessment Tool (ASA24). This 24-hour dietary recall captures detailed information about all foods, drinks, and supplements (including vitamins, minerals, herbals, and other dietary supplements) you consumed from midnight to midnight. You will be asked to complete this at 12 weeks and 24 weeks.

**Concomitant Interventions**

No other interventions, other than those specified in the protocol are allowed to treat PCOS or weight management during participation in the study.

**Adherence Assessment**

Adherence to the interventions will be monitored by the use of medication reconciliation, diary compliance and by monthly visits on site or contact with the subject.

**Clinical and Laboratory Evaluations**

**Schedule of Evaluations**

The table below summarizes the clinical and laboratory evaluations during the trial. A description of the visits follows. Note, prior to the screening visit, subjects may prescreen for potential eligibility.

**Table 6: Study Visits and Procedures**

| **Study Visit Schedule** | **Screening (half of the visit as a remote option)** | **Randomization** | **Randomization Patient Contact (PC)** | **4 Week Visit (remote option)** | **8 Week Visit (remote option)** | **12 Week PC** | **16 Week Visit** | **20 Week PC** | **24 Week Visit** | **Early Termination Visit (> or equal to 12 weeks of medication)** | **End of study PC** |
| --- | --- | --- | --- | --- | --- | --- | --- | --- | --- | --- | --- |
| Sign Informed Consent | x |  |  |  |  |  |  |  |  |  |  |
| Medical History Questionnaire | x |  |  |  |  |  |  |  |  |  |  |
| Screening labs | **x*** |  |  |  |  |  |  |  |  |  |  |
| DNA Sample |  | x |  |  |  |  |  |  |  |  |  |
| Safety Labs | **x*** | x |  |  |  |  | x |  | x | x |  |
| Menstrual History | x | x |  | x | x |  | x |  | x | x |  |
| Urine Pregnancy Test | x | x |  | x | x |  | x |  | x | x |  |
| Vitals and Biometrics | x | x |  | x | x |  | x |  | x | x |  |
| Hirsutism Assessment | x | x |  |  |  |  |  |  | x | x |  |
| Transvaginal Ultrasound | **x*** | x |  |  |  |  |  |  | x | x |  |
| Fasting labs |  | x |  |  |  |  | x |  | x | x |  |
| OGTT labs |  | x |  |  |  |  |  |  | x | x |  |
| DXA Scan |  | x |  |  |  |  |  |  | x | x |  |
| Lifestyle Modification |  | x |  |  |  |  | x |  |  |  |  |
| ASA 24 |  |  |  |  |  | x |  |  | x | x |  |
| Acne Assessment |  | x |  |  |  |  |  |  | x | x |  |
| HRQOL – PCOS, STAI, CES-D, FSDS-R, FSFI & Cognitive testing Questionnaires |  | x |  |  |  |  |  |  | x | x |  |
| Randomization |  | x |  |  |  |  |  |  |  |  |  |
| Dispense Medication/Log |  | x |  | x | x |  | x |  |  |  |  |
| Collect  Medication/Log |  |  |  | x | x |  | x |  | x | x |  |
| Review Concomitant Medications | x | x | x | x | x | x | x | x | x | x | x |
| Review Adverse Events |  |  | x | x | x | x | x | x | x | x | x |

*As Needed

**Timing of Evaluations**

**7.2.1 Screening visit:**

1. Obtain informed, signed consent from subject.

- Subject must specifically be made aware of potential drug side effects and adverse outcomes.

* Remote option for e-consent via REDCap, utilization of this option at PI discretion

2. Complete medical assessment.

- At the Screening Visit, a detailed general medical history for the former 6 months (longer in case of gynecological relevance) and history of concomitant diseases or interventions is to be reviewed and to be documented.

* Remote option for completion, utilization of this option at PI discretion

1. Must obtain and record menstrual history.
   - - Determine need for medroxyprogesterone acetate (if subject has had no menses in the last 3 months)

* Remote option for completion, utilization of this option at PI discretion

4. Perform urine pregnancy test.

5. Record vital signs and biometrics

6. Blood draw for both screening labs (as needed) and safety labs (as needed)

1. Review Hirsutism (F-G) Score

* Remote option for completion, utilization of this option at PI discretion

8. Transvaginal Ultrasound (if indicated)

9. Review electronic Medical Record (if available)

- If subjects are patients through the University of Pennsylvania Health System or Hershey Medical Center, electronic medical records will be reviewed to verify eligibility. Screening blood laboratory tests that were completed within the last year can be substituted for laboratory tests collected at screening and randomization visit.

**Table 7: Screening Blood Tests**

| ***Category*** | ***Tests*** |
| --- | --- |
| *Safety Labs*** | Complete metabolic panel, lipid profile |
| *Screening Labs* | TSH*, Prolactin*, HbA1c*, CBC*, 17OH Progesterone^, DHEAS^, Total Testosterone^#^, |

*If drawn within 1 year of screening, eligible for use to determine eligibility criteria.

^ If drawn within lifetime prior to screening, eligible for use to determine eligibility criteria.

^#^ If FG≥6 (≥2 for women of Asian descent) do not need to draw lab. Eligibility criteria can be determined without this lab value.

** If drawn within 2 months of screening, eligible for use to determine eligibility criteria. These safety labs must be drawn at Randomization visit if not drawn at Screening visit.

**7.2.2 Randomization Visit:**

1. Review menstrual history
2. Complete urine pregnancy test
3. Record vital signs and biometrics
4. Complete fasting blood draw and Safety labs (as needed)
5. Optional whole blood draw for DNA testing
6. Start the oral Glucose Tolerance Test (this may be first since the subjects are fasting)
7. Review Lifestyle Modification Counseling
8. Complete PCOSQ, CESD-R, STAI, FSDS-R & FSFI
9. Cognitive testing
10. Complete DXA scan
11. Transvaginal Ultrasound (if indicated)
12. Randomize into study arm
13. Dispense Logs/Medications
    - Review medication instructions

*For more detailed information regarding the order in which study procedures should occur, please refer to the Manual of procedures.

****Complete patient contact with subject two weeks after this visit.***

**Table 1: Randomization Fasting Blood Tests**

| ***Category*** | ***Tests*** |
| --- | --- |
| *Fasting blood Labs* | Total and free T, Apo A, Apo B, Apo.C3, hsCRP, FFA, IL-1,IL-6,1L-10, hsCRP, NMR lipoprotein analysis, HDL-C efflux, AMH, SHBG, TNF alpha, Estradiol |

**7.2.3 Follow- Up Visits**

Follow up Visits will be scheduled as shown in Table 6. Initial visits are only one month apart to allow us to trouble shoot any side effects especially while increasing the metformin/placebo tablets to the full dose and ensure adherence with the protocol. Urine pregnancy test will be checked, adverse events assessed, study logs reviewed, and new medications and study logs will be dispensed at each visit. After randomization and at 12 and 20 weeks adverse events, menstrual history, medication compliance will be reviewed via patient contact. Patient contact has also been placed 2 weeks after randomization to ensure patients are taking the medication properly and have experienced no adverse side effects.

**Table 9: Follow- Up Visits**

| Visit | Ideal Date | Lower Window  (- 1 week) | Upper Window  (+ 1 week) | Out of Window Limit  (+/- weeks) |
| --- | --- | --- | --- | --- |
| 2 Weeks* | 2 weeks | 1 week | 3 weeks | 0 / 4 weeks |
| 4 Weeks | 4 weeks | 3 weeks | 5 weeks | 2 / 6 weeks |
| 8 Weeks | 8 weeks | 7 weeks | 9 weeks | 6 / 10 weeks |
| 12 Weeks* | 12 weeks | 11 weeks | 13 weeks | 10 / 14 weeks |
| 16 Weeks | 16 weeks | 15 weeks | 17 weeks | 14 / 18 weeks |
| 20 Weeks* | 20 weeks | 19 weeks | 21 weeks | 18 / 22 weeks |
| 24 Weeks | 24 weeks | 23 weeks | 25 weeks | 22 / 26 weeks |
| 25 Weeks* | 25 weeks | 24 weeks | 26 weeks | 23 / 27 weeks |

* Patient contact

**7.2.4 Week 4 Follow- Up Visit**

* Remote option for completing visit, utilization of this option at PI discretion

1. Collect menstrual log and review medical history
2. Perform urine pregnancy test
3. Record Vital signs and Biometrics
4. Review Adverse Events
5. Collect Logs/Medication
6. Dispense Logs/Medications

**7.2.5 Week 8 Follow- Up Visit**

* Remote option for completing visit, utilization of this option at PI discretion

1. Collect menstrual log and review medical history
2. Perform urine pregnancy test
3. Record Vital signs and Biometrics
4. Review Adverse Events
5. Lifestyle Modification Assessment

Instruction to subject that the ASA 24 (online diet diary recall assessment) to be completed prior to the 12-week patient contact.

1. Collect Logs/Medication
2. Dispense Logs/Medications

**7.2.6 Week 12 patient contact**

This contact will be made to ensure subject compliance with medications, keeping study logs and to review any side effects.

**7.2.7 Week 16 Follow- Up Visit**

1. Collect menstrual log and review medical history
2. Perform urine pregnancy test
3. Record Vital signs and Biometrics
4. Fasting optional blood draw and safety labs
5. Lifestyle Modification Counseling
6. Review Adverse Events
7. Collect Logs/Medication
8. Dispense Logs/Medications

**7.2.8 Week 20 patient contact**

This contact will be made to ensure subject compliance with medications, keeping study logs and to review any side effects. Instruction to subject that the ASA 24 (online diet diary recall assessment) to be completed prior to the 24-week visit

**7.2.9 Week 24 Final Follow- Up Visit or Early Termination Visit (**≥ **to 12 weeks of being on study medication)**

1. Collect menstrual log and review medical history
2. Perform Urine pregnancy test
3. Record vital signs and biometrics
4. Review Hirsutism (F-G) Score/ Acne Assessment

1. Fasting blood draw and safety labs
2. Complete an oral Glucose Tolerance Test
3. PCOSQ, CESD-R, STAI
4. Review Hirsutism (F-G) Score/ Acne Assessment
5. Complete DXA scan
6. Obtain a Transvaginal Ultrasound (TVU)
7. Review Adverse Events
8. Collect Logs and medication
9. Must obtain and record menstrual history
   - - Determine need for medroxyprogesterone acetate (if subject has had no menses during the course of this study)

**7.2.10 Week 25 patient contact**

This contact will be made one week after patient has completed taking the medication to review any side effects and answer any patient questions. This patient contact will close out the study adverse events.

**Study Risk and Benefits**

**Risk**

The risks of these studies include the risks of the study procedures, and the risk of the study interventions. These will be discussed separately below.

Risks of Study Procedures

These risks include the risk of phlebotomy, oral glucose tolerance test and DXA. The risks of phlebotomy include pain at the phlebotomy site, bruising and rarely bleeding, and potentially iatrogenic anemia. Risks of the oral glucose tolerance test include the risks of multiple phlebotomy or in some cases the risk of insertion of an IV. The risks of IV insertion include the risks of phlebotomy, and additionally the induction of anemia, and infection at the site. Glucose ingestion can cause nausea and in rare instances vomiting upon ingestion. We have never experienced an allergic reaction to the oral glucose solution, though this is possible. Some subjects also may experience an episode of hypoglycemia after the test which can present with increased heart rate and anxiety. In the most severe cases, fainting can occur. The DXA scan will involve exposure to radiation, on average 2-3 milliroentgens of radiation during the procedure. The average amount of radiation that a person would receive from a DXA scan is less than 1% of the background radiation experienced from living in the Philadelphia area for one year, for example.

Risks of Study Interventions

Oral Contraceptive Pill - OCP used in this study is low dose (20micrograms ethinyl estradiol), which may cause bloating, nausea, breast tenderness, mood changes, weight change and headaches. Common side effects include breakthrough bleeding and amenorrhea. OCP are also associated with increased risk for cardiovascular disease, especially venous thromboembolism and stroke. OCP can also cause hypertension and alter blood lipid and glucose levels. Interactions with drugs such as phenobarbital, phenytoin, and rifampin increase the metabolism of oral contraceptives, thus decreasing the effectiveness as a method of birth control. Table lists all side effects recorded in women in the OCP arm of the recently completed OWL-PCOS study (n=49). Roughly same numbers are expected in this current study.

| **OWL-PCOS study**  **OCP side effects** | **Number of**  **subjects (%)** | **OCP side effects** | **Number of**  **subjects (%)** |
| --- | --- | --- | --- |
| Headache | 14 (28.6) | Dizziness/Vertigo | 2 (4.1) |
| Upper Respiratory Infections | 8 (16.3) | Fatigue | 2 (4.1) |
| OWL-PCOS | N (%) | Side effects | N (%) |
| Nausea/Vomiting | 7 (14.3) | Back Pain | 1 (2.0) |
| Breast Pain | 10 (20.4) | Acne | 1 (2.0) |
| Abdominal Pain | 1 (2.0) | Chest pain | 1 (2.0) |
| Dysmenorrhea | 8 (16.3) | Dental abscess | 1 (2.0) |
| Constipation | 1 (2.0) | Hot flushes | 1 (2.0) |
| Abnormal uterine bleeding | 4 (8.2) | Elevated BP w/o hypertension | 1 (2.0) |
| Mood Swings | 3 (6.1) | Gastroenteritis | 1 (2.0) |
| Gas/Bloating | 2 (4.1) | Otitis media | 1 (2.0) |
| Musculoskeletal Pain | 3 (6.1) | Anemia | 1 (2.0) |
| Vaginitis/Vulvitis | 4 (8.2) | Visual Changes | 1 (2.0) |
| Pelvic Pain | 3 (6.1) | Conjunctivitis | 1 (2.0) |
| Bunion | 1 (2.0) | Hemorrhoids | 1 (2.0) |
| Myalgia/myositis | 1 (2.0) | Bleeding, rectal | 1 (2.0) |
| Fever | 1 (2.0) | Cholelithiasis | 1 (2.0) |
| Sweating excess | 1 (2.0) | Galactorrhea | 1 (2.0) |

Treatment with metformin XR - Gastrointestinal symptoms (diarrhea, nausea, vomiting, abdominal bloating, flatulence, metallic taste in the mouth and anorexia) are the most common reactions to metformin XR and approximately 30% more frequent in women taking metformin XR compared to placebo. These symptoms are generally transient and resolve shortly after initiation of treatment. These symptoms will be managed with a step down decrease in the daily metformin XR dose (1/2 to one tablet per day per week) until symptoms resolve. These symptoms alone will not be a reason for withdrawal from the study unless a patient is unable to tolerate any dose of the medication. There is a small risk of lactic acidosis among women taking this medication. This most commonly occurs in patients with poorly controlled diabetes and impaired renal function. Vitamin B12 deficiency has also been reported in metformin-treated adults with T2DM. Women with these pre-existing medical problems will be excluded from the study as per the exclusion criteria. The Table below shows adverse events from metformin use in women with PCOS in the PPCOS1 study and the same results are expected in this current study.

| **PPCOS1 metformin Side effects** | Metformin(N=208) |
| --- | --- |
| Blood and lymphatic system disorders | 1/208 (0.5%) |
| Cardiac disorders | 1/208 (0.5%) |
| Congenital, familial and genetic disorders | 0/208 (0.0%) |
| Ear and labyrinth disorders | 3/208 (1.4%) |
| Endocrine disorders | 2/208 (1.0%) |
| Eye disorders | 3/208 (1.4%) |
| Gastrointestinal disorders | 177/208 (85.1%) |
| General disorders and administration site conditions | 61/208 (29.3%) |
| Immune system disorders | 3/208 (1.4%) |
| Infections and infestations | 43/208 (20.7%) |
| Injury, poisoning and procedural complications | 3/208 (1.4%) |
| Investigations | 6/208 (2.9%) |
| Metabolism and nutrition disorders | 37/208 (17.8%) |
| Musculoskeletal and connective tissue disorders | 29/208 (13.9%) |
| Nervous system disorders | 108/208 (51.9%) |
| Pregnancy, puerperium and perinatal conditions | 0/208 (0.0%) |
| Psychiatric disorders | 41/208 (19.7%) |
| Renal and urinary disorders | 7/208 (3.4%) |
| Reproductive system and breast disorders | 84/208 (40.4%) |
| Respiratory, thoracic and mediastinal disorders | 24/208 (11.5%) |
| Skin and subcutaneous tissue disorders | 19/208 (9.1%) |
| Surgical and medical procedures | 0/208 (0.0%) |
| Vascular disorders | 33/208 (15.9%) |

**Protection Against Risks**

Risks of adverse events will be reduced by the study personnel and the investigators regularly monitoring participants’ progress, by oversight of the local IRBs and the DSMB (see section below).

Clinical staff at each site - Our reproductive endocrinology groups at UPenn and PSU staffs a daily infertility clinic that meets every day (365 days a year). The staffing doctor also covers inpatient responsibility and research aspects of our service. The subjects at UPenn will be seen in the WHCRC which is on the same floor and adjacent to the REI practice site. The daily clinic at PSU meets just below the GCRC where the subjects are studied. At both sites a physician is available at a moment’s notice to see a subject. Therefore, the patients participating in the trials will have access to same day consult or visit with an investigator involved in the study, regardless of weekends and holidays, for any potential problem that arises in a study. We have utilized this system successfully for a wide variety of clinical studies for over 10 years.

Review at research meetings - Both groups at UPenn and PSU currently hold weekly research meetings attended by all the research staff which allows for communication about study issues and problems with any subjects in the study. In addition to the weekly site research meetings, the entire investigative team will meet by phone conference monthly, Face to face meetings will be held 2 times a year, to discuss the results of patients’ most recent study assessments as well as any adverse events. The research coordinators will inform the investigative team of any adverse medical events, or abnormal lab values, reported by study participants. All studies will be IRB approved and regular progress reports submitted to the IRB as well as adverse events (see below).

Protection against Study Procedure Risks

Phlebotomy **-** risks are reduced by the use of skilled nurses or study personnel who have had extensive experience with phlebotomy. The risks of an OGTT are minimized by utilizing trained nursing personnel with investigator back-up during the test. They are experienced in inserting IVs in study subjects. When we know of prior difficulties with these procedures, we will consult with Anesthesia to insert the IV and minimize pain and psychological trauma.

DXA - The whole body radiation dose to a female during a DXA is <1 mRem. The human race is continually being exposed to radiation from natural and man-made sources. The dose of background radiation to each person is 300-400 mRem so the exposure for these measurements is a minimal increase in exposure. For comparison purposes, this is less radiation exposure than from a routine chest x-ray and it is comparable to the radiation exposure from cosmic ray exposure during an airplane flight across the United States. The risk from this level of exposure is considered to be minimal. A negative pregnancy test will be obtained prior to these studies in all subjects. If an individual has participated in any other research study in the past 12 months that included exposure to ionizing radiation, we will assess overall exposure to radiation. If it exceeds 500 mrem for the calendar year by participating in this study, the potential participant will be excluded or moved to a later recruitment wave. Total ionizing radiation exposure will be evaluated with the help of the University of Pennsylvania and Penn State Radiation Protection Advisory Committees.

**8.2.1 Protection against Risk of Study Interventions**

Study subjects will be carefully screened for medical problems prior to randomization. The risks of the medications used in this study will be lowered through education and frequent follow-up. Study subjects will be monitored monthly in person or by telephone for adverse reactions. Only a limited supply of medication will be dispensed at each visit to encourage compliance with the protocol and prevent a prolonged exposure of an early gestation to medication.

Oral Contraceptive Pill -We will exclude subjects with all absolute contraindications to OCP including a history of thrombophlebitis, known or suspected clotting disorders, cerebrovascular or coronary artery disease or myocardial infarction, known or suspected uterine, cervical or breast neoplasia, history of a benign or malignant liver tumor that developed during the use of OCPs or other estrogen-containing products. Additionally, we will exclude other relative contraindications as listed in the exclusion criteria. Smoking will not be an absolute contraindication. Subjects will be counseled about the risks of OCP, and warning signs for serious adverse events including leg swelling, redness, pain, shortness of breath, racing heart, chest pain, severe and unremitting headache, unilateral weakness or speech disturbance. Subjects will have a scheduled visit or receive communication from the study team monthly and symptoms elicited and recorded as part of the visit. Worrisome signs will be reported to study physicians immediately for evaluation and management. We will have a low threshold of suspicion for discontinuing medication. We should note that we had no serious adverse events among 49 obese women randomized to a low dose OCP in the OWL-PCOS study (see table 2 for side effects above). Although the OWL-PCOS study showed a significant increase in prevalence of MetS in the OCP arm, only a small proportion of subjects developed DM or hypertension (4.6% and 6.7% respectively after 16 weeks). A fasting glucose level will be evaluated at the 16 week visit and a value >200mg/dl will prevent a subject from continuing in the study. BP will be measured at each visit and a subject with BP>150/100mmHg x2 will be unable to continue in the study.

Metformin - There is a small risk of lactic acidosis among women taking this medication. This most commonly occurs in patients with poorly controlled diabetes and impaired renal function. Women with these pre-existing medical problems will be excluded from the protocol as per the exclusion criteria. There have been reports of lactic acidosis induced by exposure to iodine-containing radiocontrast agents, such as those used for an intravenous pyelogram. Metformin XR will be stopped prior to procedures involving exposure to radiocontrast agents to reduce the chance of developing lactic acidosis (one week prior to medication and resume one week after the test). The development of lactic acidosis for any reason will be a reason for discontinuing participation in the study. Metformin XR is pregnancy category B with no known human teratogenic risk and no known embryonic lethality in humans. Metformin XR has been used throughout pregnancy in a number of studies with no adverse maternal or fetal effects. If a subject is pregnant during any part of the study, her participation will be stopped.

Preventing Pregnancy *-* All subjects will be asked to use barrier contraception during this study. Pregnancy tests will be performed at each in person study visit.

Menstrual irregularity - All subjects will be asked to keep detailed menstrual log and study coordinators and site PIs will review symptoms. Subjects may present with break through bleeding on the low dose OCP. Some subjects in the metformin arm may not have any menses during the study period. We will induce menses by administering medroxyprogesterone acetate at the screening visit if a subject has not had menses for 3 months. There is no data to suggest that lack of menses in the metformin arm for 6 months will increase risk of endometrial hyperplasia. In fact, several studies have described the biological plausibility of metformin having a protective effect on the endometrium. If a subject has not had menses during the study, we will administer medroxyprogesterone acetate after confirming a negative pregnancy test at the end of the study.

**Potential Benefits of the Proposed Research to Human Subjects and Others**

The subjects in this study will not benefit directly. The results from the study can be applied in the future to patients who stand to benefit from this information.

**Importance of the Knowledge to be Gained**

The importance of this study is high for the following reasons:

1. Both MetS and PCOS are major health problems and the risks of common treatments (OCP or metformin) on development of MetS are poorly understood.
2. Knowledge gained from this investigation will identify optimal strategies for treatment of overweight/obese women with PCOS
3. Understanding the underlying pathophysiology for alterations in metabolic risk with OCP or metformin use, will provide important information for future studies.

**Statistical Considerations**

**General Design Issues**

This will be a randomized, double-blind, double dummy clinical trial of three types of medication, OCP + placebo, metformin + placebo or OCP + metformin. We will track all subjects to completion. Subjects will be randomized 1:1:1 to the three treatments.

**Randomization**

Randomization is a critical feature of a clinical trial because it prevents treatment-selection biases. The study statistician will develop the programs for the randomization; however, the final random seeds used to generate the randomization scheme will be prepared by a statistician in PSU’s Department of Public Health Sciences independent of the study in order to keep the study statistician blinded as well. The randomization scheme for this study will use variable-size, random permuted blocks to ensure that the number of participants in each treatment arm is balanced after each set of *B* randomized participants, where *B* is the block size. Furthermore, the randomization will be stratified by recruitment site (UPenn/PSU), race (AA/non-AA), and the presence of MetS at baseline (yes/no). Randomization to the metformin or OCP arms will use 1:1:1 allocation.

**Outcomes**

**Primary Outcome Measurements**

The primary outcome for this trial is prevalence of MetS.

**Secondary Outcome Measurements**

We may assess change in HDL-C function, serum apolipoproteins, lipid particle size and number, body fat distribution, BMI, serum adipokines, HbA1c, glucose and insulin sensitivity, serum markers of inflammation, free fatty acids, androgens, quality of life parameters, cognitive testing and predictive factors for change in prevalence on MetS

**Sample Size and Accruals**

**Sample Size and Power Calculations**

The primary outcome of the COMET-PCOS trial will be to assess a linear trend in prevalence of MetS after 6 months’ treatment over the 3 arms assuming a 30% prevalence of MetS at baseline (derived from PENN data and OWL-PCOS). We anticipate a 15% subject drop-out over the course of the trial (in OWL-PCOS it was 8.1% over 16 weeks). Based on these assumptions, a sample size of 240 (80 per arm) will provide 80% statistical power to detect a linear trend in the prevalence of MetS over the 3 arms at the end of 6 months of 26% in the metformin arm, 40% in the OCP+metformin arm and 50% in the OCP arm using a two-sided test for linear trend with a significance level of 0.05.

**Table 10: Sample size scenarios based on varying assumptions**

| Proportion with MetS at the End of the Trial in Metformin Arm | Proportion with MetS at the End of the Trial in OCP+Metformin Arm | Proportion with MetS at the End of the Trial in OCP Arm | Total Sample Size (0% Drop-out) | Total Sample Size (15% Drop-out) | Power (%) | Type I Error (α) |
| --- | --- | --- | --- | --- | --- | --- |
| 0.25 | 0.38 | 0.50 | 204 | 240 | 84 | 0.05 |
| 0.25 | 0.38 | 0.52 | 204 | 240 | 89 | 0.05 |
| **0.26** | **0.40** | **0.50** | **204** | **240** | **80** | **0.05** |
| 0.26 | 0.40 | 0.52 | 204 | 240 | 86 | 0.05 |
| 0.28 | 0.42 | 0.52 | 204 | 240 | 80 | 0.05 |

**Statistical Analysis**

Primary analyses will invoke an intent-to-treat paradigm, wherein all randomized subjects are included according to their randomized treatment arm, regardless of actual treatment received, protocol violations, etc. Data will be summarized using descriptive statistics for continuous variables (mean, standard deviation, number of observations, and percentiles) and frequency statistics (frequencies and percentages) for categorical variables. The area under the curve (AUC) for glucose and insulin from the OGTT will be calculated using the trapezoidal rule. Univariate and bivariate distributions will be inspected in order to address any missing data, inconsistent responses, outliers, and data entry errors. The sample size estimates have taken into consideration a participant drop-out of 15%; however, every effort will be made during the studies to minimize any drop-out. If, however, study attrition appears to be an issue, we will use the observed data to determine if patients who completed the study differed from those who did not. To control for potential confounding factors for the association of the treatment effects with the metabolic syndrome, we have stratified the randomization by recruitment site, race, and the presence of metabolic syndrome at baseline. Although stratification may potentially yield unequal numbers between strata, within each individual stratum there will be approximately equal numbers and balance between treatment groups. All analyses will include these 3 randomization stratification factors as covariates in the statistical models. All hypothesis tests will be two-sided and all analyses will be performed using SAS software, version 9.4 (SAS Institute, Inc., Cary, NC), R software (open source), or Stata software, version 13 (StataCorp LP, College Station, TX).

For the primary outcome of the presence of the metabolic syndrome at the end of the 6-month trial, logistic regression will be used with independent variables that include terms for the treatment arm and the 3 randomization stratification factors as covariates, with a contrast constructed to test for linear trend over the three treatment arms.

A variety of secondary continuous outcomes will be collected during this longitudinal trial. These secondary outcomes include serum androgens, cholesterol efflux (HDL-C function) parameters, serum apolipoproteins, lipid particle size and number, anthropometric measures (BMI, adipokines, biomarkers of inflammation (e.g., hsCRP), measures of adipose tissue, abdominal adiposity, and quality of life measures (PCOSQ). For these continuous outcomes, linear mixed-effects models will be fit to assess differences between the treatment arms with respect to changes in these outcomes over time. The independent variables in the model will be treatment arm, time, the interaction of treatment and time, and the 3 randomization stratification factors as covariates. From the mixed-effects models, contrasts will be constructed to test the hypotheses of interest with respect to changes over time in the outcomes. Linear mixed-effects models are an extension of ordinary regression models that account for the between- and within-subject correlation inherent in longitudinal trials. Further, linear mixed-effects models do not drop patients with incomplete data and are easily extended to nonlinear mixed-effects models for ordinal data and count outcomes. Following our assessment of the initial fit of the models, we will add covariates to the models that correspond to other potential confounders (e.g., age) to assess their impact, if any, on the treatment effects. Residual diagnostics will be assessed to determine the appropriateness of the model fit and, if necessary, transformations of the response will be used to meet modeling assumptions. Differences in means and associated 95% confidence intervals (CIs) will be used to quantify the magnitude of the effects.

For any binary outcomes collected at each visit, analyses will be based on generalized estimating equations (GEE) with a logit link, an extension of logistic regression that accounts for correlated data within-subjects inherent in longitudinal trials, with independent variables that include terms for the treatment arm, time, the interaction of treatment and time, and the 3 randomization stratification factors as covariates. Following our assessment of the initial fit of the models, we will add covariates to the models that correspond to other potential confounders (e.g., age) to assess their impact, if any, on the treatment effects. The effect size will be quantified using the odds ratios (OR) and corresponding 95% CI.

**Accrual**

A total of 5 years will be required to complete the study after start up; 36-month enrollment period (based on 3 subjects per site/month x 2 sites), 1-month screening/randomization period, 6-month treatment period and time built in for data analysis and interpretation.

**Data Collection, Monitoring and Adverse Experience Reporting**

**Records to be kept**

Data will be collected prospectively by designated research personnel at each study site, supervised by the site PI. Original source documents will be kept in the study subject folder. Well-designed data collection forms will be developed to minimize data collection and recording errors. Administrative forms will be designed, such as visit procedure checklists, to assist the research staff in complying with protocol procedures. We will be collecting a medical history on each subject. We will also be obtaining biometric data, clinical data (hirsutism), imaging data from ultrasound, DXA scanning and biochemical data from blood. All data are being collected solely for the purpose of research and do not become part of the subject’s medical record.

All tissue samples and images will be labeled with the code number assigned to each subject and only de-identified samples will be sent to laboratories for testing. Specimens will be stored in Women’s Health Clinic Research Center’s freezers at UPenn and banked for batched analysis.

Study data will be managed using REDCap (Research Electronic Data Capture), a secure web application designed to support data capture for research studies, providing user-friendly web-based case report forms, real-time data entry validation (e.g., for data types and range checks), audit trails, a randomization module, and a de-identified data export mechanism to common statistical packages (SPSS, SAS, Stata, R/S-Plus). The database is hosted at the University of Pennsylvania, which will be used as a central location for data management. REDCap data collection projects rely on a thorough study-specific data dictionary defined in an iterative self-documenting process by all members of the research team. REDCap is flexible enough to be used for a variety of types of research and provides an intuitive user interface for database design and data entry.

Database access for REDCap is granted on a study-by-study basis. At the start of the trial when the database is created all research personnel will be given defined user roles and assigned a unique username and password. There is a 90-day password update policy for all REDCap users.  The REDCap database sits behind an application firewall and the data is stored on a virtual machine at Penn which is backed-up nightly.

**Maintenance/Retention of site records**

In order to comply with Good Clinical Practice (GCP) requirements, the investigators must maintain the master patient log that identifies all patients entered into the study for a period of two years after the study ends so that the subjects can be identified by audit. The PI must maintain adequate records pertaining to subjects’ files and other source data for a minimum of 5 years after completion of the study. Each clinical site will be responsible for ensuring study personal are trained and follow the data management guidelines of GCP and internal site policies.

**Data Security**

Study staff will make clear that subjects are not obligated to participate in the study and that their answers will be held strictly confidential. Questionnaires will not contain any identifying information, thus, ensuring the confidentiality of subjects’ responses. Code numbers will be assigned to each subject to maximize anonymity when entering and analyzing data. Any linking list of patient names and codes will be maintained separately, and destroyed at the earliest possible time. Only the investigators and research staff at each individual study center will have access to this information for patients recruited at that center. The second site and the research coordinator will not have access to the linking list. The security of the research project data will be maintained through network hardware and user authentication (usernames and passwords). Back-ups of the project data files will occur every night, with user data backed-up incrementally Monday through Thursday and complete back-ups every Friday. Archival back-ups, stored indefinitely, are cut on the last weekend of every month. All back-up data are stored in a secure off-site location. The number and variety of back-ups ensure ample data redundancy and protection. In addition, each participant will be assigned a unique subject identification number. Only the study coordinators will have the log linking this identification number with the participant’s personal information.

**Adverse Event Reporting**

**Serious Adverse Events**

All serious adverse events (SAEs) that occur from randomization through thirty days after the last dose of study medication must be reported. A serious adverse event is defined as: fatal or immediately life-threatening; severely or permanently disabling; requiring or prolonging inpatient hospitalization; overdose (intentional or accidental); ; or, any event adversely affecting the study’s risk/benefit ratio. Additionally, any event that, based on appropriate medical judgment, may jeopardize the subject’s health and may require medical or surgical intervention to prevent one of the outcomes listed above is considered an SAE.

If an SAE occurs and is thought to be related to the study medication, the study medication will be discontinued.

The site PI will report the SAE by completing and signing the Serious Adverse Event Report Form Subjects will be identified by subject identification number only. No other identifying information will be included on the form. The site PI must determine and record on the SAE form whether the SAE is unanticipated or anticipated, and if it is related, possibly related, or unrelated to participation in the research.

The DSMB and FDA (as applicable) will be notified and provide a determination regarding the SAE.

These determinations will dictate timeframes for sites’ submission to the DSMB (**Table 12**):

**Table 11: Types of Serious Adverse Events and their reporting requirements**

| **TYPE** | **SITE** |
| --- | --- |
| Unanticipated and related/possibly related SAE, fatal or life-threatening | Report to DSMB within 1 business day of discovery |
| Other unanticipated and related/possibly related SAE | Report to DSMB within 1 business day of discovery |
| Anticipated and related/possibly related SAE | Report to DSMB within 5 business days of discovery |
| Unrelated SAE (anticipated or unanticipated | Report to DSMB within 10 business days (no more than 3 weeks) of discovery |

Upon receiving notification of an SAE, the DSMB will review it via a closed-session email or conference-call discussion.

The PI will evaluate the frequency and severity of the SAEs and determine if modifications to the protocol and consent form are required. Site PIs will report the SAE to their site IRB according to local IRB requirements.

Adverse events deemed non-serious will also be recorded throughout study participation from the start of study drug through one week after the last dose of study medication. If an anticipated serious adverse event occurs at a frequency greater than expected, the DSMB will be notify the PI by the end of the next business day of discovery and follow the procedures for reporting serious and unanticipated and related adverse events. If an adverse event not initially determined to be reportable to the FDA under 21CFR312.32 is so reportable, the PI will report the adverse event to the FDA within 15 calendar days after the determination is made.

**Data Monitoring**

There will be no pre-determined stopping rules and no interim analysis.

**Study Monitoring**

A monitoring plan that satisfies the ICH/GCP guidelines for clinical monitoring will be used. The Penn designated monitor at the Women’s Health Clinical Research Center, will lead this effort, and report findings to the PI of both sites and the DSMB when necessary. The Penn designated monitor will have full knowledge of the study protocol, Manuals of Procedures, and is familiar with the database system redcap and is trained to review patient charts. The Penn designated monitor along with the Project Manager/Lead Study Coordinator at each site will be responsible for training and supervising other personnel.

Once personnel at participating site are trained to recruit patients, the Penn designated monitor will be sent to the site to help initiate the study according to the study protocol, and to ensure that the clinical site meets the scientific, clinical, and regulatory requirements. For example, the Penn designated monitor will review all signed and dated forms (such as financial disclosure forms), the curriculum vitae and certifications of the investigators and personnel, CRF training, and the written IRB approval of the protocol and consent form.

The Penn designated monitor will return to the clinical site after a defined number of patients are recruited (recruitment of the first 5 patients) and yearly thereafter.

During the site visit, the clinical sites should provide to the monitor(s) a space and access to all relevant records including medical records and regulatory binders, and there would be immediate verbal feedback provided to the site after original source documents are compared to entries in the CRF. The clinical sites must agree to cooperate with the monitor to ensure that any problems detected in the course of these monitoring visits are resolved. The on-site monitor will conduct an audit of a random sample of entered information against the source documents, a review of all regulatory documents, a review of all informed consents, and a review of all pharmacy logs. The clinical site PI and study coordinator should be available to meet the monitor during the visit. The monitor will review electronic data from all sites, providing a method for identifying systematic errors or problems.

To assure Good Clinical/Laboratory Practice, the monitor will control adherence to the protocol at the clinical sites and evaluate the competence of the personnel at the clinical sites including the ability to obtain written informed consents and record data correctly. The monitor will inform the PI, and DSMB regarding problems relating to facilities, technical equipment, or medical staff. A thorough written report will follow each site-visit and will include a detailed itemization of discrepancies and items requiring follow-up or reconciliation. The monitor will be responsible for maintaining regular contacts between the investigators in the clinical sites. When the study ends, the monitor will also visit the clinical site to provide assistance for close-out.

**Human Subjects Protection**

**Institutional Review Board (IRB) Review and Informed Consent**

This protocol and the informed consent document and any subsequent modifications will be reviewed and approved by the IRB responsible for oversight of the study. A signed consent form will be obtained from the subject. The consent form will describe the purpose of the study, the procedures to be followed, and the risks and benefits of participation. A copy of the consent form will be given to the subject, and this fact will be documented in the subject’s record.

**Subject Confidentiality**

All laboratory specimens, evaluation forms, reports, and other records that leave the site will be identified only by the Study Identification Number (SID) to maintain subject confidentiality. All records will be kept in a locked file cabinet. All computer entry and networking programs will be done using SIDs only to prevent the loss of confidentiality. Clinical information will not be released without written permission of the subject, except as necessary for monitoring by IRB, the OHRP, the sponsor, or the sponsor’s designee.

In the event that a subject revokes authorization to collect or use PHI, the investigator, by regulation, retains the ability to use all information collected prior to the revocation of subject authorization. For subjects that have revoked authorization to collect or use PHI, attempts should be made to obtain permission to collect at least vital status (i.e. that the subject is alive) at the end of their scheduled study period

**Study Modification/Discontinuation**

The study may be modified or discontinued at any time by the IRB, the NIH, the OHRP, or other government agencies as part of their duties to ensure that research subjects are protected.

**Data and Safety Monitoring Board**

Kathy Hoeger from the University of Rochester Medical Center will chair the DSMB for this clinical trial. The DSMB will review safety information, especially SAEs that may occur, on a quarterly basis while the study is active and ongoing. Kathy will be consulted for any safety questions that may arise during the trail.

**References**

1 Shroff R, Syrop CH, Davis W, Van Voorhis BJ, **Dokras** A. Risk of metabolic complications in the new PCOS phenotypes based on the Rotterdam criteria. Fertil Steril. 2007 Nov;88(5):1389-95.

2 Wild RA, Carmina E, Diamanti-Kandarakis E, **Dokras** A, Escobar-Morreale HF, Futterweit W, Lobo R, Norman RJ, Talbott E, Dumesic DA. Assessment of cardiovascular risk and prevention of cardiovascular disease in women with the polycystic ovary syndrome: a consensus statement by the Androgen Excess and Polycystic Ovary Syndrome (AE-PCOS) Society. J Clin Endocrinol Metab. 2010 May;95(5):2038-49.

3Panidis D, Tziomalos K, Misichronis G, Papadakis E, Betsas G, Katsikis I, Macut D. Insulin resistance and endocrine characteristics of the different phenotypes of polycystic ovary syndrome: a prospective study. Hum Reprod 2012; 27:541-549

4 Després JP, Couillard C, Gagnon J, Bergeron J, Leon AS, Rao DC, Skinner JS, Wilmore JH, Bouchard C. Race, visceral adipose tissue, plasma lipids, and lipoprotein lipase activity in men and women: the Health, Risk Factors, Exercise Training, and Genetics (HERITAGE) family study. Arterioscler Thromb Vasc Biol. 2000 Aug;20(8):1932-8.

114 Katzmarzyk PT, Bray GA, Greenway FL, Johnson WD, Newton RL Jr, Ravussin E, Ryan DH, Smith SR, Bouchard C.Racial differences in abdominal depot-specific adiposity in white and African American adults. Am J Clin Nutr. 2010 Jan;91(1):7-15.

5 Hillman JK, Johnson LN, Limaye M, Feldman RA, Sammel M, **Dokras** A Black women with polycystic ovary syndrome (PCOS) have increased risk for metabolic syndrome and cardiovascular disease compared with white women with PCOS [corrected].Fertil Steril. 2014 Feb;101(2):530-5

6 Hillman JK, Johnson LN, Limaye M, Feldman RA, Sammel M, **Dokras** A Black women with polycystic ovary syndrome (PCOS) have increased risk for metabolic syndrome and cardiovascular disease compared with white women with PCOS [corrected].Fertil Steril. 2014 Feb;101(2):530-5

7 **Legro** RS, Dodson WC, Kris-Etherton PM, Kunselman AR, Stetter CM, Williams NI, Gnatuk CL, Estes SJ, Fleming J, Allison KC, Sarwer DB, Coutifaris C, **Dokras A**. Randomized Controlled Trial of Preconception Interventions in Infertile Women With Polycystic Ovary Syndrome. J Clin Endocrinol Metab. 2015 Nov;100(11):4048-58

8 Hollinrake E, Abreu A, Maifeld M, Van Voorhis BJ, **Dokras** A.Increased risk of depressive disorders in women with polycystic ovary syndrome. Fertil Steril. 2007 Jun;87(6):1369-76.

9Kerchner A, Lester W, Stuart SP, **Dokras** A. Risk of depression and other mental health disorders in women with polycystic ovary syndrome: a longitudinal study.Fertil Steril. 2009 Jan;91(1):207-12

10 Rohatgi A, Khera A, Berry JD, Givens EG, Ayers CR, Wedin KE, Neeland IJ, Yuhanna IS, Rader DR, de Lemos JA, Shaul PW. HDL cholesterol efflux capacity and incident cardiovascular events. N Engl J Med. 2014 Dec 18;371(25):2383-93.

11Khera AV, Cuchel M, laLleraMoya M, Rodrigues A, Burke MF, Jafri K,French BC, Phillips JA, Mucksavage M L, Wilensky RL, Mohler ER, Rothblat GH,Rader DJ Cholesterol efflux capacity, high-density lipoprotein function, and atherosclerosis. N Engl J Med. 2011;364:127–135.

12 Roe A, Hillman J, Butts S, Smith M, Rader D, Playford M, **Mehta NN**, **Dokras A**. Decreased cholesterol efflux capacity and atherogenic lipid profile in young women with PCOS.J Clin Endocrinol Metab. 2014 May;99(5):E841-7

13 **Legro** RS, Barnhart HX, Schlaff WD, Carr BR, Diamond MP, Carson SA, Steinkampf MP, **Coutifaris C,** McGovern PG, Cataldo NA, Gosman GG, Nestler JE, Giudice LC, Leppert PC, Myers ER; Clomiphene, metformin, or both for infertility in the polycystic ovary syndrome. N Engl J Med 2007;356(6):551-66

14Sniderman AD, Scantlebury T, Cianflone K. Hypertriglyceridemic hyperapob: the unappreciated atherogenic dyslipoproteinemia in type 2 diabetes mellitus. Ann Intern Med. 2001; 135: 447–459

15 Lewis GF, Uffelman KD, Szeto LW, Weller B, Steiner G. Interaction between free fatty acids and insulin in the acute control of very low density lipoprotein production in humans. J Clin Invest*.* 1995*;* 95*:* 158*–166.*

16 Hotamisligil GS, Shargill NS, Spiegelman BM. Adipose expression of tumor necrosis factor-alpha: direct role in obesity-linked insulin resistance. Science 1993;259:87–91.

17 Baumann H, Gauldie J. Regulation of hepatic acute phase plasma protein genes by hepatocyte stimulating

factors and other mediators of inflammation.Mol Biol Med 1990;7:147–59.

18 Trevisan M, Liu J, Bahsas FB, Menotti A. Syndrome X and mortality: a population-based study. Risk Factor and Life Expectancy Research Group. Am J Epidemiol. 1998;148(10):958-66.

19 Wannamethee SG, Shaper AG, Lennon L, Morris RW. Metabolic syndrome vs Framingham Risk Score for prediction of coronary heart disease, stroke, and type 2 diabetes mellitus. Archives of Internal Medicine. 2005;165:2644–2650.

20 Mottillo S, Filion KB, Genest J, et al. The metabolic syndrome and cardiovascular risk: a systematic review and meta-analysis. J Am Coll Cardiol 2010;56:1113–32.

21 Huxley R, Barzi F, Woodward M. Excess risk of fatal coronary heart disease associated with diabetes in men and women: meta-analysis of 37 prospective cohort studies. BMJ 2006;332:73–8.

22 Devers MC, Campbell S, Simmons D. Influence of age on the prevalence and components of the metabolic syndrome and the association with cardiovascular disease. BMJ Open Diabetes Res Care. 2016 Apr 25;4(1)

23 **Dokras A**, Bochner M, Hollinrake E, Markham S, Vanvoorhis B, Jagasia DH. Screening women with polycystic ovary syndrome for metabolic syndrome.Obstet Gynecol. 2005 Jul;106(1):131-7.

24Moran LJ, Misso ML, Wild RA, Norman RJ. Impaired glucose tolerance, type 2 diabetes and metabolic syndrome in polycystic ovary syndrome: a systematic review and meta-analysis. Hum Reprod Update.2010; 16(4):347-63.

25 Escobar-Morreale HF, Luque-Ramírez M, González F.Circulating inflammatory markers in polycystic ovary syndrome: a systematic review and metaanalysis.Fertil Steril. 2011 Mar 1;95(3):1048-58.

26Toulis KA, Goulis DG, Mintziori G, Kintiraki E, Eukarpidis E, Mouratoglou SA, Pavlaki A, Stergianos S, Poulasouchidou M, Tzellos TG, Makedos A, Chourdakis M, Tarlatzis BC.Meta-analysis of cardiovascular disease risk markers in women with polycystic ovary syndrome.Hum Reprod Update. 2011 Nov-Dec;17(6):741- 60

27 **Dokras A**.Cardiovascular disease risk in women with PCOS.Steroids. 2013 Apr 26.

28 Solomon CG, Hu FB, Dunaif A, Rich-Edwards JE, Stampfer MJ, Willett WC, Speizer FE, Manson JE. Menstrual cycle irregularity and risk for future cardiovascular disease. J Clin Endocrinol Metab 2002;87(5):2013-2017.

29Mani H, Levy MJ, Davies MJ, Morris DH, Gray LJ, Bankart J, Blackledge H, Khunti K, Howlett TA. Diabetes and cardiovascular events in women with polycystic ovary syndrome: a 20-year retrospective cohort study. Clin Endocrinol (Oxf). 2013 Jun;78(6):926-34.

30 Azziz R, Marin C, Hoq L, Badamgarav E, Song P. Health care-related economic burden of the polycystic ovary syndrome during the reproductive life span. J Clin Endocrinol Metab 2005;90(8):4650-8.

31**Dokras A**, Clifton S, Futterweit W, Wild R Increased risk for abnormal depression scores in women with polycystic ovary syndrome: a systematic review and meta-analysis..Obstet Gynecol. 2011 Jan;117(1):145-52.

32 **Legro** RS, Arslanian SA, Ehrmann DA, Hoeger KM, Murad MH, Pasquali R, Welt CK; Endocrine Society. Diagnosis and treatment of polycystic ovary syndrome: an Endocrine Society clinical practice guideline. J Clin Endocrinol Metab. 2013 Dec;98(12):4565-92

33 ACOG Practice Bulletin No. 108: Polycystic ovary syndrome. Obstet Gynecol. 2009 Oct;114(4):936-49. ACOG Committee on Practice Bulletins--Gynecology.

34 Rotterdam ESHRE/ASRM-sponsored PCOS consensus workshop group. Revised 2003 consensus on diagnostic criteria and long-term health risks related to polycystic ovary syndrome (PCOS). Hum Reprod 2004;1:41–7.

35 Costello M, Shrestha B, Eden J, Sjoblom P, Johnson N. Insulin-sensitising drugs versus the combined oral contraceptive pill for hirsutism, acne and risk of diabetes, cardiovascular disease, and endometrial cancer in polycystic ovary syndrome. Cochrane Database Syst Rev. 2007 Jan 24;(1):CD005552. Review.

36 Archer JS, Chang RJ. Hirsutism and acne in polycystic ovary syndrome. Best Pract Res Clin Obstet Gynaecol 2004;18(5):737-54.

37 Corbould A Effects of androgens on insulin action in women: is androgen excess a component of female metabolic syndrome? Diabetes Metab Res Rev. 2008 Oct;24(7):520-32.

38Rimm EB, Manson JE, Stampfer MJ, Colditz GA, Willett WC, Rosner B, Hennekens CH and Speizer FE (1992) Oral contraception use and the risk of type 2 diabetes in a large prospective study of women. Diabetologia 35,967–972.

39 Watanabe RM, Azen CG, Roy S, Perlman JA, Bergman RN. Defects in carbohydrate metabolism in oral contraceptive users without apparent metabolic risk factors. J Clin EndocrinolMetab 1994;79:1277–1283

40 Chasen-Taber L, Willett WC, Stampfer MJ, Hunter DJ, Colditz GA, Spiegelman Dand Manson JE. A prospective study of oral contraceptives andNIDDM among U.S. women. Diabetes Care 1997; 20,330–335.

41 van Rooijen M, Hansson LO, Frostegård J, Silveira A, Hamsten A, Bremme K Treatment with combined oral contraceptives induces a rise in serum C-reactive protein in the absence of a general inflammatory response. J Thromb Haemost. 2006 Jan;4(1):77-82.

42 Krintus M, Sypniewska G, Kuligowska-Prusinska M. Effect of second and third generation oral contraceptives on C-reactive protein, lipids and apolipoproteins in young, non-obese, non-smoking apparently healthy women. Clin Biochem. 2010 Apr;43(6):626-8.

43 Ball MJ, Ashwell E, Jackson M, Gillmer MD Comparison of two triphasic contraceptives with different progestogens: effects on metabolism and coagulation proteins..Contraception. 1990 Apr;41(4):363-76.

44 Van Rooijen M, Schoultz BV, Silveira A, Hamsten A and Bremme K (2002) Different effects of oral contraceptives containing levonorgestrel or desogestrel on plasma lipoproteins and coagulation factor VII. Am J Obstet Gynecol 186,44–48.

45 Halperin IJ, Kumar SS, Stroup DF, Laredo SE The association between the combined oral contraceptive pill and insulin resistance, dysglycemia and dyslipidemia in women with polycystic ovary syndrome: a systematic

review and meta-analysis of observational studies..Hum Reprod. 2011 Jan;26(1):191-201

46Dunaif A, Segal KR, Futterweit W, Dobrjansky A 1989 Profound peripheral insulin resistance, independent of obesity, in polycystic ovary syndrome. Diabetes 38:1165-1174

47 Ciaraldi TP, el-Roeiy A, Madar Z, Reichart D, Olefsky JM, Yen SS 1992. Cellular mechanisms of insulin resistance in polycystic ovarian syndrome. J Clin Endocrinol Metab 75(2):577-83.

48 Dunaif A, Wu X, Lee A, Diamanti-Kandarakis E 2001. Defects in insulin receptor signaling in vivo in the polycystic ovary syndrome (PCOS). Am J Physiol Endocrinol Metab 281(2):E392-9.

49Ciaraldi TP, Aroda V, Mudaliar S, Chang RJ, Henry RR 2009. Polycystic ovary syndrome is associated with tissue-specific differences in insulin resistance. J Clin Endocrinol Metab 94(1):157-63.

50 Rosenbaum D, Haber RS, Dunaif A 1993. Insulin resistance in polycystic ovary syndrome: decreased expression of GLUT-4 glucose transporters in adipocytes. Am J Physiol 264(2 Pt 1):E197-202.

51Dunaif A. Drug insight: insulin-sensitizing drugs in the treatment of polycystic ovary syndrome--a reappraisal Nat Clin Pract Endocrinol Metab. 2008 May;4(5):272-83.

52 Bailey CJ, Turner RC. Metformin. N Engl J Med. 1996;334(9):574–9.

53 Knowler WC, Barrett-Connor E, Fowler SE, Hamman RF, Lachin JM, Walker EA, Nathan DM; Reduction inm the incidence of type 2 diabetes with lifestyle intervention or metformin. Diabetes Prevention Program Research Group. N Engl J Med. 2002 Feb 7;346(6):393-403.

54 Effect of intensive blood-glucose control with metformin on complications in overweight patients with type 2 diabetes (UKPDS 34). UK Prospective Diabetes Study (UKPDS) Group. [No authors listed]Lancet. 1998 Sep 12;352(9131):854-65.

55Quinn SM, Baur LA, Garnett SP, Cowell CT. Treatment of clinical insulin resistance in children: a systematic review. Obes Rev. 2010;11(10):722–30.

56Kendall D, Vail A, Amin R, Barrett T, Dimitri P, Ivison F, et al. Metformin in obese children and adolescents: the MOCA Trial. J Clin Endocrinol Metab. 2013;98(1):322–9. A randomized trial which assessed the effect of metformin on body weight, metabolic risk factors, and adipokines in obese children and adolescents with hyperinsulinemia and/or impaired fasting glucose or impaired glucose tolerance.

57 Tang T, Lord JM, Norman RJ, Yasmin E, Balen AH.Insulin-sensitising drugs (metformin, rosiglitazone, pioglitazone, D-chiro-inositol) for women with polycystic ovary syndrome, oligo amenorrhoea and subfertility. Cochrane Database Syst Rev. 2012 May 16;5:CD003053. doi: 10.1002/14651858.CD003053.pub5. Review.

58 J.M. Lord, I.H. Flight, R.J. Norman, Metformin in polycystic ovary syndrome: systematic review and meta- analysis. Br. Med. J. 327, 951–953 (2003)

59 Nieuwenhuis-Ruifrok AE1, Kuchenbecker WK, Hoek A, Middleton P, Norman RJ. Insulin sensitizing drugs for weight loss in women of reproductive age who are overweight or obese: systematic review and meta-analysis. Hum Reprod Update. 2009 Jan-Feb;15(1):57-68

60 Salpeter SR, Buckley NS, Kahn JA, Salpeter EE. Meta-analysis: metformin treatment in persons at risk for diabetes mellitus..Am J Med. 2008 Feb;121(2):149-157

61 Morin-Papunen L, Rautio K, Ruokonen A, Hedberg P, Puukka M, Tapanainen JS Metformin reduces serum C-reactive protein levels in women with polycystic ovary syndrome. J Clin Endocrinol Metab. 2003 Oct;88(10):4649-54.

62 Thethi TK, Katalenich B, Nagireddy P, Chabbra P, Md NK, Fonseca V.Role of insulin sensitizers on cardiovascular risk factors in polycystic ovarian syndrome – a meta-analysis.Endocr Pract. 201 Jun;21(6):645-67

63 Naderpoor N, Shorakae S, de Courten B, Misso ML, Moran LJ, Teede HJ.Metformin and lifestyle modification in polycystic ovary syndrome: systematic review and meta-analysis. Hum Reprod Update. 2015 Sep-Oct;21(5):560-74.

64 Costello M, Shrestha B, Eden J, Sjoblom P, Johnson N Insulin-sensitising drugs versus the combined oral contraceptive pill for hirsutism, acne and risk of diabetes, cardiovascular disease, and endometrial cancer in polycystic ovary syndrome..Cochrane Database Syst Rev. 2007

65 Morin-Papunen LC, Vauhkonen I, Koivunen RM, Ruokonen A, Martikainen HK, Tapanainen JS. Endocrine and metabolic effects of metformin versus ethinyl estradiol-cyproterone acetate in obese women with polycystic ovary syndrome: a randomized study. J Clin Endocrinol Metab 2000 Sep;85(9):3161-8.

66 Rautio K, Tapanainen JS, Ruokonen A, Morin-Papunen LC. Effects of metformin and ethinyl estradiol- cyproterone acetate on lipid levels in obese and non-obese women with polycystic ovary syndrome. Eur J Endocrinol. 2005 Feb;152(2):269-75.

67 Harborne L, Fleming R, Lyall H, Sattar N, Norman J Metformin or antiandrogen in the treatment of hirsutism in polycystic ovary syndrome. J Clin Endocrinol Metab. 2003 Sep;88(9):4116-23.

68 Luque-Ramírez M, Alvarez-Blasco F, Escobar-Morreale HF. Antiandrogenic contraceptives increase serum adiponectin in obese polycystic ovary syndrome patients. Obesity (Silver Spring). 2009 Jan;17(1):3-9.

69 Moran LJ, Meyer C, Hutchison SK, Zoungas S, Teede HJ.Novel inflammatory markers in overweight women with and without polycystic ovary syndrome and following pharmacological intervention. J Endocrinol Invest. 2010 Apr;33(4):258-65.

70Glintborg D, Mumm H, Altinok ML, Richelsen B, Bruun JM, Andersen M. Adiponectin, interleukin-6, monocyte chemoattractant protein-1, and regional fat mass during 12-month randomized treatment with metformin and/or oral contraceptives in polycystic ovary syndrome.J Endocrinol Invest. 2014 Aug;37(8):757-64.

71 Glintborg D, Altinok ML, Mumm H, Hermann AP, Ravn P, Andersen M.Body composition is improved during 12 months' treatment with metformin alone or combined with oral contraceptives compared with treatment with oral contraceptives in polycystic ovary syndrome. J Clin Endocrinol Metab. 2014 Jul;99(7):2584-91

72Cussons AJ, Stuckey BG, Walsh JP, Burke V & Norman RJ Polycystic ovarian syndrome: marked differences between endocrinologists and gynaecologists in diagnosis and management. Clinical Endocrinology 2005 62 289–295.

73 Conway G, Dewailly D, Diamanti-Kandarakis E, Escobar-Morreale HF, Franks S, Gambineri A, Kelestimur F, Macut D, Micic D, Pasquali R, Pfeifer M, Pignatelli D, Pugeat M, Yildiz B; ESE PCOS Special Interest Group.European survey of diagnosis and management of the polycystic ovary syndrome: results of the ESE PCOS Special Interest Group's Questionnaire. Eur J Endocrinol. 2014 Oct;171(4):489-98.

74Bonny AE, Appelbaum H, Connor EL, Cromer B, DiVasta A, Gomez-Lobo V, Harel Z, Huppert J, Sucato G; NASPAG Research Committee. Clinical variability in approaches to polycystic ovary syndrome. J Pediatr Adolesc Gynecol. 2012 Aug;25(4):259-61.

75Misso M, Boyle J, Norman R, Teede H.Development of evidenced-based guidelines for PCOS and implications for community health.Semin Reprod Med. 2014 May;32(3):230-40.

76 Domecq JP, Prutsky G, Mullan RJ, Hazem A, Sundaresh V, Elamin MB, Phung OJ, Wang A, Hoeger K, Pasquali R, Erwin P, Bodde A, Montori VM, Murad MH Lifestyle modification programs in polycystic ovary syndrome: systematic review and meta-analysis.

77 Eckel RH, Alberti KG, Grundy SM, Zimmet PZ. The metabolic syndrome. Lancet. 2010;375:181–3.

78 Walsh BW, Schiff I, Rosner B, Greenberg L, Ravnikar V, Sacks FM. Effects of postmenopausal estrogen replacement on the concentrations and metabolism of plasma lipoproteins. N Engl J Med. 1991;325:1196- 1204.

79 Tikkanen MJ, Nikkila EA, Kussi T, Sipinens S. High density lipoprotein-2 and hepatic lipase: reciprocal changes produced by estrogen and norgestrel. J Clin Endocrinol Metab. 1982;54:1113-1117.

80Barter PJ, Caulfield M, Eriksson M, Grundy SM, Kastelein JJ, Komajda M, Lopez-Sendon J, Mosca L, Tardif JC, Waters DD, Shear CL, Revkin JH, Buhr KA, Fisher MR, Tall AR, Brewer B. Effects of torcetrapib in patients at high risk for coronary events. N Engl J Med. 2007;357:2109–2122

81 Khera AV, Patel PJ, Reilly MP, Rader DJ.The addition of niacin to statin therapy improves high-density lipoprotein cholesterol levels but not metrics of functionality. J Am Coll Cardiol. 2013 Nov 12;62(20):1909-10.

82 Badeau RM, Metso J, Kovanen PT, Lee-Rueckert M, Tikkanen MJ, Jauhiainen M. The impact of gender and serum estradiol levels on HDL-mediated reverse cholesterol transport. Eur J Clin Invest. 2013 Apr;43(4):317- 23

83 McGillicuddy FC, de la Llera Moya M, Hinkle CC, Joshi MR, Chiquoine EH, Billheimer JT, Rothblat GH, Reilly MInflammation impairs reverse cholesterol transport in vivo.Circulation. 2009 Mar 3;119(8):1135-45.

84Matsuki K, Tamasawa N, Yamashita M, Tanabe J, Murakami H, Matsui J, Imaizumi T, Satoh K, Suda T Metformin restores impaired HDL-mediated cholesterol efflux due to glycation. Atherosclerosis. 2009 Oct;206(2):434-8.

85Sarwar N, Danesh J, Eiriksdottir G, et Triglycerides and the risk of coronary heart disease: 10,158 incident cases among 262,525 participants in 29 Western prospective studies. Circulation. 2007;115(4):450–45

86 Jørgensen AB, Frikke-Schmidt R, Nordestgaard BG, Tybjærg-Hansen A Loss-of-function mutations in APOC3 and risk of ischemic vascular disease.. N Engl J Med. 2014 Jul 3;371(1):32-41

87 Watson TD, Caslake MJ, Freeman DJ, Griffin BA, Hinnie J, Packard CJ, Shepherd J. Determinants of LDL subfraction distribution and concentrations in young normolipidemic subjects. Arterioscler Thromb. 1994; 14:902–9

88 Berneis KK, Krauss RM. Metabolic origins and clinical significance of LDL heterogeneity. J Lipid Res. 2002; 43: 1363–1379.

89 Mora S, Otvos JD, Rosenson RS, Pradhan A, Buring JE, Ridker PM. Lipoprotein particle size and concentration by nuclear magnetic resonance and incident type 2 diabetes in women. Diabetes. 2010 May;59(5):1153-60

90 Goff DC Jr, D’Agostino RB Jr, Haffner SM, Otvos JD. Insulin resistance and adiposity influence lipoprotein size and subclass concentrations: results from the Insulin Resistance Atherosclerosis Study. Metabolism. 2005; 54: 264–270.

91 Garvey WT, Kwon S, Zheng D, Shaughnessy S, Wallace P, Hutto A, Pugh K, Jenkins AJ, Klein RL, Liao Y. Effects of insulin resistance and type 2 diabetes on lipoprotein subclass particle size and concentration determined by nuclear magnetic resonance. Diabetes. 2003; 52: 453–462.

92 Mora S, Rifai N, Buring JE, Ridker PM. Comparison of LDL cholesterol concentrations by Friedewald calculation and direct measurement in relation to cardiovascular events in 27,331 women. Clin Chem. 2009 May;55(5):888-94.

93 Goldberg R, Temprosa M, Otvos J, Brunzell J, Marcovina S, Mather K, Arakaki R, Watson K, Horton E, Barrett-Connor E Lifestyle and metformin treatment favorably influence lipoprotein subfraction distribution in the Diabetes Prevention Program. J Clin Endocrinol Metab. 2013 Oct;98(10):3989-98.

94 InterAct Consortium, Langenberg C, Sharp SJ, Schulze MB, Rolandsson O, Overvad K, Forouhi NG, Spranger J, Drogan D, Huerta JM, Arriola L, de Lauzon-Guillan B, Tormo MJ, Ardanaz E, Balkau B, Beulens JW, Boeing H, Bueno-de-Mesquita HB, Clavel-Chapelon F, Crowe FL, Franks PW, Gonzalez CA, Grioni S, Halkjaer J, Hallmans G, Kaaks R, Kerrison ND, Key TJ, Khaw KT, Mattiello A, Nilsson P, Norat T, Palla L, Palli D, Panico S, Quirós JR, Romaguera D, Romieu I, Sacerdote C, Sánchez MJ, Slimani N, Sluijs I, Spijkerman AM, Teucher B, Tjonneland A, Tumino R, van der A DL, van der Schouw YT, Feskens EJ, Riboli E, Wareham NJ. Long-term risk of incident type 2 diabetes and measures of overall and regional obesity: the EPIC-InterAct case-cohort study. PLoS Med. 2012;9(6):e1001230.

95 Emerging Risk Factors Collaboration, Wormser D, Kaptoge S, Di Angelantonio E, Wood AM, Pennells L, Thompson A, Sarwar N, Kizer JR, Lawlor DA, Nordestgaard BG, Ridker P, Salomaa V, Stevens J, Woodward M, Sattar N, Collins R, Thompson SG, Whitlock G, Danesh J.Separate and combined associations of body-mass index and abdominal adiposity with cardiovascular disease: collaborative analysis of 58 prospective studies. Lancet. 2011 Mar 26;377(9771):1085-95

96 Lim SS, Norman RJ, Davies MJ, Moran LJ. The effect of obesity on polycystic ovary syndrome: a systematic review and meta-analysis. Obes Rev. 2013 Feb;14(2):95-109.

97 Faloia E, Canibus P, Gatti C, Frezza F, Santangelo M, Garrappa GG, Boscaro M2004 Body composition, fat

distribution and metabolic characteristics in lean and obese

98 Barber, T.M., Golding, S.J., Alvey, C., Wass, J.A., Karpe, F., Franks, S., McCarthy, M.I., Global adiposity rather than abnormal regional fat distributioncharacterises women with polycystic ovary syndrome. 2007. J.Clin. Endocrinol.Metab. 93, 999–1004.

99 Mannerås-Holm L, Leonhardt H, Kullberg J, et al. Adipose tissuehas aberrant morphology and function in PCOS: enlarged adipocytes and low serum adiponectin, but not circulating sex steroids, are strongly associated with insulin resistance. J Clin Endocrinol Metab. 2011;96:304–311.

100 Carmina E, Bucchieri S, Esposito A, Del Puente A, Mansueto P, Orio F, Di Fede G, Rini G. Abdominal fat quantity and distribution in women with polycystic ovary syndrome and extent of its relation to insulin resistance. J Clin Endocrinol Metab. 2007 Jul;92(7):2500-5.

101 Svendsen PF, Nilas L, Nørgaard K, Jensen JE, Madsbad S. Obesitybody composition and metabolic disturbances in polycystic ovarysyndrome. Hum Reprod. 2008;23:2113–2121.

102 Puder, J.J., Varga, S., Kraenzlin, M., De Geyter,C.,Keller, U., Muller, B., 2005. Central fat excess in polycystic ovary syndrome: relation to low-grade inflammation and insulin resistance. J. Clin. Endocrinol. Metab. 90, 6014–6021.

103van de Woestijne AP, Monajemi H, Kalkhoven E, Visseren FL. Adipose tissue dysfunction and hypertriglyceridemia: mechanisms and management. Obes Rev. 2011 Oct;12(10):829-40.

104 Ibáñez L, de Zegher F. Ethinylestradiol-drospirenone, flutamide-metformin, or both for adolescents anwomen with hyperinsulinemic hyperandrogenism: opposite effects on adipocytokines and body adiposity. J Clin Endocrinol Metab. 2004 Apr;89(4):1592-7.

105Aydin K, Cinar N, Aksoy DY, Bozdag G, Yildiz BO.Body composition in lean women with polycystic ovary syndrome: effect of ethinyl estradiol and drospirenone combination. Contraception. 2013 Mar;87(3):358-62.

106 Ibáñez L, Díaz M, Sebastiani G, Marcos MV, López-Bermejo A, de Zegher F.Oral contraception vs insulin sensitization for 18 months in nonobese adolescents with androgen excess: posttreatment differences in C- reactive protein, intima-media thickness, visceral adiposity, insulin sensitivity, and menstrual regularity. J Clin Endocrinol Metab. 2013 May;98(5)

107Diamanti-Kandarakis E, Dunaif A Insulin resistance and the polycystic ovary syndrome revisited: an update on mechanisms and implications. Endocr Rev. 2012 Dec;33(6):981-1030.

108 Ibáñez L, Lopez-Bermejo A, Diaz M, Marcos MV, de Zegher F. Pubertal metformin therapy to reduce total, visceral, and hepatic adiposity. J Pediatr. 2010 Jan;156(1):98-102

109Lord J, Thomas R, Fox B, Acharya U, Wilkin T. The effect of metformin on fat distribution and the metabolic syndrome in women with polycystic ovary syndrome--a randomised, double-blind, placebo-controlled trial. BJOG. 2006 Jul;113(7):817-24.

110 R. Pasquali, A. Gambineri, D. Biscotti, V. Vicennati, L. Gagliardi, D. Colitta, S. Fiorini, G.E. Cognigni, M. Filicori, A.M. Morselli-Labate, Effect of long-term treatment with metformin added to hypocaloric diet on body composition, fat distribution, androgen and insulin levels, in abdominally obese women with and without the polycystic ovary syndrome. J. Clin. Endocrinol. Metab. 85, 2767–2774 (2000)

111 Maffei, M., Halaas, J., Ravussin, E., Pratley, R.E., Lee, G.H., Zhang, Y., Fei, H., Kim, S., Lallone, R., Ranganathan, S., 1995. Leptin levels in human and rodent: measurement of plasma leptin and ob RNA in obese and weight-reduced subjects. Nat. Med. 1, 1155–1161.

112 Barber TM, Franks S Adipocyte biology in polycystic ovary syndrome..Mol Cell Endocrinol. 2013 Jul 5;373(1-2):68-76.

113 Lara-Castro C, Luo N, Wallace P, Klein RL, Garvey WT Adiponectin multimeric complexes and the metabolic syndrome trait cluster..Diabetes. 2006 Jan;55(1):249-59.

114 Toulis, K.A., Goulis, D.G., Farmakiotis, D., Georgopoulos, N.A., Katsikis, I., Tarlatzis, B.C., Papadimas, I., Panidis, D., 2009. Adiponectin levels in women with polycystic ovary syndrome: a systematic review and a meta-analysis. Hum. Reprod. Update 15, 297–307.

115 Tarkun I, Dikmen E, Cetinarslan B, Cantürk Z.Impact of treatment with metformin on adipokines in patients with polycystic ovary syndrome.Eur Cytokine Netw. 2010 Dec;21(4):272-7.

116 Singh S, Akhtar N, Ahmad J. Plasma adiponectin levels in women with polycystic ovary syndrome: impact of metformin treatment in a case-control study. Diabetes Metab Syndr. 2012 Oct-Dec;6(4):207-11.

117 Esfahanian F, Zamani MM, Heshmat R, Moini nia F. Effect of metformin compared with hypocaloric diet on serum C-reactive protein level and insulin resistance in obese and overweight women with polycystic ovary syndrome. J Obstet Gynaecol Res. 2013 Apr;39(4):806-13.

118 Luque-Ramírez M, Escobar-Morreale HF. Treatment of polycystic ovary syndrome (PCOS) with metformin ameliorates insulin resistance in parallel with the decrease of serum interleukin-6 concentrations. Horm Metab Res. 2010 Oct;42(11):815-2

**Protocol changes during trial with IRB dates of approval**

| Version date | Version | IRB approval date | Change |
| --- | --- | --- | --- |
| 7/25/2017 | 1 | 9/5/2017 | No Change original protocol |
| 10/19/2017 | 2 | 11/17/2017 | "Table of contents updated accordingly  Page 9: Hypothesis: updated to match what was listed in the grant application. “OCPs increase the risk of MetS specifically by producing an atherogenic lipoprotein phenotype, increasing blood pressure and/ or body weight while metformin modestly decreases MetS risk by decreasing body weight and improving lipid phenotype.”  Treatment: Provera changed from 4 weeks to 3 months prior to screening visit. This was decided by the physicians as they believe it is in the patients be interest and this larger window remains safe given that these patients typically have irregular periods any ways.  Page 11: Tertiary Aims: this section was added incase these found topics are to be published as separate papers outside of the main research paper with the primary and secondary results.  Page 21: Inclusion Criteria: The order of the Rotterdam criteria was changed so that it was clearer of the requirements. A patient must be A+B or A+C to qualify for this diagnosis of PCOS.  Exclusion Criteria: It was decided that patients should washout prior to screening. Therefore most of that language was removed. Deep venous thrombosis, pulmonary embolus, and cerebrovascular accident as well as breast carcinoma are part of hormonal contraceptive contraindications so these topics were removed so that they were not presenting twice.  Page 23: “Letters will be mailed to potential subjects which provides details of the study and contact information of the study team (to allow for subject to reach out for more information). Penn Datastore (and PSU equivalent) may be utilized to provide a list of potential subjects based on clinical practice information.” This was added as a recruitment tool for this study in order to reach patients with in the Hospitals network that may be interested.  Page 24: The consent process has been updated to indicate it will be conducted according to each site’s standard operating procedures as they may different depending on each institution’s interpretation of Shinal v. Tom opinion.  Study medication has been updated to clarify that subjects will receive extra doses of medication at each visit in case they need to utilize the study window. Subjects will be instructed not to use this medication unless approved to do so by the study team. Pill counts will allow the study team to know if extra medication was utilized or not. The decision to give extra medication at each visit was made to ensure that there would be no breaks in compliance of study medication- as breaks could alter the primary outcome.  Lifestyle modification was updated to remove the 500 calorie deficit as this study will not require that subjects cut calories. This description was taken from another study and not edited appropriately the first time.  Page 25: The Neuropsych assessments were edited to eliminate assessments. The study doctors discussed that this was not a primary aim of the protocol and administering so many test could be a burden on study participants. Only the most vital assessments will be administered.  Page 26: The study procedure transvaginal ultrasound was updated to state that it will only be performed if indicated as screening. If the subject has had one in the past 12 month that is acceptable for use. All subjects will however, receive a transvaginal ultrasound at randomization and 24 week visit as these will be used in the primary analysis.  Page 27: Additional questionnaires regarding quality of life were added. It was always the goal to have these questionnaires however specific versions were not decided upon with the first submission of the protocol.  The table was update to match all changes to the protocol for version 2.0.  Page 29-33: Study procedures were updated to order them in the order in which they should be performed and to add clarifying information.  Page 38: Secondary Outcome was updated to be consistent with grant application.  Page 41:  Some AEs were removed as they were copied from a previous protocol and not relevant to this protocol." |
| 1/30/2018 | 3 | 2/6/2018 | "1. We changed all > signs in the inclusion and exclusion criteria to ≥ and < to ≤.  2. We re-introduced, from the Protocol version 1.0, the Digit Symbol Substitution test back into the cognitive testing platform.  3. We edited the screening labs to indicate for 17OH Progesterone and DHEAS, if these labs are drawn within the subject’s lifetime prior to screening that that value can be used to determine eligibility criteria and would not have to be drawn. For Total Testosterone, if the subject’s FG≥8 (≥2 for women of Asian descent) then this lab does not have to be drawn. Eligibility criteria can be determined without this lab value.  4. We edited safety labs to indicate if the labs are drawn within the 2 months prior to the Screening visit then those lab values can be used to determine eligibility criteria. The safety labs would not be drawn at the Screening visit in this circumstance but then must be drawn at Randomization visit. If a subject has these labs drawn at the Screening visit then they do not have them drawn at the Randomization visit.  5. Therefore both the Screening Visit and Randomization visit now include “Safety labs (as needed)”  6. Estradiol was added to the fasting lab tests  7. Early Termination Visit was set to ≥12 weeks instead of 8 weeks" |
| 4/6/2018 | 4 | pending edits | edits listed below |
| 5/1/2018 | 4 | 5/7/2018 | 1. We added that during cognitive testing staff members can audio record the testing with subjects to verify answers given in a timely matter. All audio recordings will be consented for by the subject prior to recording and will remain on file for less than 24 hours at which point they will be deleted. To verify standardization of administration of the tests one patient recording from each certified staff member will go to the Clinical Neuropsychologist.  2. This audio recording has been added as an optional part of the consent form.  3. We edited the Neuropsych assessments description to match the test battery performed.  4. We added in the Female Sexual Distress Scale-Revised questionnaire to be administered at randomization and 24 weeks. This is a screening questionnaire for measuring sexually related personal distress in women with Female Sexual Dysfunction (FSD)  5. We added in the Female Sexual Function Index questionnaire to be administered at randomization and 24 weeks. This is a brief questionnaire measure of sexual function in women. It was developed for the specific purpose of assessing domains of sexual function (e.g. sexual arousal, orgasm, satisfaction, pain) in clinical trials.  6. We made an advertisement for a website with a bullet point focus.  7. We made a square advertisement.  8. We made an advertisement in a video format. |
| 9/24/2018 | 5 | 10/1/2018 | 1) Changed inclusion criteria language to be more board regarding the Rotterdam criteria. The specific criteria then can be edited in the MOP only. This decision is being made based on the Rotterdam criteria changing for a second time since the start of the study. Due to the potential of it changing again we would like to keep the specifics only in the MOP. It is changing from AFC of 18 to 20 and an FG score of 8 to 6. Change made to page 19. |
| 4/11/2019 | 6 | 5/6/2019 | 1) On pages 8, 26, 29, 30, and 34 edits were made to insure patient contact language was consisted throughout the document as there were still some places that stated phone call specifically instead of patient contact in a general since.  2) On page 22 added survey to state that is just not only through phone calls in which patients are prescreened. |
| 5/10/2019 | 7 | 5/20/2019 | On pages 7, 18, 19 edits were made to increase BMI criteria from ≤ 45 kg/m² to ≤ 48 kg/m² |
| 2/5/2020 | 8 | 5/11/2020 | • Removed The Director of Regulatory Affairs (Bridget Nolan). Pages 3, 19, 40, and 41.  • Recruitment clarification for the use of email as well as mail this one sentence was an oversight for the changes made previously to allow contact via email (5/1/19 cover letter). Also added the use of other electronic medical record (EMR) tools such as SlicerDicer and BPA. Page 21.  • Contingent of approval of the Penn/CHOP agreement for recruitment purposes. A line was added to allow recruitment through CHOP similar to the recruitment efforts being made currently at both PSU and Penn. Page 21  • Added line about potential for pregnancy consent to follow until outcome. Page 24.  • Use for transabdominal US added on an if needed bases. Page 25.  • Clarified that the subject may assess their own Hirsutism. Page 25.  • Clarified that the Early Termination visit can occur at ≥ to 12 weeks of being on study medication. Pages 26, 30.  • Throughout the whole document minor formatting changes made. |
| 10/22/2020 | 9 | pending edits | edits listed below |
| 11/30/2020 | 9 | 12/3/2020 | "• Recruitment option added: “A subject referral-based payment structure will be added to increase recruitment. Prior study participants who have given their permission for re-contact that are not Penn Employees will be contacted to give them the opportunity to refer individuals to our study. For each individual referred who qualifies as eligible, the prior participant will get $25. The referral period will last until the study team determines the budget to support this type of referral is no longer available. The study team will inform the prior participants at that time of the ending of the referral-based payment program.” Change on page 21.  • Half of the screening visit along with the 4 week visit and 8 week visits can be completed remotely at the discretion of the PI. This option is being utilize currently to decrease exposure during the COVID 19 pandemic and may need to permanently be utilized as an option until completion of the study. Changes on pages: 25, 26, 28, 29.  • FG≥6 changed. This change reflects a change that was made back in 10/5/2018 by the study team to reflect the current diagnostic PCOS criteria. This one place went unnoticed. Change of page 27.  • Medication compliance and adherence modified to clearly state that these can be obtained via the return from the medication bottles that go to IDS as well as the journal diaries that with get entered into the study database. Either option can provide the study team with the correct statistics needed for analysis for our primary goal to determine the effect of 6 months’ treatment with OCP vs. metformin vs. OCP + metformin on prevalence of MetS and its components in overweight / obese women. Change on page 22." |
| 7/8/2021 | 10 | 7/27/2021 | All edits are in section 5.4.1 of the protocol. Dr. Monica Mainigi added and CHOP added as a recruitment site. |
| 11/30/2021 | 10.1 | 12/22/2021 | Exclusion criteria increased from >14 drinks per week to ≥ 22 drinks per week. |
