# Supplementary material for: Impact of combined hormonal contraceptives and metformin on metabolic syndrome in women with hyperandrogenic polycystic ovary syndrome and obesity: The COMET-PCOS randomized clinical trial
Source: PLoS Med. 2025 Dec 8;22(12):e1004662. doi: 10.1371/journal.pmed.1004662 (PMC12697981; doi:10.1371/journal.pmed.1004662)
Supplement: S2 Text — (DOCX) [file pmed.1004662.s004.docx]

**Statistical Analysis Plan (SAP)**

**Randomization**

Randomization is a critical feature of a clinical trial because it prevents treatment-selection biases. The study statistician developed the programs for the randomization; however, the final random seeds used to generate the randomization scheme were prepared by a statistician in Penn State University’s Department of Public Health Sciences independent of the study in order to keep the study statistician blinded as well. The randomization scheme for this study used variable-size, random permuted blocks to ensure that the number of participants in each treatment arm was balanced after each set of *B* randomized participants, where *B* represents block sizes of 3 or 6. Furthermore, the randomization was stratified by recruitment site (University of Pennsylvania/Penn State University), race (Black/non-Black), and the presence of metabolic syndrome (MetS) at baseline (yes/no). Participants were randomized in a 1:1:1 ratio to continuous combined oral contraceptive pill (COCP) (20μg ethinyl estradiol/0.15mg desogestrel) and placebo, extended-release metformin (XR) and placebo, or COCP and metformin (combined) daily for 24 weeks.

**Sample Size and Power Calculations**

The primary outcome of the COMET-PCOS trial was to assess a linear trend in prevalence of MetS after 24 weeks’ treatment over the 3 arms assuming a 30% prevalence of MetS at baseline (derived from University of Pennsylvania data and the OWL-PCOS trial^1^). We anticipated a 15% subject drop-out over the course of the trial (in OWL-PCOS it was 8.1% over 16 weeks). Based on these assumptions, a sample size of 240 (80 per arm) provided 80% statistical power to detect a linear trend in the prevalence of MetS over the 3 arms at the end of 24 weeks of 26% in the metformin arm, 40% in the COCP+metformin arm and 50% in the COCP arm using a two-sided test for linear trend with a significance level of 0.05.

| Proportion with MetS at the End of the Trial in Metformin Arm | Proportion with MetS at the End of the Trial in COCP+Metformin Arm | Proportion with MetS at the End of the Trial in COCP Arm | Total Sample Size (0% Drop-out) | Total Sample Size (15% Drop-out) | Power (%) | Type I Error (α) |
| --- | --- | --- | --- | --- | --- | --- |
| 0.25 | 0.38 | 0.50 | 204 | 240 | 84 | 0.05 |
| 0.25 | 0.38 | 0.52 | 204 | 240 | 89 | 0.05 |
| **0.26** | **0.40** | **0.50** | **204** | **240** | **80** | **0.05** |
| 0.26 | 0.40 | 0.52 | 204 | 240 | 86 | 0.05 |
| 0.28 | 0.42 | 0.52 | 204 | 240 | 80 | 0.05 |

**Statistical Analysis Methods**

Primary analyses invoked an intent-to-treat paradigm, wherein all randomized subjects are included according to their randomized treatment arm, regardless of actual treatment received, protocol violations, etc. Data were summarized using descriptive statistics for continuous variables (mean, standard deviation, number of observations, and percentiles) and frequency statistics (frequencies and percentages) for categorical variables. The area under the curve (AUC) for glucose and insulin from the OGTT was calculated using the trapezoidal rule per subject. Our calculation of AUC required non-missing fasting (0h) and 2h values and at least 2 of the 3 intermediate time points (30, 60, 90). Calculations of summed scores for questionnaires required non-missing data for all items. Univariate and bivariate distributions were inspected in order to address any missing data, inconsistent responses, outliers, and data entry errors. Data entry values that matched the source document but were suspected to be errors (e.g. suspicious self-reported values from remote visits) were omitted from analyses. The sample size estimates have taken into consideration a participant drop-out of 15%; however, every effort was made during the studies to minimize drop-out. If, study attrition appears to be an issue, we will use the observed data to determine if patients who completed the study differed from those who did not. To control for potential confounding factors for the association of the treatment effects with the metabolic syndrome, we have stratified the randomization by recruitment site, race, and the presence of metabolic syndrome at baseline. Although stratification may potentially yield unequal numbers between strata, within each individual stratum there will be approximately equal numbers and balance between treatment groups. All analyses include the 3 randomization stratification factors as covariates in the statistical models, except for models assessing the change over time for MetS or its individual components which only include recruitment site and race as covariates. All hypothesis tests are two-sided and all analyses were performed using SAS software, version 9.4 (SAS Institute, Inc., Cary, NC) or R software (R Foundation for Statistical Computing, Vienna, Austria).

MetS is defined as presence of at least 3 of the following 5 criteria: TG≥150mg/dl, HDL-C<50mg/dl, BP≥130/≥85mmHg or use of anti-hypertensive medications, waist circumference≥88cm (≥80cm for Asian subjects), and fasting glucose≥100mg/dl. The final calculation of MetS is based on biometric measurements and safety labs obtained at the screening visit (baseline) and last visit completed (end of study). However, additional calculations were performed to be used in sensitivity analyses, which included (1) using biometric measurements and core labs obtained at the randomization visit (baseline) and end of study visit to calculate MetS, (2) calculating end of study MetS based on 24 week visit only, (3) calculating end of study MetS based on 16 week visit only.

For the primary outcome of the presence of the metabolic syndrome at the end of the study, binary logistic regression was used with independent variables that include terms for the treatment arm and the 3 randomization stratification factors as covariates, with a contrast constructed to test for linear trend over the three treatment arms. Following our assessment of the initial fit of the model, we added covariates that correspond to other potential confounders (age and depression/anxiety medication) to assess their impact, if any, on the treatment effects.

A variety of secondary continuous outcomes were collected during this longitudinal trial. These secondary outcomes include serum androgens, cholesterol efflux (HDL-C function) parameters, serum apolipoproteins, lipid particle size and number, anthropometric measures (BMI, adipokines, biomarkers of inflammation (e.g., hsCRP), measures of adipose tissue, abdominal adiposity, and quality of life measures (PCOSQ). For these continuous outcomes, linear mixed-effects models were used to assess differences between the treatment arms with respect to changes in these outcomes over time. The independent fixed variables in the model will be treatment arm, time, the interaction of treatment and time, and the 3 randomization stratification factors as covariates. These linear mixed models included a random subject effect and a first-order antedependence variance-covariance structure for the repeated visits over time. In the event the model did not converge, the random subject effect was removed and the first-order antedependence variance-covariance structure for the repeated visits over time was kept. From the mixed-effects models, contrasts were constructed to test the hypotheses of interest with respect to changes over time in the outcomes. Linear mixed-effects models are an extension of ordinary regression models that account for the between- and within-subject correlation inherent in longitudinal trials. Further, linear mixed-effects models do not drop patients with incomplete data and are easily extended to nonlinear mixed-effects models for ordinal data and count outcomes. Residual diagnostics were assessed to determine the appropriateness of the model fit and, if necessary, transformations (e.g., logarithmic) of the response was used to meet parametric modeling assumptions. Differences in estimated marginal means and associated 95% confidence intervals (CIs), or ratio of geometric marginal means and associated 95% CIs in the event a logarithmic transformation is necessary, were used to quantify the magnitude of the effects.

For any binary outcomes collected at each visit, analyses were based on generalized estimating equations (GEE) with a logit link, an extension of logistic regression that accounts for correlated data within-subjects inherent in longitudinal trials, with independent variables that include terms for the treatment arm, time, the interaction of treatment and time, and the randomization stratification factors as covariates. The GEE models for MetS and its components only include the 2 randomization stratification factors of site and race as covariates; the metabolic syndrome randomization stratification factor is omitted, since a baseline MetS value is already included in the repeated measures modeling. The effect size were quantified using the odds ratios (OR) and corresponding 95% CI.

Freeman-Halton tests were used to compare adverse event proportions among the groups.

**Early Termination Impact on Analysis**

Per the protocol, subjects terminating the study prior to 24 weeks who completed the first 12 weeks of the intervention could complete an early termination visit. Upon completion of the study, we found that no early termination visits were completed between 12 and 16 weeks. However, subjects who terminated after completion of 16 week visit or completed an early termination visit between 16-24 weeks contributed to end of study outcome data, i.e., end of study corresponds to data from last completed visit ≥16 weeks.

References

1. Legro RS, Dodson WC, Kris-Etherton PM, Kunselman AR, Stetter CM, Williams NI, Gnatuk CL, Estes SJ, Fleming J, Allison KC, Sarwer DB, Coutifaris C, Dokras A. Randomized controlled trial of preconception interventions in infertile women with polycystic ovary syndrome. J Clin Endocrinol Metab. 2015 Nov;100(11):4048-58.
