# Supplementary material for: Impact of combined hormonal contraceptives and metformin on metabolic syndrome in women with hyperandrogenic polycystic ovary syndrome and obesity: The COMET-PCOS randomized clinical trial
Source: PLoS Med. 2025 Dec 8;22(12):e1004662. doi: 10.1371/journal.pmed.1004662 (PMC12697981; doi:10.1371/journal.pmed.1004662)
Supplement: S3 Text — (DOCX) [file pmed.1004662.s005.docx]

**Protocol Deviation Report:**

On the following pages, protocol deviations reported during the study are listed individually with a brief description.

COVID-19 protocol deviations are reported separately. This includes all remote visits due to pandemic restrictions through 12/3/2020. The IRB approved an exception for remote visits to be conducted on 4/7/2020.

As of 12/3/2020, the protocol now allows for specific remote visits at the discretion of the PI as well as pill compliance calculations based on journal diaries. Therefore deviations will no longer need to be recorded in these situations. As of this date, we will record all deviations on the main report; no further deviations will be recorded as COVID-19.

In summary, there have been a total of 201 deviations (147 Penn, 54 PSU), of which 87 were specific to COVID-19. Since the prior report (August 2023), one data entry error of a protocol deviation has been removed and one new protocol deviation has been recorded.

All occurrences are deviations only; no protocol violations have been reported. There has been one protocol exception for enrollment into the study. A TSH level

> 4.5 mIU/mL is an exclusion criteria. On 10/22/18, Dr. Dokras approved a

protocol exception for enrollment of a Penn subject with a TSH level of 4.58.

***Protocol Deviation Report***

| **Study ID** | **Event Name** | **Date of occurrence:** | **Description of occurrence** | **Compromised the scientific integrity of the data collected for the study?** | **Significant or substantive risk of harm to the research subject?** | **Willful or knowing breach of human subject protection regulations, policies, or procedures on the part of the investigator(s)?** | **Serious or continuing noncompliance with federal, state, local or institutional human subject protection regulations, policies, or procedures?** | **Inconsistent with the NIH**  **Human Research Protection Program? research, medical, and ethical principles?** | **Affects the rights, safety, or welfare of this subject or the integrity of research data (scientific soundness)?** |
| --- | --- | --- | --- | --- | --- | --- | --- | --- | --- |
| 100-224 | 24 Week Visit | 2023-12-01 | Fasting blood, or OGTT not collected. Patient was unable to tolerate needle sticks after 2 tried, and was not willing to come back. Safety labs collected prior to IV stopping working. | No | No | No | No | No | is not believed  to |
| 100-227 | 8 Week Visit | 2023-07-15 | Subject has been taking 5 tablets of Metformin/placebo instead of 4 tablets | No | No | No | No | No | is not believed  to |
| 100-208 | Randomization | 2023-03-23 | Discovered on 8/2/23 that subject 100-208 was incorrectly logged and randomized as 100-028 by IDS, and to the wrong stratification factor. Subject 100-208 was randomized as NO Metabolic Syndrome instead of YES Metabolic Syndrome on  3/23/23. Randomization for ID 208 occurred in redcap under the wrong ID (028 who was a screen failure). The Randomization form under 028 will be deleted from redcap, and the Randomization form for 208 will record the treatment assignment she was given using the "back-up" field (this is the only way to assign the correct treatment assignment to 208 in redcap). | No | No | No | No | No | is not believed  to |

***Protocol Deviation Report***

| **Study ID** | **Event Name** | **Date of occurrence:** | **Description of occurrence** | **Compromised the scientific integrity of the data collected for the study?** | **Significant or substantive risk of harm to the research subject?** | **Willful or knowing breach of human subject protection regulations, policies, or procedures on the part of the investigator(s)?** | **Serious or continuing noncompliance with federal, state, local or institutional human subject protection regulations, policies, or procedures?** | **Inconsistent with the NIH**  **Human Research Protection Program? research, medical, and ethical principles?** | **Affects the rights, safety, or welfare of this subject or the integrity of research data (scientific soundness)?** |
| --- | --- | --- | --- | --- | --- | --- | --- | --- | --- |
| 100-191 | 24 Week Visit | 2023-02-28 | ASAS24 Dietary recall not filled out at 24W timepoint | No | No | No | No | No | is not believed  to |
| 100-187 | 24 Week Visit | 2023-02-09 | ASA24 dietary recall not filled out at 24W timepoint | No | No | No | No | No | is not believed  to |
| 100-191 | 12 Week PC | 2022-12-08 | ASA24 Dietary recall not filled out at 12W timepoint | No | No | No | No | No | is not believed  to |
| 100-169 | 24 Week Visit | 2022-10-14 | DXA, OGTT, labs, and TVU missed due to remove 24W visit. | No | No | No | No | No | is not believed  to |
| 100-189 | 4 Week Visit | 2022-09-04 | Subject did not start medications as directed. 4 days 1 tablet, 1  day 2 tablets, then to 3 for 5 and then to 4 as direct. | No | No | No | No | No | is not believed  to |
| 100-188 | 4 Week Visit | 2022-08-20 | At 2 weeks BHI reports "forgetful" at taking meds. By 9/19/22 had only taken a few weeks worth. Did not step up Metformin per instructions. | No | No | No | No | No | is not believed  to |
| 200-103 | 24 Week Visit | 2022-08-15 | Patient did not report to 35 Hope Drive for her TVU first thing in the morning, she went to the CRC first. SC completed entire final visit in the CRC then asked patient to go to 35 Hope Drive for TVU and patient refused to go to the clinic and refused the TVU for the final visit. | No | No | No | No | No | is not believed  to |

***Protocol Deviation Report***

| **Study ID** | **Event Name** | **Date of occurrence:** | **Description of occurrence** | **Compromised the scientific integrity of the data collected for the study?** | **Significant or substantive risk of harm to the research subject?** | **Willful or knowing breach of human subject protection regulations, policies, or procedures on the part of the investigator(s)?** | **Serious or continuing noncompliance with federal, state, local or institutional human subject protection regulations, policies, or procedures?** | **Inconsistent with the NIH**  **Human Research Protection Program? research, medical, and ethical principles?** | **Affects the rights, safety, or welfare of this subject or the integrity of research data (scientific soundness)?** |
| --- | --- | --- | --- | --- | --- | --- | --- | --- | --- |
| 100-151 | 24 Week Visit | 2022-04-29 | No DEXA for 24W visit. DEXA machine in service at CHPS | No | No | No | No | No | is not believed  to |
| 100-172 | Randomization | 2022-04-29 | No DEXA for RAN visit. DEXA machine being serviced at CHPS | No | No | No | No | No | is not believed  to |
| 100-157 | 12 Week PC | 2022-04-19 | No response from AMR at 12W. Upper window ended 4/19/2022 | No | No | No | No | No | is not believed  to |
| 100-153 | 24 Week Visit | 2022-04-14 | No DEXA for 24 W visit. DEXA machine broken and needs repair at CHPS | No | No | No | No | No | is not believed  to |
| 200-104 | 4 Week Visit | 2022-03-29 | Study coordinator reviewed study logs and noted that patient did dose up correctly with 2 tablets for 5 days of metformin. On 3/12 patient should have stepped up to 3 tablets but missed taking Metformin. On 3/13 patient should have taken 3 tablets but took 2 tablets and then stepped up to 3 on 3/14/2022. | No | No | No | No | No | is not believed  to |
| 200-103 | 4 Week Visit | 2022-03-22 | Study Coordinator reviewed patient logs at visit and noticed patient took 1 tablet of Metformin for 6 days instead of 5 days.  Patient verbalized she realized she made a mistake with the dosing schedule. | No | No | No | No | No | is not believed  to |

***Protocol Deviation Report***

| **Study ID** | **Event Name** | **Date of occurrence:** | **Description of occurrence** | **Compromised the scientific integrity of the data collected for the study?** | **Significant or substantive risk of harm to the research subject?** | **Willful or knowing breach of human subject protection regulations, policies, or procedures on the part of the investigator(s)?** | **Serious or continuing noncompliance with federal, state, local or institutional human subject protection regulations, policies, or procedures?** | **Inconsistent with the NIH**  **Human Research Protection Program? research, medical, and ethical principles?** | **Affects the rights, safety, or welfare of this subject or the integrity of research data (scientific soundness)?** |
| --- | --- | --- | --- | --- | --- | --- | --- | --- | --- |
| 200-100 | 24 Week Visit | 2022-03-14 | Cognitive testing could not be completed because Study Coordinator had to leave patient visit for a family emergency. | No | No | No | No | No | is not believed  to |
| 100-145 | 24 Week Visit | 2022-01-20 | Subject in for 24W visit 1 day out of the window due to JSC having COVID and needing to wait two weeks. The visit window was until 1/19/22. Also, 30-, 60-, and 90-minute OGTT blood was collected as serum (gold top), not Li-Hp. 0- and 120-minute OGTT were on Li-Hp (green top) | No | No | No | No | No | is not believed  to |
| 100-097 | 24 Week Visit | 2021-12-22 | ASA24 not completed | No | No | No | No | No | is not believed  to |
| 200-101 | 4 Week Visit | 2021-12-10 | SC was reviewing patient logs and noticed she missed her meds on 11/24/2021. Patient should have taken 3 tablets on 11/25, 11/26, 11/27 and 11/28, but did not therefore, not stepping up correctly even though instructed about this by SC at randomization visit. | No | No | No | No | No | is not believed  to |
| 100-150 | 8 Week Visit | 2021-12-06 | UPT result and BP measurements never sent to CRC for remote 8 Week visit. | No | No | No | No | No | is not believed  to |

***Protocol Deviation Report***

| **Study ID** | **Event Name** | **Date of occurrence:** | **Description of occurrence** | **Compromised the scientific integrity of the data collected for the study?** | **Significant or substantive risk of harm to the research subject?** | **Willful or knowing breach of human subject protection regulations, policies, or procedures on the part of the investigator(s)?** | **Serious or continuing noncompliance with federal, state, local or institutional human subject protection regulations, policies, or procedures?** | **Inconsistent with the NIH**  **Human Research Protection Program? research, medical, and ethical principles?** | **Affects the rights, safety, or welfare of this subject or the integrity of research data (scientific soundness)?** |
| --- | --- | --- | --- | --- | --- | --- | --- | --- | --- |
| 200-095 | 20 Week PC | 2021-11-11 | After multiple attempts via email and leaving a phone message to have PT respond for Week 20 PC patient never responded to SC. | No | No | No | No | No | is not believed  to |
| 100-153 | Randomization | 2021-10-21 | Did not complete cognitive test or blood work at today's visit. Plan to finish visit on 10/28/21.  Randomization and medication dispensing will happen on the 10/28/21 visit as well. | No | No | No | No | No | is not believed  to |
| 200-095 | 8 Week Visit | 2021-08-11 | Patient finally returned her Week 4 study logs at this visit. Upon review SC noted that she did not step up with the Metformin study meds per instructions given to her at the randomization visit. Patient reported she did not follow the instructions given to her by the Study Coordinator. She stepped up the Metformin after 7 days instead of 5 days. | No | No | No | No | No | is not believed  to |
| 100-123 | 24 Week Visit | 2021-08-10 | Threw up during OGTT so only the fasting labs and timepoint zero for the OGTT were collected. The 30, 60, 90, and 120-minute blood for the OGTT were not performed. | No | No | No | No | No | is not believed  to |

***Protocol Deviation Report***

| **Study ID** | **Event Name** | **Date of occurrence:** | **Description of occurrence** | **Compromised the scientific integrity of the data collected for the study?** | **Significant or substantive risk of harm to the research subject?** | **Willful or knowing breach of human subject protection regulations, policies, or procedures on the part of the investigator(s)?** | **Serious or continuing noncompliance with federal, state, local or institutional human subject protection regulations, policies, or procedures?** | **Inconsistent with the NIH**  **Human Research Protection Program? research, medical, and ethical principles?** | **Affects the rights, safety, or welfare of this subject or the integrity of research data (scientific soundness)?** |
| --- | --- | --- | --- | --- | --- | --- | --- | --- | --- |
| 100-128 | 12 Week PC | 2021-06-30 | SFT responded to 12 W contact out of window. Upper window ended 6/28/2021 | No | No | No | No | No | is not believed  to |
| 200-092 | Randomization | 2021-06-08 | Study Coordinator forgot to pull a purple top EDTA tube for the NIH sample. As a result, serum and blood were not obtained for this fasting blood work from the patient. | No | No | No | No | No | is not believed  to |
| 100-128 | 8 Week Visit | 2021-06-02 | SFT sent 8W data for weight, UPT, and pill counts on 6/1/21. SFT sent hip/waist and BP data on 6/2/21. All other data sent in the study window. | No | No | No | No | No | is not believed  to |
| 200-094 | Randomization | 2021-05-21 | TVU not performed at randomization visit. Documentation of TVU obtained from INSUPP study on 10/2/2020 used at screening to determine PCOS dx. Study Coordinator inadvertently forgot to repeat TVU at randomization. | No | No | No | No | No | is not believed  to |

***Protocol Deviation Report***

| **Study ID** | **Event Name** | **Date of occurrence:** | **Description of occurrence** | **Compromised the scientific integrity of the data collected for the study?** | **Significant or substantive risk of harm to the research subject?** | **Willful or knowing breach of human subject protection regulations, policies, or procedures on the part of the investigator(s)?** | **Serious or continuing noncompliance with federal, state, local or institutional human subject protection regulations, policies, or procedures?** | **Inconsistent with the NIH**  **Human Research Protection Program? research, medical, and ethical principles?** | **Affects the rights, safety, or welfare of this subject or the integrity of research data (scientific soundness)?** |
| --- | --- | --- | --- | --- | --- | --- | --- | --- | --- |
| 100-134 | Randomization | 2021-04-21 | Subject originally randomized on 4/21/21 with MS and non AA criteria marked. Mistake found on 7/9/21. The subject should have been randomized under No MS and non AA criteria. Correction to be made using manual REDCap entry. | No | No | No | No | No | is not believed  to |
| 100-112 | 24 Week Visit | 2021-04-09 | ASA24 not completed | No | No | No | No | No | is not believed  to |
| 200-080 | Early Termination Visit | 2021-03-01 | Patient withdrew from the study a few days before her 16 Week visit due to personal constraints. She only had a small window of time to come in for the early termination visit. As a result, she declined OGTT, DXA, TVU and cognitive testing. Study Coordinator requested patient do these items at a later date or remotely if the items could be done remotely and patient refused to do anything else. | No | No | No | No | No | is not believed  to |
| 100-100 | 24 Week Visit | 2021-01-13 | ASA24 not completed | No | No | No | No | No | is not believed  to |

***Protocol Deviation Report***

| **Study ID** | **Event Name** | **Date of occurrence:** | **Description of occurrence** | **Compromised the scientific integrity of the data collected for the study?** | **Significant or substantive risk of harm to the research subject?** | **Willful or knowing breach of human subject protection regulations, policies, or procedures on the part of the investigator(s)?** | **Serious or continuing noncompliance with federal, state, local or institutional human subject protection regulations, policies, or procedures?** | **Inconsistent with the NIH**  **Human Research Protection Program? research, medical, and ethical principles?** | **Affects the rights, safety, or welfare of this subject or the integrity of research data (scientific soundness)?** |
| --- | --- | --- | --- | --- | --- | --- | --- | --- | --- |
| 200-081 | 12 Week PC | 2021-01-11 | Week 12 PC follow-up outside of visit window. Contact should have taken place between 12/17/2020 - 12/31/2020 and did not occur until 1/11/2021. | No | No | No | No | No | is not believed  to |
| 100-113 | 8 Week Visit | 2021-01-08 | UPT results not returned subject lost to follow up. | No | No | No | No | . | is not believed  to |
| 100-116 | 8 Week Visit | 2021-01-07 | Subject unable to get UPT to work at 8 week visit. CRC Vresilovic sent new UPT to subject due to holidays, mailing delays, negative result collected outside visit window. | No | No | No | No | No | is not believed  to |
| 200-086 | 4 Week Visit | 2021-01-05 | Patient arrived for Week 4 visit. Study Coordinator reviewed dosing up of Metformin. Patient took 3 tablets for 6 days instead of 5 days. Patient reports she knew she made a mistake and that was her fault, but made correction the next day. | No | No | No | No | No | is not believed  to |
| 200-080 | 4 Week Visit | 2020-12-04 | Study Coordinator reviewed patient's logs and noticed that she dosed 3 tablets for 5 days correctly, but made a mistake and dosed at 3 tablets for an extra day instead of stepping up to 4 tablets, but then dosed up to 4 tablets after noticing her mistake. | No | No | No | No | No | is not believed  to |

***Protocol Deviation Report***

| **Study ID** | **Event Name** | **Date of occurrence:** | **Description of occurrence** | **Compromised the scientific integrity of the data collected for the study?** | **Significant or substantive risk of harm to the research subject?** | **Willful or knowing breach of human subject protection regulations, policies, or procedures on the part of the investigator(s)?** | **Serious or continuing noncompliance with federal, state, local or institutional human subject protection regulations, policies, or procedures?** | **Inconsistent with the NIH**  **Human Research Protection Program? research, medical, and ethical principles?** | **Affects the rights, safety, or welfare of this subject or the integrity of research data (scientific soundness)?** |
| --- | --- | --- | --- | --- | --- | --- | --- | --- | --- |
| 200-084 | 4 Week Visit | 2020-11-20 | Upon review of patient's logs, study coordinator noted that instead of patient stepping up to 2 tablets of Metformin for 5 days she stepped up for 6 days. Then instead of stepping up to 3 tablets per day for 5 days patient only stepped up for 4 days. Patient knew she made a mistake even though she was instructed carefully at the randomization visit by study coordinator. | No | No | No | No | No | is not believed  to |
| 200-083 | 4 Week Visit | 2020-11-18 | At week 4 visit during the review of patient logs, study coordinator noted patient did not step up from 2 Metformin tablets to 3 tablets correctly. She only took 1 day of 3 tablets instead of 5 days and instead of jumped right up to 4 tablets. Patient was counseled about this. She said she simply did not follow the instructions study coordinator gave her at the randomization visit. | No | No | No | No | No | is not believed  to |

***Protocol Deviation Report***

| **Study ID** | **Event Name** | **Date of occurrence:** | **Description of occurrence** | **Compromised the scientific integrity of the data collected for the study?** | **Significant or substantive risk of harm to the research subject?** | **Willful or knowing breach of human subject protection regulations, policies, or procedures on the part of the investigator(s)?** | **Serious or continuing noncompliance with federal, state, local or institutional human subject protection regulations, policies, or procedures?** | **Inconsistent with the NIH**  **Human Research Protection Program? research, medical, and ethical principles?** | **Affects the rights, safety, or welfare of this subject or the integrity of research data (scientific soundness)?** |
| --- | --- | --- | --- | --- | --- | --- | --- | --- | --- |
| 100-115 | Randomization | 2020-10-27 | The patient was not comfortable with having an IV placed or blood drawn at the visit on 10/27/20 due to two unsuccessful attempts.  CRC discussed options for returning another day to complete the Randomization visit. On 10/27/20 100-115 was not randomized. No TVUS, DXA, Blood work, cognitive test, or LMC performed.  Vitals/biometrics and paper questionnaires/surveys completed. | No | No | No | No | No | is not believed  to |
| 100-110 | Randomization PC | 2020-09-28 | NMM took 2 tablets for 4 days, instead of 5 days, for metformin build up | No | No | No | No | No | is not believed  to |
| 100-105 | 8 Week Visit | 2020-09-14 | Patient took 5 tablets of Metformin for 6 days instead of the prescribed 4 tablets Metformin/Placebo. | No | No | No | No | No | is not believed  to |
| 100-108 | Randomization PC | 2020-09-10 | inappropriate starting of study medications. CRC and subject made a new plan for dosage to ensure for side effects to not be heightened. | No | No | No | No | No | is not believed  to |

***Protocol Deviation Report***

| **Study ID** | **Event Name** | **Date of occurrence:** | **Description of occurrence** | **Compromised the scientific integrity of the data collected for the study?** | **Significant or substantive risk of harm to the research subject?** | **Willful or knowing breach of human subject protection regulations, policies, or procedures on the part of the investigator(s)?** | **Serious or continuing noncompliance with federal, state, local or institutional human subject protection regulations, policies, or procedures?** | **Inconsistent with the NIH**  **Human Research Protection Program? research, medical, and ethical principles?** | **Affects the rights, safety, or welfare of this subject or the integrity of research data (scientific soundness)?** |
| --- | --- | --- | --- | --- | --- | --- | --- | --- | --- |
| 200-078 | 4 Week Visit | 2020-09-02 | Patient came in for Week 4 Visit. SC reviewed logs and noted that patient did not step up with Metformin correctly on 8/15/2020 and again on 8/22/2020. Patient admits she did not follow SC instructions correctly given during randomization visit. | No | No | No | No | No | is not believed  to |
| 200-064 | 24 Week Visit | 2020-08-28 | Patient arrived for Week 24 visit and reported that she did not bring her study medication bottles with her to the visit. Medications were never returned because patient had a house fire and could not get in to get the bottles. | No | No | No | No | No | is not believed  to |
| 200-067 | 4 Week Visit | 2020-08-06 | Patient came in for Week 4 visit. Study coordinator noticed during the review of the study logs that on 7/8/2020 patient did not step up to 2 metformin tablets as she was instructed to do during the randomization visit. Patient said she forgot and never stopped until 7/10/2020. Then patient told SC that she decided on her own to step back to 3 tablets on 7/25/2020 after being on 4 tablets due to side effects and she never contacted the study coordinator. | No | No | No | No | No | is not believed  to |

***Protocol Deviation Report***

| **Study ID** | **Event Name** | **Date of occurrence:** | **Description of occurrence** | **Compromised the scientific integrity of the data collected for the study?** | **Significant or substantive risk of harm to the research subject?** | **Willful or knowing breach of human subject protection regulations, policies, or procedures on the part of the investigator(s)?** | **Serious or continuing noncompliance with federal, state, local or institutional human subject protection regulations, policies, or procedures?** | **Inconsistent with the NIH**  **Human Research Protection Program? research, medical, and ethical principles?** | **Affects the rights, safety, or welfare of this subject or the integrity of research data (scientific soundness)?** |
| --- | --- | --- | --- | --- | --- | --- | --- | --- | --- |
| 200-064 | 16 Week Visit | 2020-07-03 | Patient arrived for Week 16 visit and reported that she forgot both her medication bottles from the last visit. Medication was never returned. | No | No | No | No | No | is not believed  to |
| 100-094 | 4 Week Visit | 2020-06-27 | inappropriate starting of study medications: 6 days at 1 MET  1 day at 2 MET 4 days st 3 MET then to 4 tablets daily MET Only 1 day of diarrhea so not considered overdose as no bad side effects occurred just patient non-compliance. CRC gave patient instructions at RAN visit and spoke about the instructions again at 2W visit. | No | No | No | No | No | is not believed  to |
| 100-096 | 4 Week Visit | 2020-06-24 | inappropriate starting of study medications: 9 days at 2 MET  1 day at 3 MET then to 4 tablets daily MET. patient  non-compliance. CRC gave patient instructions at RAN visit and spoke about the instructions again at 2W visit. | No | No | No | No | No | is not believed  to |
| 100-087 | 16 Week Visit | 2020-06-12 | IDS no pill count returned for OCP only. Therefore no pill compliance completed for OCP only | No | No | No | No | No | is not believed  to |

***Protocol Deviation Report***

| **Study ID** | **Event Name** | **Date of occurrence:** | **Description of occurrence** | **Compromised the scientific integrity of the data collected for the study?** | **Significant or substantive risk of harm to the research subject?** | **Willful or knowing breach of human subject protection regulations, policies, or procedures on the part of the investigator(s)?** | **Serious or continuing noncompliance with federal, state, local or institutional human subject protection regulations, policies, or procedures?** | **Inconsistent with the NIH**  **Human Research Protection Program? research, medical, and ethical principles?** | **Affects the rights, safety, or welfare of this subject or the integrity of research data (scientific soundness)?** |
| --- | --- | --- | --- | --- | --- | --- | --- | --- | --- |
| 200-058 | Early Termination Visit | 2020-03-02 | DXA not completed because patient again insisted she had to leave and must get to an appointment. She once again declined to have the DXA done. | No | No | No | No | No | is not believed  to |
| 200-051 | 24 Week Visit | 2020-02-27 | DXA scan was unable to be performed because patient had to leave the visit abruptly to pick up child. | No | No | No | No | No | is not believed  to |
| 100-074 | 16 Week Visit | 2020-02-19 | Pill count documentation was not completed for this visit. This finding was noted during the OCR Audit summer 2020 | No | No | No | No | No | is not believed  to |
| 100-070 | 24 Week Visit | 2020-02-12 | IDS no pill count returned. Therefore no pill compliance completed | No | No | No | No | No | is not believed  to |
| 200-056 | 16 Week Visit | 2020-02-11 | Patient arrived for Week 16 visit and reported that she forgot her medication bottles from the last visit. Medications were never returned. | No | No | No | No | No | is not believed  to |
| 100-084 | 8 Week Visit | 2020-02-10 | IDS no pill count returned. Therefore no pill compliance completed | No | No | No | No | No | is not believed  to |

***Protocol Deviation Report***

| **Study ID** | **Event Name** | **Date of occurrence:** | **Description of occurrence** | **Compromised the scientific integrity of the data collected for the study?** | **Significant or substantive risk of harm to the research subject?** | **Willful or knowing breach of human subject protection regulations, policies, or procedures on the part of the investigator(s)?** | **Serious or continuing noncompliance with federal, state, local or institutional human subject protection regulations, policies, or procedures?** | **Inconsistent with the NIH**  **Human Research Protection Program? research, medical, and ethical principles?** | **Affects the rights, safety, or welfare of this subject or the integrity of research data (scientific soundness)?** |
| --- | --- | --- | --- | --- | --- | --- | --- | --- | --- |
| 100-078 | 8 Week Visit | 2020-01-08 | The subject took 4000mg of Metformin. The subject reported thinking she forgot to take the morning dose with breakfast, so she took it again with dinner.  100-078 had diarrhea and vomiting immediately and then felt better. 100-078 took the dose twice that day by accident. Did not take the medication 1/9/20 and then back to normal 2000mg daily on 1/10/20. | No | No | No | No | No | is not believed  to |
| 200-042 | 24 Week Visit | 2019-12-06 | Patient arrived for Week 24 visit and reported that she forgot both her medication bottles from the last visit. Medications were never returned. | No | No | No | No | No | is not believed  to |
| 200-058 | Randomization | 2019-11-11 | DXA scan not performed because patient had another appointment and insisted she had to leave. | No | No | No | No | No | is not believed  to |
| 100-057 | 16 Week Visit | 2019-10-11 | IDS no pill count returned for OCPs only. Therefore no pill compliance for the OCPs | No | No | No | No | No | is not believed  to |
| 200-048 | 4 Week Visit | 2019-09-30 | Patient arrived for Week 4 visit and reported that she forgot both her medication bottles from the last visit. Medication were never returned. | No | No | No | No | No | is not believed  to |

***Protocol Deviation Report***

| **Study ID** | **Event Name** | **Date of occurrence:** | **Description of occurrence** | **Compromised the scientific integrity of the data collected for the study?** | **Significant or substantive risk of harm to the research subject?** | **Willful or knowing breach of human subject protection regulations, policies, or procedures on the part of the investigator(s)?** | **Serious or continuing noncompliance with federal, state, local or institutional human subject protection regulations, policies, or procedures?** | **Inconsistent with the NIH**  **Human Research Protection Program? research, medical, and ethical principles?** | **Affects the rights, safety, or welfare of this subject or the integrity of research data (scientific soundness)?** |
| --- | --- | --- | --- | --- | --- | --- | --- | --- | --- |
| 100-068 | 8 Week Visit | 2019-09-25 | Pill bottles were not returned for this visit. Therefore pill count documentation was not completed. This finding was noted during the OCR Audit summer 2020 | No | No | No | No | No | is not believed  to |
| 200-051 | 4 Week Visit | 2019-09-17 | Patient should have dosed up to 3 tablets per day, on 9/17/2019, but she only dosed 2 tablets. Patient realized her error and made correction on 9/18/2019. | No | No | No | No | No | is not believed  to |
| 100-031 | Screening | 2019-08-23 | It was noted during the OCR audit Summer 2020 that this subject did not put a date on the consent next to their signature. The CRC signed and dated the form. The subject was a screenfailure and thus this mistake could no tbe corrected at a future visit. | No | No | No | No | No | is not believed  to |
| 100-046 | 20 Week PC | 2019-07-11 | subject early term and never returned medication bottles. Uncle was dying and subject very distracted during that time. | No | No | No | No | No | is not believed  to |
| 100-047 | 20 Week PC | 2019-06-27 | 20 week contact out of window due to subject noncompliance. Contacted on 6/7/19 no response until 6/27/19. Finding of 2020 monitoring. | No | No | No | No | No | is not believed  to |

***Protocol Deviation Report***

| **Study ID** | **Event Name** | **Date of occurrence:** | **Description of occurrence** | **Compromised the scientific integrity of the data collected for the study?** | **Significant or substantive risk of harm to the research subject?** | **Willful or knowing breach of human subject protection regulations, policies, or procedures on the part of the investigator(s)?** | **Serious or continuing noncompliance with federal, state, local or institutional human subject protection regulations, policies, or procedures?** | **Inconsistent with the NIH**  **Human Research Protection Program? research, medical, and ethical principles?** | **Affects the rights, safety, or welfare of this subject or the integrity of research data (scientific soundness)?** |
| --- | --- | --- | --- | --- | --- | --- | --- | --- | --- |
| 100-056 | 4 Week Visit | 2019-06-25 | Subject forgot to bring the medications back for this visit. | No | No | No | No | No | is not believed  to |
| 100-045 | 20 Week PC | 2019-04-30 | Subject moved to paris and did not return study medications | No | No | No | No | No | is not believed  to |
| 200-022 | 24 Week Visit | 2019-04-16 | Patient was unable to complete the cognitive testing because she got sick after drinking the glucola for the OGTT. | No | No | No | No | No | is not believed  to |
| 100-032 | 24 Week Visit | 2019-03-28 | A discussion with the new monitor and the PI took place on 5/29/2020 that made this missing portion of the OGTT test a finding. This subject threw up during the OGTT at the 24W visit. The 30, 60, 90, and 120 minute time points were not collected | No | No | No | No | No | is not believed  to |
| 200-033 | 4 Week Visit | 2019-03-24 | Patient was scheduled to step up to 2 metformin tablets on 3/24/2019, but forgot to take second pill that day. | No | No | No | No | No | is not believed  to |
| 100-033 | 24 Week Visit | 2019-03-13 | Safety labs for the 24W visit were transferred at 16:16 on 3/12/19.  Due to lab error blood not processed until 3/13/19 at 12:58 as a result a glucose value was not given for the CMP. On 1/11/19 glucose was 78mg/dL. | No | No | No | No | No | is not believed  to |

***Protocol Deviation Report***

| **Study ID** | **Event Name** | **Date of occurrence:** | **Description of occurrence** | **Compromised the scientific integrity of the data collected for the study?** | **Significant or substantive risk of harm to the research subject?** | **Willful or knowing breach of human subject protection regulations, policies, or procedures on the part of the investigator(s)?** | **Serious or continuing noncompliance with federal, state, local or institutional human subject protection regulations, policies, or procedures?** | **Inconsistent with the NIH**  **Human Research Protection Program? research, medical, and ethical principles?** | **Affects the rights, safety, or welfare of this subject or the integrity of research data (scientific soundness)?** |
| --- | --- | --- | --- | --- | --- | --- | --- | --- | --- |
| 200-029 | 4 Week Visit | 2019-02-16 | On 2/16/2019 patient was scheduled to step up to 4 metformin study tablets, but took 3 tablets instead. Correct dose taken the following day 2/17/2019. | No | No | No | No | No | is not believed  to |
| 100-046 | 4 Week Visit | 2019-02-07 | forgot to get hip and waist measurement at the 4W visit. Notified the subject. She may try to get measurements at home today. | No | No | No | No | No | is not believed  to |
| 100-045 | 4 Week Visit | 2019-01-17 | IDS never returned pill count for these returned medications. Pill compliance not able to be completed. | No | No | No | No | No | is not believed  to |
| 100-034 | 8 Week Visit | 2019-01-07 | medications not returned. Term subject | No | No | No | No | No | is not believed  to |
| 100-039 | 8 Week Visit | 2019-01-03 | Patient did not return drug therefore no pill compliance complete | No | No | No | No | . | is not believed  to |
| 200-027 | 4 Week Visit | 2018-12-19 | Patient came in for Week 4 visit. While reviewing step up of Metformin compliance patient informed study coordinator that she took 6 days of Metformin at 2 tablets instead of 5 days. | No | No | No | No | No | is not believed  to |

***Protocol Deviation Report***

| **Study ID** | **Event Name** | **Date of occurrence:** | **Description of occurrence** | **Compromised the scientific integrity of the data collected for the study?** | **Significant or substantive risk of harm to the research subject?** | **Willful or knowing breach of human subject protection regulations, policies, or procedures on the part of the investigator(s)?** | **Serious or continuing noncompliance with federal, state, local or institutional human subject protection regulations, policies, or procedures?** | **Inconsistent with the NIH**  **Human Research Protection Program? research, medical, and ethical principles?** | **Affects the rights, safety, or welfare of this subject or the integrity of research data (scientific soundness)?** |
| --- | --- | --- | --- | --- | --- | --- | --- | --- | --- |
| 200-011 | 24 Week Visit | 2018-12-14 | Patient arrived for Week 24 visit and reported that she forgot both her medication bottles from the last visit. Medications were never returned.. | No | No | No | No | No | is not believed  to |
| 100-039 | 4 Week Visit | 2018-12-06 | Patient did not return drug therefore no pill compliance complete | No | No | No | No | No | is not believed  to |
| 200-023 | 4 Week Visit | 2018-12-05 | Study Coordinator noticed while reviewing study logs at Week 4 visit that patient did not step up per study instructions with metformin correctly. Patient took 1 tab for 7 days, then 2 tabs for 7 days, then 3 tabs for 7 days and is currently at 4 tabs. Patient had all the forms and was given detailed instructions about how to step up correctly. Patient said she did not listen closely and did not refer to study documents. | No | No | No | No | No | is not believed  to |
| 200-010 | 24 Week Visit | 2018-12-03 | Patient arrived for Week 24 visit and reported that she forgot both her medication bottles from the last visit. Medications were never returned. | No | No | No | No | No | is not believed  to |

***Protocol Deviation Report***

| **Study ID** | **Event Name** | **Date of occurrence:** | **Description of occurrence** | **Compromised the scientific integrity of the data collected for the study?** | **Significant or substantive risk of harm to the research subject?** | **Willful or knowing breach of human subject protection regulations, policies, or procedures on the part of the investigator(s)?** | **Serious or continuing noncompliance with federal, state, local or institutional human subject protection regulations, policies, or procedures?** | **Inconsistent with the NIH**  **Human Research Protection Program? research, medical, and ethical principles?** | **Affects the rights, safety, or welfare of this subject or the integrity of research data (scientific soundness)?** |
| --- | --- | --- | --- | --- | --- | --- | --- | --- | --- |
| 200-010 | 24 Week Visit | 2018-12-02 | DXA scan could not be completed because patient has back issues and is unable to lay flat for the scan. | No | No | No | No | No | is not believed  to |
| 200-027 | Randomization | 2018-11-28 | DXA scan unable to be completed at randomization due to DXA machine being broke and needing a part. Will perform DXA at Week 4 visit. | No | No | No | No | No | is not believed  to |
| 100-025 | 4 Week Visit | 2018-11-08 | Unable to obtain a third blood pressure reading during the 4 week visit | No | No | No | No | No | is not believed  to |
| 100-025 | 4 Week Visit | 2018-11-08 | Subject lost bottles during her move. Was not able to return. | No | No | No | No | No | is not believed  to |
| 100-034 | 4 Week Visit | 2018-11-05 | Subject forgot to bring the medications back for this visit. | No | No | No | No | No | is not believed  to |
| 200-022 | Randomization | 2018-10-29 | Patient started cognitive testing and completed NIH Toolbox but got sick and could not complete the rest of the the cognitive testing at the visit. | No | No | No | No | No | is not believed  to |
| 200-004 | 16 Week Visit | 2018-10-08 | Patient arrived for Week 16 visit and reported that she forgot both her medication bottles from the last visit. Medications have never been returned. | No | No | No | No | No | is not believed  to |

***Protocol Deviation Report***

| **Study ID** | **Event Name** | **Date of occurrence:** | **Description of occurrence** | **Compromised the scientific integrity of the data collected for the study?** | **Significant or substantive risk of harm to the research subject?** | **Willful or knowing breach of human subject protection regulations, policies, or procedures on the part of the investigator(s)?** | **Serious or continuing noncompliance with federal, state, local or institutional human subject protection regulations, policies, or procedures?** | **Inconsistent with the NIH**  **Human Research Protection Program? research, medical, and ethical principles?** | **Affects the rights, safety, or welfare of this subject or the integrity of research data (scientific soundness)?** |
| --- | --- | --- | --- | --- | --- | --- | --- | --- | --- |
| 100-034 | Randomization | 2018-10-05 | IDS randomized the subject on Friday 10.5.18 for her randomization visit scheduled on Monday 10.8.18 before receiving confirmation from the study coordinator of the subject's arrival on the day of her visit. | No | No | No | No | No | is not believed  to |
| 100-010 | 24 Week Visit | 2018-10-01 | A discussion with the new monitor and the PI took place on 5/29/2020 that made this missing portion of the cognitive test a finding. All tests were complete except Pegboard & DSR. This subject had a hypoglycemic event occurred during the cognitive test at the 24W visit. | No | No | No | No | No | is not believed  to |
| 100-010 | 24 Week Visit | 2018-10-01 | A discussion with the new monitor and the PI took place on 5/29/2020 that made this missing portion of the OGTT test a finding. This subject had a hypoglycemic event occurred during the OGTT at the 24W visit. The 120 minute time point was not collected. | No | No | No | No | No | is not believed  to |
| 200-001 | 24 Week Visit | 2018-09-28 | Patient came in for Week 24 visit 1 day outside of visit windows. Patient could not come in within the windows because of her job. | No | No | No | No | No | is not believed  to |

***Protocol Deviation Report***

| **Study ID** | **Event Name** | **Date of occurrence:** | **Description of occurrence** | **Compromised the scientific integrity of the data collected for the study?** | **Significant or substantive risk of harm to the research subject?** | **Willful or knowing breach of human subject protection regulations, policies, or procedures on the part of the investigator(s)?** | **Serious or continuing noncompliance with federal, state, local or institutional human subject protection regulations, policies, or procedures?** | **Inconsistent with the NIH**  **Human Research Protection Program? research, medical, and ethical principles?** | **Affects the rights, safety, or welfare of this subject or the integrity of research data (scientific soundness)?** |
| --- | --- | --- | --- | --- | --- | --- | --- | --- | --- |
| 100-027 | 4 Week Visit | 2018-08-29 | Subject forgot to bring the medications back for this visit. | No | No | No | No | No | is not believed  to |
| 200-014 | 4 Week Visit | 2018-08-28 | During study coordinator review of patient's study logs at the week 4 visit she noted that patient did not step up with the metformin/placebo metformin according to study protocol.  Study coordinator made patient aware of mistake. Patient confirmed she made the mistake and did not pay attention to the document and directions from coordinator. | No | No | No | No | No | is not believed  to |
| 200-013 | Randomization | 2018-08-13 | Patient randomized incorrectly because metabolic syndrome was entered incorrectly into REDCap. Value in REDCap was entered as YES and should have been NO. | No | No | No | No | No | is not believed  to |
| 100-010 | 16 Week Visit | 2018-08-08 | Medications bottles not returned subject reports they were thrown out because they were in the clinical space at work and the Joint Commission was coming through. | No | No | No | No | No | is not believed  to |
| 100-004 | 20 Week PC | 2018-08-03 | medications not returned | No | No | No | No | No | is not believed  to |

***Protocol Deviation Report***

| **Study ID** | **Event Name** | **Date of occurrence:** | **Description of occurrence** | **Compromised the scientific integrity of the data collected for the study?** | **Significant or substantive risk of harm to the research subject?** | **Willful or knowing breach of human subject protection regulations, policies, or procedures on the part of the investigator(s)?** | **Serious or continuing noncompliance with federal, state, local or institutional human subject protection regulations, policies, or procedures?** | **Inconsistent with the NIH**  **Human Research Protection Program? research, medical, and ethical principles?** | **Affects the rights, safety, or welfare of this subject or the integrity of research data (scientific soundness)?** |
| --- | --- | --- | --- | --- | --- | --- | --- | --- | --- |
| 100-011 | 8 Week Visit | 2018-08-03 | medications not returned | No | No | No | No | No | is not believed  to |
| 200-008 | 8 Week Visit | 2018-07-24 | Patient arrived for Week 8 visit and reported that she forgot her OCP bottle from the last visit. Medication was never returned. | No | No | No | No | No | is not believed  to |
| 100-004 | 16 Week Visit | 2018-07-23 | subject forgot to bring medications to visit and never returned | No | No | No | No | No | is not believed  to |
| 200-010 | 4 Week Visit | 2018-07-12 | Patient arrived for Week 4 visit and reported that she forgot both her medication bottles from the last visit. Medications were never returned. | No | No | No | No | No | is not believed  to |

***Protocol Deviation Report***

| **Study ID** | **Event Name** | **Date of occurrence:** | **Description of occurrence** | **Compromised the scientific integrity of the data collected for the study?** | **Significant or substantive risk of harm to the research subject?** | **Willful or knowing breach of human subject protection regulations, policies, or procedures on the part of the investigator(s)?** | **Serious or continuing noncompliance with federal, state, local or institutional human subject protection regulations, policies, or procedures?** | **Inconsistent with the NIH**  **Human Research Protection Program? research, medical, and ethical principles?** | **Affects the rights, safety, or welfare of this subject or the integrity of research data (scientific soundness)?** |
| --- | --- | --- | --- | --- | --- | --- | --- | --- | --- |
| 100-019 | 4 Week Visit | 2018-07-09 | PD discovered on 6/25/19. Subject reports taking for 8 days (7/9/18 to 7/16/18) a total of 5 tablets which is 2500mg daily instead of 4 tablets which is 2000mg daily of the Metformin/Placebo tablets during the first 4 week period. Subject confirmed this dosage during study visit with CRC on 7/17/18. Originally dispensed 82 tablets total of Metformin/Placebo on 6/19/18. Subject returned 10 tablets on 7/17/18. Journals report taking 90 tablets in total when only 72 tablets were not returned, reporting a compliance of 87.80%. No AEs were ever reported from this patient. | No | No | No | No | No | is not believed  to |
| 200-012 | Randomization | 2018-06-27 | Patient randomized incorrectly because metabolic syndrome was entered incorrectly in REDCap. Value in REDCap was entered as YES and should have been NO. | No | No | No | No | No | is not believed  to |

***Protocol Deviation Report***

| **Study ID** | **Event Name** | **Date of occurrence:** | **Description of occurrence** | **Compromised the scientific integrity of the data collected for the study?** | **Significant or substantive risk of harm to the research subject?** | **Willful or knowing breach of human subject protection regulations, policies, or procedures on the part of the investigator(s)?** | **Serious or continuing noncompliance with federal, state, local or institutional human subject protection regulations, policies, or procedures?** | **Inconsistent with the NIH**  **Human Research Protection Program? research, medical, and ethical principles?** | **Affects the rights, safety, or welfare of this subject or the integrity of research data (scientific soundness)?** |
| --- | --- | --- | --- | --- | --- | --- | --- | --- | --- |
| 100-021 | Randomization | 2018-06-26 | A discussion with the new monitor and the PI took place on 5/29/2020 that made this missing portion of the cognitive test a finding. All tests were complete except the Visual puzzles at the ran visit. | No | No | No | No | No | is not believed  to |
| 200-008 | 4 Week Visit | 2018-06-21 | Patient arrived for Week 4 visit and reported that she forgot both her medication bottles from the last visit. Medications were never returned. | No | No | No | No | No | is not believed  to |
| 200-010 | Randomization | 2018-06-19 | DXA scan could not be completed because patient has back issues and is unable to lay flat for the scan. | No | No | No | No | No | is not believed  to |
| 100-011 | 4 Week Visit | 2018-05-25 | subject forgot to bring medications bottles | No | No | No | No | No | is not believed  to |
| 100-009 | 8 Week Visit | 2018-05-21 | Only the OCP bottle not returned | No | No | No | No | No | is not believed  to |

***Protocol Deviation Report***

| **Study ID** | **Event Name** | **Date of occurrence:** | **Description of occurrence** | **Compromised the scientific integrity of the data collected for the study?** | **Significant or substantive risk of harm to the research subject?** | **Willful or knowing breach of human subject protection regulations, policies, or procedures on the part of the investigator(s)?** | **Serious or continuing noncompliance with federal, state, local or institutional human subject protection regulations, policies, or procedures?** | **Inconsistent with the NIH**  **Human Research Protection Program? research, medical, and ethical principles?** | **Affects the rights, safety, or welfare of this subject or the integrity of research data (scientific soundness)?** |
| --- | --- | --- | --- | --- | --- | --- | --- | --- | --- |
| 100-010 | Randomization | 2018-04-16 | A discussion with the new monitor and the PI took place on 5/29/2020 that made this missing portion of the cognitive test a finding. At the Ran visit a portion of the Cognitive testing was not performed due to the subject falling asleep during the administration of the test due to sleep deprivation. This subject only completed nihtool box, rey both time points, and the memory stories both time points. | No | No | No | No | No | is not believed  to |
| 100-007 | Randomization | 2018-03-08 | A discussion with the new monitor and the PI took place on 5/29/2020 that made this missing portion of the cognitive test a finding. All tests were complete except Mental rotations due to patient sleep deprivation. | No | No | No | No | No | is not believed  to |
| 100-006 | Screening | 2018-02-19 | BP taken 3 minutes apart instead of 5 minutes apart by CRC Kieran Alessi. CRC Vresilovic noticed the mistake and retrained Kieran. | No | No | No | No | No | is not believed  to |
| 100-005 | Screening | 2018-02-16 | BP taken 1 minute apart instead of 5 minutes apart by CRC Kieran Alessi. CRC Vresilovic noticed the mistake and retrained Kieran. | No | No | No | No | No | is not believed  to |

***COVID-19 Protocol Deviations***

| **Study ID** | **Event Name** | **Date of occurrence:** | **Description of occurrence** |
| --- | --- | --- | --- |
| 100-112 | 4 Week Visit | 2020-11-22 | Remote visit due to COVID-19 |
| 100-116 | 4 Week Visit | 2020-11-20 | Remote visit due to COVID-19 |
| 100-102 | 8 Week Visit | 2020-11-19 | Remote visit due to COVID-19 |
| 100-113 | 8 Week Visit | 2020-11-19 | Remote visit due to COVID-19 |
| 100-120 | Screening | 2020-11-19 | Remote visit completed due to COVID-19 - Remote consent via REDCap, medical history, concomitant medications, and hirsutism. All other screening done in person |
| 100-119 | Screening | 2020-11-16 | Remote visit completed due to COVID-19 - Remote consent via REDCap, medical history, concomitant medications, and hirsutism. All other screening done in person |
| 100-099 | 8 Week Visit | 2020-11-11 | Remote visit due to COVID-19 |
| 100-107 | 4 Week Visit | 2020-11-11 | Remote visit due to COVID-19 |
| 100-114 | 4 Week Visit | 2020-11-06 | Remote visit due to COVID-19 |
| 100-108 | 8 Week Visit | 2020-10-29 | Remote visit due to COVID-19 |
| 100-111 | 8 Week Visit | 2020-10-29 | Remote visit due to COVID-19 |
| 100-109 | 8 Week Visit | 2020-10-26 | Remote visit due to COVID-19 |
| 100-102 | 4 Week Visit | 2020-10-22 | Remote visit due to COVID-19 |
| 100-118 | Screening | 2020-10-22 | Remote visit completed due to COVID-19 - Remote consent via REDCap, medical history, concomitant medications, and hirsutism. All other screening done in person |
| 100-113 | 4 Week Visit | 2020-10-20 | Remote visit due to COVID-19 |
| 100-101 | 8 Week Visit | 2020-10-19 | Remote visit due to COVID-19 |
| 100-099 | 4 Week Visit | 2020-10-15 | Remote visit completed due to COVID-19 |
| 100-109 | 4 Week Visit | 2020-09-30 | Remote visit completed due to COVID-19 |
| 100-111 | 4 Week Visit | 2020-09-29 | Remote visit completed due to COVID-19 |
| 100-108 | 4 Week Visit | 2020-09-28 | Remote visit completed due to COVID-19 |
| 100-104 | 8 Week Visit | 2020-09-23 | Remote visit due to COVID-19 |
| 100-106 | 8 Week Visit | 2020-09-23 | Remote visit due to COVID-19 |

***COVID-19 Protocol Deviations***

| **Study ID** | **Event Name** | **Date of occurrence:** | **Description of occurrence** |
| --- | --- | --- | --- |
| 100-116 | Screening | 2020-09-22 | Remote visit completed due to COVID-19 - Remote consent via REDCap, medical history, concomitant medications, and hirsutism. All other screening done in person |
| 100-117 | Screening | 2020-09-22 | Remote visit completed due to COVID-19 - Remote consent via REDCap, medical history, concomitant medications, and hirsutism. All other screening done in person |
| 100-115 | Screening | 2020-09-21 | Remote visit completed due to COVID-19 - Remote consent via REDCap, medical history, concomitant medications, and hirsutism. All other screening done in person |
| 100-101 | 4 Week Visit | 2020-09-18 | Remote visit due to COVID-19 |
| 100-100 | 8 Week Visit | 2020-09-15 | Remote visit due to COVID-19 |
| 100-105 | 8 Week Visit | 2020-09-14 | Remote visit conducted due to COVID-19 |
| 100-114 | Screening | 2020-09-08 | Remote visit completed due to COVID-19 - Remote consent via REDCap, medical history, concomitant medications, and hirsutism. All other screening done in person |
| 200-067 | 8 Week Visit | 2020-09-03 | Week 8 visit for this patient was conducted remotely because patient was experiencing fever symptoms. Study Coordinator had to follow institutions COVID visit policy. Unable to obtain puls measurements for this visit. |
| 100-113 | Screening | 2020-09-01 | Remote visit completed due to COVID-19 - Remote consent via REDCap, medical history, concomitant medications, and hirsutism. All other screening done in person |
| 100-104 | 4 Week Visit | 2020-08-25 | Remote visit due to COVID-19 |
| 100-106 | 4 Week Visit | 2020-08-24 | Remote visit due to COVID-19 |
| 100-094 | 8 Week Visit | 2020-08-21 | Remote visit due to COVID-19 |
| 100-100 | 4 Week Visit | 2020-08-17 | Remote visit for COVID-19 |

***COVID-19 Protocol Deviations***

| **Study ID** | **Event Name** | **Date of occurrence:** | **Description of occurrence** |
| --- | --- | --- | --- |
| 100-094 | 4 Week Visit | 2020-08-13 | 4W valid negative pregnancy test results sent 8/13/20. CRC sent UPT 3 times to subjects home on 7/27, 8/4, and 8/10. Subject stated not sexually active when sent all other 4W data on 7/26/20. |
| 100-093 | 8 Week Visit | 2020-08-07 | Remote visit for COVID-19 |
| 100-112 | Screening | 2020-08-07 | Remote visit completed due to COVID-19 - Remote consent via REDCap, medical history, concomitant medications, and hirsutism. All other screening done in person |
| 100-105 | 4 Week Visit | 2020-08-03 | Remote visit for COVID-19 |
| 100-111 | Screening | 2020-07-27 | Remote visit completed due to COVID-19 - Remote consent via REDCap, medical history, concomitant medications, and hirsutism. All other screening done in person |
| 100-094 | 4 Week Visit | 2020-07-24 | Remote visit due to COVID-19 |
| 100-110 | Screening | 2020-07-24 | Remote visit completed due to COVID-19 - Remote consent via REDCap, medical history, concomitant medications, and hirsutism. All other screening done in person |
| 100-109 | Screening | 2020-07-21 | Remote visit completed due to COVID-19 - Remote consent via REDCap, medical history, concomitant medications, and hirsutism. All other screening done in person |
| 100-108 | Screening | 2020-07-17 | Remote visit completed due to COVID-19 - Remote consent via REDCap, medical history, concomitant medications, and hirsutism. All other screening done in person |
| 100-107 | Screening | 2020-07-10 | Remote visit completed due to COVID-19 - Remote consent via REDCap, medical history, concomitant medications, and hirsutism. All other screening done in person |
| 100-093 | 4 Week Visit | 2020-07-09 | Remote visit due to COVID-19 |

***COVID-19 Protocol Deviations***

| **Study ID** | **Event Name** | **Date of occurrence:** | **Description of occurrence** |
| --- | --- | --- | --- |
| 100-106 | Screening | 2020-07-08 | Remote visit completed due to COVID-19 - Remote consent via REDCap, medical history, concomitant medications, and hirsutism. All other screening done in person |
| 100-105 | Screening | 2020-07-06 | Remote visit completed due to COVID-19 - Remote consent, medical history, concomitant medications, and hirsutism. All other screening done in person |
| 100-104 | Screening | 2020-06-30 | Remote visit completed due to COVID-19 - Remote consent, medical history, concomitant medications, and hirsutism. All other screening done in person |
| 100-102 | Screening | 2020-06-29 | Remote visit completed due to COVID-19 - Remote consent, medical history, concomitant medications, and hirsutism. All other screening done in person |
| 100-103 | Screening | 2020-06-29 | Remote visit completed due to COVID-19 - Remote consent, medical history, concomitant medications, and hirsutism. All other screening done in person |
| 100-101 | Screening | 2020-06-26 | Remote visit completed due to COVID-19 - Remote consent, medical history, concomitant medications, and hirsutism. All other screening done in person |
| 100-099 | Screening | 2020-06-25 | Remote visit completed due to COVID-19 - Remote consent, medical history, concomitant medications, and hirsutism. All other screening done in person |
| 100-100 | Screening | 2020-06-25 | Remote visit completed due to COVID-19 - Remote consent, medical history, concomitant medications, and hirsutism. All other screening done in person |
| 100-097 | Screening | 2020-06-16 | Remote visit completed due to COVID-19 - Remote consent, medical history, concomitant medications, and hirsutism. All other screening done in person |
| 100-098 | Screening | 2020-06-16 | Remote visit completed due to COVID-19 - Remote consent, medical history, concomitant medications, and hirsutism. All other screening done in person |

***COVID-19 Protocol Deviations***

| **Study ID** | **Event Name** | **Date of occurrence:** | **Description of occurrence** |
| --- | --- | --- | --- |
| 100-096 | Screening | 2020-06-15 | Remote consent, medical history, concomitant medications completed today due to  COVID-19 precautions. 100-096 sent hirsutism electronically. |
| 100-093 | Randomization | 2020-06-12 | randomization visit 3 days outside of screening window. IRB approved this exception on 6/5/20 due to COVID-19 restrictions. |
| 100-094 | Screening | 2020-06-05 | Remote consent, medical history assessment, concomitant medications, and hirsutism completed today due to  COVID-19 precautions |
| 100-095 | Screening | 2020-06-05 | Remote visit completed due to COVID-19 - Remote consent, medical history, concomitant medications, and hirsutism. All other screening done in person |
| 100-080 | 24 Week Visit | 2020-05-20 | Remote 24W visit due to  COVID-19 shutdown. No OGTT, fasting labs, TVUS, DXA collected. Cognitive test via telehealth. Subject counted pills at the end of the study. |
| 100-082 | 24 Week Visit | 2020-05-12 | Remote 24W visit due to  COVID-19 shutdown. No OGTT, fasting labs, TVUS, DXA collected. Cognitive test via telehealth. Subject counted pills at the end of the study. |
| 200-060 | 16 Week Visit | 2020-05-10 | Due to COVID 19 repository blood work was not obtained for Week 16 visit. |
| 100-092 | 8 Week Visit | 2020-05-06 | Remote visit due to COVID-19 |
| 100-079 | 24 Week Visit | 2020-05-05 | Remote 24W visit due to  COVID-19 shutdown. No OGTT, fasting labs, TVUS, DXA collected. Cognitive test via telehealth. Subject counted pills at the end of the study. |
| 100-078 | 24 Week Visit | 2020-05-04 | Remote visit due to COVID-19 shut down. No OGTT, fasting labs, TVUS, DXA collected.  Cognitive test via telehealth. 100-078 counted pills left at end of study |
| 100-089 | 8 Week Visit | 2020-05-04 | Remote visit for COVID-19 No scale at home so could not measure weight |

***COVID-19 Protocol Deviations***

| **Study ID** | **Event Name** | **Date of occurrence:** | **Description of occurrence** |
| --- | --- | --- | --- |
| 100-085 | 16 Week Visit | 2020-05-01 | Remote visit for COVID-19 No scale so could not measure weight |
| 200-063 | 8 Week Visit | 2020-04-22 | Week 8 visit did not take place because of COVID. Therefore, unable to obtain pulse measurements and collection of study medications. |
| 100-076 | 24 Week Visit | 2020-04-21 | Remote visit due to COVID-19 stay at home. No OGTT, fasting labs, TVUS, DXA collected.  Cognitive testing via telehealth. 100-076 counted pills left at end of study. |
| 100-087 | 8 Week Visit | 2020-04-21 | Remote visit completed due to COVID-19. self pill count conducted for this visit. |
| 200-064 | 4 Week Visit | 2020-04-13 | Due to COVID-29 pandemic Week 4 visit was performed remotely. Therefore, study medication was not collected. |
| 100-074 | 24 Week Visit | 2020-04-09 | Pill count documentation was not completed for this visit. It may be due to COVID-19 restrictions.  This finding was noted during the OCR Audit summer 2020 |
| 100-074 | 24 Week Visit | 2020-04-08 | Remote visit due to COVID-19. No OGTT, fasting labs, TVUS, DXA collected. Cognitive testing via telehealth. |
| 100-084 | 16 Week Visit | 2020-04-06 | Due to COVID-19 shutdown 16w appointment occurred remotely. Optional repository not collected. Safety labs done at LabCorp. |
| 100-092 | 4 Week Visit | 2020-04-06 | Remote visit completed due to COVID-19 |
| 100-089 | 4 Week Visit | 2020-03-31 | Remote visit due to COVID-19 No scale at home. Weight could not be completed |
| 200-055 | 24 Week Visit | 2020-03-26 | Due to COVID-19 issue the following for Week 24 Visit could not be performed: DXA scan, OGTT, UVA labs, TVU, collection of fasting labs, collection of study medications and portions of the cognitive and NIH Toolbox testing. Safety labs were drawn on 5/5/2020. |
| 100-080 | 16 Week Visit | 2020-03-23 | Remote COVID-19 visit. NO optional repository labs, Subject counted pills for this visit. |

***COVID-19 Protocol Deviations***

| **Study ID** | **Event Name** | **Date of occurrence:** | **Description of occurrence** |
| --- | --- | --- | --- |
| 200-057 | Early Termination Visit | 2020-03-23 | Due to COVID-19 patient withdrew from the study early and the following Early Termination procedures could not be completed: OGTT, cognitive testing and NIH Toolbox testing, safety labs, DXA scan, TVU, collection of medications and collection of fasting labs. Patient sent into active duty for COVID. |
| 200-057 | Early Termination Visit | 2020-03-23 | Due to COVID-19 patient withdrew from the study early. Study medication bottles were not returned and self report of pill count by patient was not completed. |
| 200-060 | 8 Week Visit | 2020-03-20 | Due to COVID-19 Week 8 visit completed remotely unable to obtain weight, height, waist and hip measurements, 3 blood pressures and collection of study medications. |
| 200-063 | 4 Week Visit | 2020-03-20 | Week 4 visit did not take place because of COVID-19. Therefore, unable to obtain waist and hip and pulse measurements and collection of study medication. |
| 100-087 | 4 Week Visit | 2020-03-19 | Remote COVID-19 visit |
| 100-087 | 4 Week Visit | 2020-03-19 | visit occurred during COVID-19 no self reported count. Subject returned all bottles to IDS at 24 Week visit and no IDS pill count either. |
| 100-082 | 16 Week Visit | 2020-03-18 | Remote COVID-19 visit. NO optional repository labs, Subject counted pills for this visit. |
| 100-075 | 24 Week Visit | 2020-03-05 | subject forgot to bring medications to last visit. Planned to bring to clinic but due to COVID-19 shutdown advised not to return. No self pill count. |
