# Supplementary material for: Impact of combined hormonal contraceptives and metformin on metabolic syndrome in women with hyperandrogenic polycystic ovary syndrome and obesity: The COMET-PCOS randomized clinical trial
Source: PLoS Med. 2025 Dec 8;22(12):e1004662. doi: 10.1371/journal.pmed.1004662 (PMC12697981; doi:10.1371/journal.pmed.1004662)
Supplement: S1 Table — (DOCX) [file pmed.1004662.s006.docx]

**S1. Table Eligibility Criteria**

**Inclusion Criteria**

| 1. | Women ≥ 18 to ≤ 40 years of age (at the time of screening), with hyperandrogenic PCOS. |
| --- | --- |
| 2. | Subjects will be diagnosed with PCOS defined by the most up to date Rotterdam criteria based on: a. Androgen excess AND  b. polycystic ovaries. Or  c. history of chronic anovulation or spontaneous periods. |
| 3. | BMI ≥ 25 kg/m² to ≤ 48 kg/m² obtained at screening visit. |
| 4. | In good general health according to the investigators discretion |
| 5. | Willing to avoid pregnancy for the duration of the study. |

**Exclusion Criteria**

| 1. | Current pregnancy or desire of pregnancy during course of study |
| --- | --- |
| 2. | Currently breastfeeding |
| 3. | Known 21 hydroxylase deficiency or any form of congential adrenal hyperplasia (CAH) |
| 4. | Untreated thyroid disease (TSH ≤0.45 mlU/mL and ≥ 4.5 mlU/mL) |
| 5. | Untreated hyperprolactinemia (2 Levels≥30 ng/ml at least one week apart) |
| 6. | Type 1 or type 2 Diabetes Mellitus currently receiving anti-diabetic agents, (subjects may wash out from metformin if taking the drug for another indication.) |
| 7. | Liver disease (AST/ALT≥2 times normal or a total bilirubin ≥2.5 mg/dL) |
| 8. | Renal disease (BUN≥30 mg/dL or serum creatinine ≥1.4 mg/dL) |
| 9. | Anemia (hemoglobin ≤10 mg/dL) |
| 10. | current history of alcohol abuse (≥ 22 drinks/week in the past 3 months) |
| 11. | Poorly controlled hypertension defined as average systolic blood pressure >= 150 mm Hg or average diastolic ≥100 mm Hg obtained on three measurements obtained 5 minutes apart. If treated, average systolic blood pressure ≥140 mm Hg or average diastolic ≥90 mm Hg |
| 12. | Patients with a history of, or suspected cervical carcinoma, endometrial carcinoma |
| 13. | TG≥250mg/dl |
| 14. | Current Use of lipid lowering or weight loss agents |
| 15. | Current use of hormonal contraceptives such as oral contraceptives, depo progestin, or hormonal implants |
| 16. | Participation in any study of an investigational drug or device or biological agent within 30 days |
| 17. | Suspected adrenal or ovarian tumor secreting androgens |
| 18. | Suspected Cushing’s syndrome |
| 19. | Bariatric surgery procedure in the recent past (≤12 months) |
| 20. | Absolute contraindications to the use of hormonal contraceptives or metformin (details in MOP) |
| 21. | Subjects who are unable to comply with the study procedures (In the opinion of the investigator) |
