# Supplementary material for: Impact of combined hormonal contraceptives and metformin on metabolic syndrome in women with hyperandrogenic polycystic ovary syndrome and obesity: The COMET-PCOS randomized clinical trial
Source: PLoS Med. 2025 Dec 8;22(12):e1004662. doi: 10.1371/journal.pmed.1004662 (PMC12697981; doi:10.1371/journal.pmed.1004662)
Supplement: S2 Table — (DOCX) [file pmed.1004662.s007.docx]

**S2 Table. Assessment of secondary outcomes**

| **Secondary End Points** | **Assessment at baseline and end of study** |
| --- | --- |
| Hyperandrogenism | Serum Total and free testosterone measured at Quest diagnostics, Ferriman-Gallwey Hirsutism Score [1] |
| Menstrual Frequency | Menstrual logs |
| Body Fat Distribution | Dual energy x-ray absorptiometry (DXA) using a  Hologic Discover (Hologic Inc) [2] |
| Lipoprotein | NMR spectroscopy measured at the NIH Clinical laboratory (Vantera, Labcorp, Burlington, NC, USA) [3] |
| Insulin Sensitivity | HOMA-IR and Matsuda’s Insulin Sensitivity Index [4] |
| Glucose homeostasis | 75gram 2-hour oral Glucose tolerance test (oGTT), [5] glucose and insulin measured at the translational Core Laboratories, University of Pennsylvania |
| Health related quality of life | PCOSQ [6] validated for women with PCOS and includes  five domains: emotional, body hair, infertility, weight,  and menstrual problems |
| Nutritional Intake | Automated Self-Administered 24-hour dietary (ASA 24) [7] recall Healthy Eating Index (HEI) was assessed at 12 and 24 weeks |

1. Yildiz BO, Bolour S, Woods K, et al. Visually scoring hirsutism. Hum Reprod Update. 2010; 16:51–64.

2. Micklesfield LK, Goedecke JH, Punyanitya M, Wilson KE, Kelly TL. Dual-energy X-ray performs as well as clinical computed tomography for the measurement of visceral fat. Obesity. 2012; 20:1109–1114

3. Dokras A, Playford M, Kris-Etherton PM, Kunselman AR, Stetter CM, Williams NI, Gnatuk CL, Estes SJ, Sarwer DB, Allison KC, Coutifaris C, Mehta N, Legro RS. Impact of hormonal contraception and weight loss on high-density lipoprotein cholesterol efflux and lipoprotein particles in women with polycystic ovary syndrome Clin Endocrinol (Oxf). 2017 May;86(5):739-746

4.Matsuda M, DeFronzo RA. Insulin sensitivity indices obtained from oral glucose tolerance testing: Comparison with the euglycemic insulin clamp. Diabetes Care. 1999; 22:1462–1470.

5. D.I. Phillips, P.M. Clark, C.N. Hales, C. Osmond Understanding oral glucose tolerance: comparison of glucose or insulin measurements during the oral glucose tolerance test with specific measurements of insulin resistance and insulin secretion Diabet Med, 11 (1994), pp. 286–292.

6. Guyatt G, Weaver B, Cronin L, Dooley JA, Azziz R. Health-related quality of life in women with polycystic ovary syndrome, a self-administered questionnaire. J Clin Epidemiol. 2004;57(12):1279-1287.

7. Subar AF, Kirkpatrick SI, Mittl B, et al. The Automated Self-Administered 24-hour dietary recall (ASA24): a resource for researchers, clinicians, and educators from the National Cancer Institute. J Acad Nutr Diet. 2012;112(8):1134-1137.
