# Supplementary material for: Impact of combined hormonal contraceptives and metformin on metabolic syndrome in women with hyperandrogenic polycystic ovary syndrome and obesity: The COMET-PCOS randomized clinical trial
Source: PLoS Med. 2025 Dec 8;22(12):e1004662. doi: 10.1371/journal.pmed.1004662 (PMC12697981; doi:10.1371/journal.pmed.1004662)
Supplement: S3 Table — (DOCX) [file pmed.1004662.s008.docx]

**S3 table Baseline Characteristics of Participants related to additional secondary outcomes**

|  | **COCP (N=79)** | **Metformin (N=81)** | **Combined (N=80)** |  |
| --- | --- | --- | --- | --- |
| ^a^ **PCOSQOL and mental health surveys** | | | |  |
| PCOSQ: Emotion domain mean (SD) | 3.9 (1.4) | 3.9 (1.3) | 3.8 (1.4) |  |
| PCOSQ: Body Hair domain mean (SD) | 3.6 (1.8) | 3.8 (1.6) | 3.6 (1.6) |  |
| PCOSQ: Weight domain mean (SD) | 2.3 (1.4) | 2.2 (1.3) | 2.1 (1.2) |  |
| PCOSQ: Infertility domain mean (SD) | 4.1 (1.9) | 4.0 (1.8) | 4.0 (1.9) |  |
| PCOSQ: Menstrual domain mean (SD) | 3.9 (1.2) | 3.8 (1.4) | 3.3 (1.3) |  |
| PCOSQ: Acne domain mean (SD) | 5.3 (1.6) | 4.9 (1.6) | 4.8 (1.5) |  |
| ^b^ CES-D Total Score mean (SD) | 17.3 (11.2) | 19.1 (12.4) | 16.6 (11.8) |  |
| ^c^**Lipoprotein Analysis** | | | |  |
| Total TRLP mean (SD) nmol/L | 120.5 (62.1) | 96.0 (47.8) | 116.8 (67.4) |  |
| *Very Large TRLP* mean (SD) *nmol/L* | 0.2 (0.2) | 0.1 (0.2) | 0.1 (0.2) |  |
| *Large TRLP* mean (SD) *nmol/L* | 2.8 (3.9) | 1.8 (2.9) | 2.6 (4.0)] |  |
| *Medium TRLP* mean (SD*) nmol/L* | 15.4 (14.1) | 10.8 (12.1) | 11.5 (11.1) |  |
| *Small TRLP* mean (SD) *nmol/L* | 54.5 (38.5) | 50.2 (35.4) | 54.3 (35.7) |  |
| *Very Small TRLP* mean (SD) *nmol/L* | 47.6 (47.3) | 33.0 (30.3) | 48.2 (45.0) |  |
| TRL Triglycerides median (IQR) mg/dL | | 67.0 (37.0, 98.0) | 52.0 (26.5, 80.5) | 54.0 (35.0, 85.0) |
| TRL Cholesterol median (IQR) mg/dL | | 18.0 (13.0, 23.0) | 16.0 (11.0, 19.5) | 16.0 (12.0, 22.0) |
| Total LDLP mean (SD) nmol/L | 1310.4 (317.9) | 1343.3 (369.7) | 1334.4 (327.3) |  |
| *Large LDLP* mean (SD) *nmol/L* | 359.2 (194.4) | 357.1 (217.3) | 344.2 (190.0) |  |
| *Medium LDLP* mean (SD) *nmol/L* | 468.2 (251.9) | 508.7 (312.6) | 413.7 (258.0) |  |
| *Small LDLP* mean (SD) *nmol/L* | 483.1 (379.3) | 477.6 (312.5) | 576.6 (362.5) |  |
| LDL Size mean (SD) nm | 21.2 (0.4) | 21.2 (0.4) | 21.1 (0.4) |  |
| LDL mean (SD) mg/dL | 107.8 (27.1) | 107.5 (28.3) | 108.4 (25.1) |  |
| Total HDLP mean (SD) µmol/L | 19.0 (2.5) | 18.6 (2.6) | 18.5 (2.3) |  |
| *Large HDLP* mean (SD) *µmol/L* | 1.7 (0.9) | 1.9 (1.4) | 1.8 (1.0) |  |
| *Medium HDLP* mean (SD) *µmol/L* | 4.3 (1.9) | 4.1 (2.2) | 3.9 (1.6) |  |
| *Small HDLP* mean (SD) *µmol/L* | 13.0 (2.6) | 12.5 (3.2) | 12.9 (2.6) |  |
| HDL mean (SD) mg/dL | 49.5 (10.9) | 50.0 (11.6) | 48.4 (10.6) |  |
| HDL Size mean (SD) nm | 9.0 (0.3) | 9.0 (0.4) | 8.9 (0.3) |  |
| Triglycerides median (IQR) mg/dL | 96.5 (65.0, 129.0) | 85.5 (55.0, 107.0) | 85.0 (64.0, 122.0) |  |
| Total Cholesterol mean (SD) mg/dL | 176.6 (31.8) | 174.5 (32.5) | 175.0 (27.5) |  |

^a^PCOSQ domain scores are graded on a scale of 1 (poorest function) to 7 (optimal function)

^b^The Center for Epidemiologic Studies Depression Scale (CES-D) with higher scores indicating more severe depressive symptoms. 2 participants in the COCP group and 1 participant in the combined group were missing total score.

^c^Lipoprotein analysis data were missing in 1 participant in the COCP group, 2 participants in the metformin group and 2 participants in the combined group
