# Supplementary material for: Impact of combined hormonal contraceptives and metformin on metabolic syndrome in women with hyperandrogenic polycystic ovary syndrome and obesity: The COMET-PCOS randomized clinical trial
Source: PLoS Med. 2025 Dec 8;22(12):e1004662. doi: 10.1371/journal.pmed.1004662 (PMC12697981; doi:10.1371/journal.pmed.1004662)
Supplement: S4 Table — (DOCX) [file pmed.1004662.s009.docx]

**Table S4. Change in Prevalence of Metabolic Syndrome (MetS) and its Components between Baseline (Pre) and End of Study Visits (Post) (unadjusted)**

|  | **COCP**  **Post vs. Pre** | | **Metformin**  **Post vs. Pre** | | **Combined**  **Post vs. Pre** | | **^a^COCP**  **vs. Metformin** | **^a^COCP**  **vs. Combined** | **^a^Metformin**  **vs. Combined** |
| --- | --- | --- | --- | --- | --- | --- | --- | --- | --- |
|  | **OR**  **(95% CI)** | **P-**  **value** | **OR**  **(95% CI)** | **P-**  **value** | **OR**  **(95% CI)** | **P-**  **value** | **P-**  **value** | **P-**  **value** | **P-**  **value** |
| **Metabolic Syndrome** | 1.05 (0.68, 1.62) | 0.83 | 0.80 (0.45, 1.39) | 0.42 | 0.84 (0.48, 1.47) | 0.53 | 0.45 | 0.54 | 0.90 |
| **MetS abnormal criterion**  **definition** |  |  |  |  |  |  |  |  |  |
| Waist circumference  ≥88cm (≥80cm for Asian subjects) | 0.39 (0.15, 1.00) | 0.05 | 0.46 (0.21, 1.03) | 0.06 | 1.01 (0.43, 2.37) | 0.99 | 0.79 | 0.15 | 0.19 |
| Triglyceride  ≥150mg/dl | 2.31 (1.34, 3.98) | 0.003 | 1.27 (0.70, 2.30) | 0.44 | 1.76 (0.99, 3.12) | 0.05 | 0.14 | 0.50 | 0.43 |
| HDL <50mg/dl | 0.74 (0.48, 1.12) | 0.15 | 0.81 (0.50, 1.31) | 0.39 | 0.62 (0.39, 0.96) | 0.03 | 0.78 | 0.57 | 0.42 |
| Blood Pressure  ≥130/85mmHg or use of anti-hypertensive medications | 1.00 (0.56, 1.78) | 1.00 | 0.41 (0.22, 0.77) | 0.01 | 0.89 (0.48, 1.66) | 0.72 | 0.04 | 0.80 | 0.08 |
| ^b^Fasting Glucose ≥100mg/dl | 1.85 (0.92, 3.74) | 0.09 | 1.72 (0.97, 3.06) | 0.07 | 0.61 (0.24, 1.54) | 0.30 | 0.87 | 0.06 | 0.06 |

Odds ratios were obtained via binary logistic regression using GEE and calculated as [odds at end of study] / [odds at baseline].

^a^ comparison of the ORs between groups.

^b^Type 1 and 2 diabetes was an exclusion criterion
