# Supplementary material for: Impact of combined hormonal contraceptives and metformin on metabolic syndrome in women with hyperandrogenic polycystic ovary syndrome and obesity: The COMET-PCOS randomized clinical trial
Source: PLoS Med. 2025 Dec 8;22(12):e1004662. doi: 10.1371/journal.pmed.1004662 (PMC12697981; doi:10.1371/journal.pmed.1004662)
Supplement: S5 Table — (DOCX) [file pmed.1004662.s010.docx]

**S5 Table. Change in Secondary Outcomes from Baseline to End of Study Visits within each group and comparison between OCP, metformin and combined groups (unadjusted)**

|  | **COCP**  **(n=79)** | | **Metformin**  **(n=81)** | | **Combined**  **(n-80)** | | **COCP**  **vs. Metformin** | **COCP**  **vs. Combined** | **Metformin vs. Combined** |
| --- | --- | --- | --- | --- | --- | --- | --- | --- | --- |
|  | **Change from Baseline**  **(95% CI)^a^** | **P-value** | **Change from Baseline**  **(95% CI) ^a^** | **P-value** | **Change from Baseline**  **(95% CI) ^a^** | **P-value** | **P-value** | **P-value** | **P-value** |
| **Biometric parameters** | | | | | | | | | |
| Weight (kg) | -1.40 (-3.03, 0.23) | 0.09 | -0.24 (-1.77, 1.28) | 0.75 | -1.30 (-2.85, 0.25) | 0.10 | 0.31 | 0.93 | 0.34 |
| BMI (kg/m^2^) | -0.49 (-0.88, -0.10) | 0.01 | -0.09 (-0.46, 0.29) | 0.65 | -0.47 (-0.85, -0.09) | 0.02 | 0.14 | 0.94 | 0.16 |
| Waist circumference (cm) | -2.27 (-4.03, -0.51) | 0.01 | -1.65 (-3.33, 0.02) | 0.05 | -2.42 (-4.11, -0.72) | 0.006 | 0.61 | 0.91 | 0.53 |
| Systolic BP  (mmHg) | 2.60 (0.44, 4.77) | 0.02 | 0.65 (-1.42, 2.71) | 0.54 | 3.25 (1.16, 5.35) | 0.003 | 0.20 | 0.67 | 0.08 |
| Diastolic BP (mmHg) | 1.87 (0.40, 3.35) | 0.01 | 0.16 (-1.24, 1.57) | 0.82 | 1.53 (0.10, 2.95) | 0.04 | 0.10 | 0.74 | 0.18 |
| **PCOS diagnosis parameters** | | | | | | | | | |
| Total  Testosterone ^b^ | 0.63 (0.55, 0.71) | <.001 | 0.88 (0.77, 0.99) | 0.03 | 0.68 (0.60, 0.77) | <.001 | <.001 | 0.35 | 0.006 |
| Free  Testosterone ^b^ | 0.28 (0.23, 0.35) | <.001 | 0.81 (0.66, 0.99) | 0.04 | 0.36 (0.30, 0.45) | <.001 | <.001 | 0.09 | <.001 |
| SHBG ^b^ | 3.38 (2.92, 3.92) | <.001 | 1.07 (0.93, 1.23) | 0.33 | 2.69 (2.33, 3.11) | <.001 | <.001 | 0.03 | <.001 |
| Ferriman-Gallwey Score | -1.76 (-2.82, -0.70) | 0.001 | -1.60 (-2.60, -0.60) | 0.002 | -2.82 (-3.82, -1.81) | <.001 | 0.83 | 0.15 | 0.09 |
| AMH ^b^ | 0.69 (0.63, 0.75) | <.001 | 1.01 (0.93, 1.10) | 0.77 | 0.76 (0.69, 0.83) | <.001 | <.001 | 0.14 | <.001 |
| Antral Follicle Count ^b^ | 0.77 (0.68, 0.88) | <.001 | 1.05 (0.93, 1.19) | 0.45 | 0.75 (0.67, 0.85) | <.001 | 0.001 | 0.77 | <.001 |
| Total Ovarian Volume ^b^ | 0.61 (0.52, 0.71) | <.001 | 1.00 (0.87, 1.16) | 0.95 | 0.69 (0.60, 0.80) | <.001 | <.001 | 0.21 | <.001 |
| **Oral Glucose Tolerance Test (oGTT)** | | | | | | | | | |
| Fasting Glucose ^b^ | 0.97 (0.94, 1.00) | 0.03 | 0.99 (0.97, 1.02) | 0.52 | 0.97 (0.95, 1.00) | 0.04 | 0.24 | 0.82 | 0.33 |
| 2-hour Glucose ^b^ | 1.13 (1.05, 1.22) | 0.001 | 1.00 (0.93, 1.07) | 0.89 | 1.15 (1.07, 1.23) | <.001 | 0.02 | 0.75 | 0.005 |
| AUC Glucose ^b^ | 1.08 (1.02, 1.13) | 0.005 | 1.00 (0.95, 1.05) | 1.00 | 1.04 (0.99, 1.09) | 0.15 | 0.04 | 0.28 | 0.31 |
| Fasting Insulin ^b^ | 1.02 (0.87, 1.20) | 0.78 | 1.13 (0.97, 1.32) | 0.12 | 1.03 (0.88, 1.20) | 0.75 | 0.38 | 0.98 | 0.39 |
| 2-hour Insulin ^b^ | 1.09 (0.89, 1.32) | 0.40 | 0.84 (0.70, 1.02) | 0.08 | 1.18 (0.98, 1.42) | 0.09 | 0.07 | 0.56 | 0.01 |
| AUC Insulin ^b^ | 1.07 (0.95, 1.20) | 0.24 | 0.93 (0.83, 1.04) | 0.18 | 1.03 (0.92, 1.15) | 0.60 | 0.08 | 0.62 | 0.19 |
| Matsuda's Insulin Sensitivity Index ^b^ | 0.94 (0.83, 1.07) | 0.36 | 1.01 (0.90, 1.14) | 0.86 | 0.93 (0.82, 1.05) | 0.23 | 0.43 | 0.87 | 0.33 |
| HOMA-IR ^b^ | 1.03 (0.88, 1.21) | 0.69 | 1.05 (0.90, 1.22) | 0.53 | 1.02 (0.87, 1.18) | 0.84 | 0.88 | 0.88 | 0.76 |
| **DXA parameters** | | | | | | | | | |
| Fat mass (kg) | -0.83 (-1.65, -0.01) | 0.05 | -0.49 (-1.25, 0.27) | 0.21 | 0.06 (-0.70, 0.82) | 0.87 | 0.55 | 0.12 | 0.31 |
| Lean mass (kg) | -0.93 (-1.44, -0.41) | <.001 | -0.07 (-0.54, 0.41) | 0.79 | -1.24 (-1.71, -0.76) | <.001 | 0.02 | 0.39 | <.001 |
| Total Mass (kg) (Fat+Lean) | -1.75 (-2.79, -0.70) | 0.001 | -0.55 (-1.52, 0.41) | 0.26 | -1.17 (-2.14, -0.20) | 0.02 | 0.10 | 0.42 | 0.37 |
| % Fat | 0.01 (-0.52, 0.54) | 0.97 | -0.29 (-0.78, 0.20) | 0.24 | 0.55 (0.06, 1.04) | 0.03 | 0.41 | 0.14 | 0.02 |
| Android Fat (g) | -168 (-265, -71) | <.001 | -87 (-177, 3) | 0.06 | -58 (-148, 32) | 0.20 | 0.23 | 0.10 | 0.65 |
| Gynoid Fat (g) | -129 (-268, 10) | 0.07 | -43 (-172, 86) | 0.51 | -5 (-134, 124) | 0.94 | 0.37 | 0.20 | 0.68 |
| Android/  Gynoid fat ratio | -0.013 (-0.024, -0.003) | 0.010 | -0.008 (-0.018, 0.001) | 0.09 | -0.007 (-0.017, 0.002) | 0.13 | 0.46 | 0.38 | 0.88 |

^a^ change based on estimated marginal means
^b^ data were log-transformed for analysis and changes reported as ratio of geometric marginal means
