# Supplementary material for: Impact of combined hormonal contraceptives and metformin on metabolic syndrome in women with hyperandrogenic polycystic ovary syndrome and obesity: The COMET-PCOS randomized clinical trial
Source: PLoS Med. 2025 Dec 8;22(12):e1004662. doi: 10.1371/journal.pmed.1004662 (PMC12697981; doi:10.1371/journal.pmed.1004662)
Supplement: S6 Table — (A) Dietary intake based on ASA-24 data collected at week 12 and end of study Visit. (B) shows unadjusted values. (DOCX) [file pmed.1004662.s011.docx]

**Table S6A. Dietary intake based on Automated Self-Administered 24-hour dietary recall**

**(ASA-24) data collected at week 12 and end of study visit**

|  | COCP | Metformin | Combined | OCP vs Metformin | OCP vs combined | Metformin vs Combined |
| --- | --- | --- | --- | --- | --- | --- |
|  | *Marginal mean (95%CI) | *Marginal mean (95%CI) | *Marginal mean (95%CI) | P-value | P-value | P-value |
| Energy (Kcal) |  |  |  |  |  |  |
| Week 12 | 1903.8  (1728.4, 2079.2) | 1716.0  (1551.2, 1880.8) | 1573.9  (1405.5, 1742.3) | 0.08 | 0.003 | 0.19 |
| End of study visit | 1865.3  (1663.7, 2066.9) | 1670.8  (1484.7, 1857.0) | 1484.7  (1304.0, 1665.3) | 0.13 | 0.003 | 0.13 |
| Protein (g) |  |  |  |  |  |  |
| Week 12 | 89.4 (80.2, 98.6) | 71.7 (63.0, 80.3) | 64.8 (55.9, 73.6) | 0.002 | <.0001 | 0.23 |
| End of study visit | 84.9 (74.3, 95.4) | 70.9 (61.2, 80.7) | 62.3 (52.8, 71.7) | 0.04 | <0.001 | 0.18 |
| Total Fat (g) |  |  |  |  |  |  |
| Week 12 | 82.8 (73.1, 92.5) | 67.0 (57.9, 76.1) | 63.1 (53.8, 72.4) | 0.008 | 0.001 | 0.51 |
| End of study visit | 79.7 (67.8, 91.6) | 68.3 (57.3, 79.3) | 61.3 (50.6, 72.0) | 0.13 | 0.02 | 0.34 |
| Carbohydrate (g) |  |  |  |  |  |  |
| Week 12 | 198.6 (177.2, 220.1) | 197.5 (177.3, 217.7) | 182.5 (161.8, 203.2) | 0.93 | 0.24 | 0.26 |
| End of study Visit | 198.8 (175.2, 222.3) | 189.9 (168.2, 211.6) | 170.6 (149.5, 191.6) | 0.55 | 0.06 | 0.17 |
| Total Healthy Eating Index (HEI) score |  |  |  |  |  |  |
| Week 12 | 52.7 (48.5, 57.0) | 53.9 (49.9, 57.8) | 48.8 (44.7, 52.8) | 0.67 | 0.14 | 0.05 |
| End of study Visit | 53.3 (48.8, 57.9) | 53.8 (49.7, 58.0) | 49.9 (45.8, 53.9) | 0.85 | 0.22 | 0.14 |

* estimated marginal means adjusted for the randomization stratification factors of site, race, and metabolic syndrome.

**UNADJUSTED Table S6B. Dietary intake based on ASA-24 data collected at week 12 and end of study visit**

|  | COCP | Metformin | Combined | OCP vs Metformin | OCP vs combined | Metformin vs Combined |
| --- | --- | --- | --- | --- | --- | --- |
|  | Marginal mean (95%CI) | Marginal mean (95%CI) | Marginal mean (95%CI) | P-value | P-value | P-value |
| Energy (Kcal) |  |  |  |  |  |  |
| Week 12 | 1811.9 (1656.0, 1967.7) | 1625.1 (1477.8, 1772.5) | 1490.5 (1336.3, 1644.6) | 0.09 | 0.004 | 0.21 |
| End of study visit | 1775.6 (1590.3, 1960.8) | 1583.1 (1411.4, 1754.7) | 1408.0 (1238.7, 1577.2) | 0.13 | 0.004 | 0.15 |
| Protein (g) |  |  |  |  |  |  |
| Week 12 | 87.3 (79.2, 95.4) | 69.7 (62.0, 77.4) | 63.1 (55.0, 71.1) | 0.002 | <.0001 | 0.24 |
| End of study visit | 82.8 (73.1, 92.4) | 69.0 (60.1, 78.0) | 60.7 (51.9, 69.5) | 0.04 | 0.001 | 0.19 |
| Total Fat (g) |  |  |  |  |  |  |
| Week 12 | 80.4 (71.8, 88.9) | 64.2 (56.1, 72.3) | 60.7 (52.2, 69.1) | 0.007 | 0.001 | 0.55 |
| End of study visit | 77.2 (66.4, 88.1) | 65.7 (55.6, 75.8) | 59.1 (49.2, 69.1) | 0.13 | 0.02 | 0.36 |
| Carbohydrate (g) |  |  |  |  |  |  |
| Week 12 | 185.1 (165.8, 204.3) | 184.6 (166.3, 202.8) | 170.2 (151.1, 189.3) | 0.97 | 0.28 | 0.28 |
| End of study Visit | 185.4 (163.6, 207.1) | 177.4 (157.3, 197.5) | 159.3 (139.4, 179.1) | 0.60 | 0.08 | 0.21 |
| Total Healthy Eating Index (HEI) score |  |  |  |  |  |  |
| Week 12 | 52.7 (48.9, 56.5) | 53.7 (50.1, 57.3) | 49.0 (45.3, 52.8) | 0.69 | 0.18 | 0.08 |
| End of study Visit | 53.3 (49.2, 57.4) | 53.7 (49.9, 57.5) | 50.0 (46.3, 53.8) | 0.88 | 0.25 | 0.18 |

* Automated Self-Administered 24-hour dietary recall

*****
