# Supplementary material for: Impact of combined hormonal contraceptives and metformin on metabolic syndrome in women with hyperandrogenic polycystic ovary syndrome and obesity: The COMET-PCOS randomized clinical trial
Source: PLoS Med. 2025 Dec 8;22(12):e1004662. doi: 10.1371/journal.pmed.1004662 (PMC12697981; doi:10.1371/journal.pmed.1004662)
Supplement: S7 Table — (A) Change in additional secondary outcomes from baseline to end of study within each group and comparison between OCP, metformin, and combined groups. (B) shows unadjusted values. (DOCX) [file pmed.1004662.s012.docx]

**Table S7A. Change in additional secondary outcomes from baseline to end of study within each group and comparison between OCP, metformin and combined groups**

|  | **COCP**  **N=79** | | | | **Metformin**  **N=81** | | | **Combined**  **n-80** | | | **^c^COCP**  **vs. Metformin** | | **^c^COCP**  **vs. Combined** | | **^c^Metformin vs. Combined** | |
| --- | --- | --- | --- | --- | --- | --- | --- | --- | --- | --- | --- | --- | --- | --- | --- | --- |
|  | **Change from Baseline**  **(95% CI)^a^** | | **P-value** | | **Change from Baseline**  **(95% CI) ^a^** | **P-value** | | **Change from Baseline**  **(95% CI) ^a^** | **P-value** | | **P-value** | | **P-value** | | **P-value** | |
| **PCOSQ and mental health surveys** | | | | | | | | | | | | | | | | |
| Emotion  domain | 0.39  (0.12, 0.67) | | 0.005 | | 0.40  (0.14, 0.66) | 0.003 | | 0.44  (0.17, 0.70) | 0.001 | | 0.96 | | 0.81 | | 0.84 | |
| Body Hair  domain | 0.74  (0.46, 1.02) | | <.001 | | 0.26  (-0.01, 0.53) | 0.06 | | 0.62  (0.35, 0.90) | <.001 | | 0.02 | | 0.56 | | 0.06 | |
| Weight domain | 0.72  (0.37, 1.07) | | <.001 | | 0.34  (0.01, 0.68) | 0.05 | | 0.85  (0.51, 1.19) | <.001 | | 0.13 | | 0.60 | | 0.04 | |
| Infertility  domain | 0.55  (0.21, 0.88) | | 0.001 | | 0.48  (0.16, 0.80) | 0.004 | | 0.48  (0.16, 0.81) | 0.003 | | 0.76 | | 0.79 | | 0.97 | |
| Menstrual  Problem  domain | 0.28  (-0.03, 0.59) | | 0.07 | | 0.45  (0.16, 0.75) | 0.003 | | 0.67  (0.37, 0.97) | <.001 | | 0.43 | | 0.07 | | 0.31 | |
| Acne  domain | 0.37  (0.08, 0.66) | | 0.01 | | 0.40  (0.12, 0.68) | 0.006 | | 0.78  (0.50, 1.07) | <.001 | | 0.89 | | 0.05 | | 0.06 | |
| CES-D Total Score | -1.93  (-4.47, 0.61) | | 0.14 | | -6.13  (-8.55, -3.72) | <.001 | | -0.47  (-2.92, 1.98) | 0.71 | | 0.02 | | 0.42 | | 0.001 | |
| **Lipoprotein Analysis** | | | | | | | | | | | | | | | | |
| Total TRLP (nmol/L) | -2.26  (-14.71, 10.19) | 0.72 | | 6.33  (-5.35, 18.02) | | 0.29 | 10.24  (-1.70, 22.18) | | 0.09 | 0.32 | | 0.15 | | 0.65 | |  |
| *Very Large TRLP*  *(nmol/L)* | -0.01  (-0.12, 0.10) | 0.85 | | -0.02  (-0.12, 0.08) | | 0.73 | 0.14  (0.04, 0.24) | | 0.009 | 0.92 | | 0.05 | | 0.03 | |  |
| *Large TRLP (nmol/L)* | 0.80  (-0.25, 1.86) | 0.13 | | 0.88  (-0.11, 1.87) | | 0.08 | 2.12  (1.11, 3.13) | | <.001 | 0.92 | | 0.08 | | 0.09 | |  |
| *Medium TRLP (nmol/L)* | 0.90  (-2.50, 4.31) | 0.60 | | 4.00  (0.79, 7.20) | | 0.01 | 4.71  (1.44, 7.98) | | 0.005 | 0.19 | | 0.11 | | 0.76 | |  |
| *Small TRLP (nmol/L)* | -18.22  (-27.06, -9.37) | <.001 | | -1.96  (-10.36, 6.44) | | 0.65 | -16.21  (-24.79, -7.64) | | <.001 | 0.009 | | 0.75 | | 0.02 | |  |
| *Very Small TRLP*  *(nmol/L)* | 15.66  (3.34, 27.97) | 0.01 | | 3.05  (-8.54, 14.65) | | 0.60 | 19.14  (7.32, 30.97) | | 0.002 | 0.14 | | 0.69 | | 0.06 | |  |
| **^b^**  TRL Triglycerides | 1.07  (0.93, 1.22) | 0.35 | | 1.16  (1.02, 1.31) | | 0.03 | 1.20  (1.05, 1.37) | | 0.007 | 0.40 | | 0.22 | | 0.68 | |  |
| **^b^**  TRL Cholesterol | 1.22  (1.12, 1.32) | <.001 | | 1.11  (1.03, 1.20) | | 0.01 | 1.31  (1.21, 1.42) | | <.001 | 0.11 | | 0.23 | | 0.004 | |  |
| Total LDLP (nmol/L) | 150.03  (90.11, 209.95) | <.001 | | -6.16  (-62.49, 50.17) | | 0.83 | 136.20  (78.59, 193.81) | | <.001 | <.001 | | 0.74 | | <.001 | |  |
| *Large LDLP (nmol/L)* | -69.25  (-112.6, -25.88) | 0.002 | | -57.61  (-98.69, -16.52) | | 0.006 | -41.40  (-83.37, 0.57) | | 0.05 | 0.70 | | 0.36 | | 0.59 | |  |
| *Medium LDLP (nmol/L)* | 66.17  (-11.20, 143.55) | 0.09 | | -5.43  (-78.93, 68.06) | | 0.88 | 61.10  (-13.87, 136.07) | | 0.11 | 0.19 | | 0.93 | | 0.21 | |  |
| *Small LDLP (nmol/L)* | 168.50  (85.58, 251.41) | <.001 | | 58.70  (-19.57, 136.96) | | 0.14 | 115.37  (35.48, 195.26) | | 0.005 | 0.06 | | 0.36 | | 0.32 | |  |
| LDL Size (nm) | -0.19  (-0.28, -0.10) | <.001 | | -0.11  (-0.19, -0.03) | | 0.007 | -0.14  (-0.22, -0.06) | | <.001 | 0.19 | | 0.41 | | 0.62 | |  |
| LDL-cholesterol (mg/dL) | -6.70  (-11.19, -2.20) | 0.004 | | -3.24  (-7.48, 1.01) | | 0.13 | -3.99  (-8.33, 0.34) | | 0.07 | 0.27 | | 0.39 | | 0.81 | |  |
| Total HDLP (µmol/L) | 1.79  (1.15, 2.43) | <.001 | | 0.60  (-0.00, 1.20) | | 0.05 | 2.23  (1.62, 2.85) | | <.001 | 0.008 | | 0.32 | | <.001 | |  |
| *Large HDLP (µmol/L)* | 0.36  (0.12, 0.60) | 0.003 | | 0.14  (-0.08, 0.36) | | 0.21 | 0.02  (-0.21, 0.25) | | 0.85 | 0.18 | | 0.04 | | 0.46 | |  |
| *Medium HDLP (µmol/L)* | 0.08  (-0.42, 0.59) | 0.75 | | -0.25  (-0.73, 0.23) | | 0.30 | 0.68  (0.19, 1.16) | | 0.006 | 0.34 | | 0.10 | | 0.008 | |  |
| *Small HDLP (µmol/L)* | 1.34  (0.64, 2.04) | <.001 | | 0.73  (0.08, 1.39) | | 0.03 | 1.53  (0.86, 2.21) | | <.001 | 0.21 | | 0.70 | | 0.09 | |  |
| HDL Size (nm) | -0.02  (-0.08, 0.03) | 0.42 | | -0.00  (-0.06, 0.05) | | 0.87 | -0.04  (-0.09, 0.01) | | 0.12 | 0.64 | | 0.61 | | 0.31 | |  |
| HDL-cholesterol (mg/dL) | 4.57  (2.71, 6.42) | <.001 | | 1.65  (-0.08, 3.39) | | 0.06 | 3.47  (1.69, 5.25) | | <.001 | 0.02 | | 0.40 | | 0.15 | |  |
| **^b^**  Triglycerides | 1.26  (1.15, 1.38) | <.001 | | 1.11  (1.02, 1.21) | | 0.02 | 1.36  (1.24, 1.49) | | <.001 | 0.06 | | 0.25 | | 0.002 | |  |
| Total Cholesterol (mg/dL) | 1.20  (-4.22, 6.63) | 0.66 | | 0.51  (-4.60, 5.61) | | 0.84 | 6.18  (0.96, 11.41) | | 0.02 | 0.85 | | 0.19 | | 0.13 | |  |

**^a^** change based on estimated marginal means adjusted for the randomization stratification factors of site, race, and metabolic syndrome

**^b^** data were log-transformed for analysis and changes reported as ratio of geometric marginal means

^C^ comparison of the ORs between groups.

**UNADJUSTED Table S7B. Change in additional secondary outcomes from baseline to end of study within each group and comparison between OCP, metformin and combined groups**

|  | **COCP**  **N=79** | | | | **Metformin**  **N=81** | | | **Combined**  **n-80** | | | **^c^COCP**  **vs. Metformin** | | **^c^COCP**  **vs. Combined** | | **^c^Metformin vs. Combined** | |
| --- | --- | --- | --- | --- | --- | --- | --- | --- | --- | --- | --- | --- | --- | --- | --- | --- |
|  | **Change from Baseline**  **(95% CI)^a^** | | **P-value** | | **Change from Baseline**  **(95% CI) ^a^** | **P-value** | | **Change from Baseline**  **(95% CI) ^a^** | **P-value** | | **P-value** | | **P-value** | | **P-value** | |
| **PCOSQ and mental health surveys** | | | | | | | | | | | | | | | | |
| Emotion  domain | 0.39 (0.12, 0.67) | | 0.005 | | 0.40 (0.14, 0.66) | 0.003 | | 0.44 (0.17, 0.70) | 0.001 | | 0.97 | | 0.82 | | 0.84 | |
| Body Hair  domain | 0.74 (0.46, 1.03) | | <.001 | | 0.26 (-0.01, 0.53) | 0.06 | | 0.62 (0.35, 0.90) | <.001 | | 0.02 | | 0.55 | | 0.06 | |
| Weight domain | 0.71 (0.36, 1.06) | | <.001 | | 0.34 (0.00, 0.67) | 0.05 | | 0.85 (0.51, 1.19) | <.001 | | 0.13 | | 0.58 | | 0.04 | |
| Infertility  domain | 0.55 (0.22, 0.88) | | 0.001 | | 0.48 (0.16, 0.80) | 0.004 | | 0.48 (0.16, 0.80) | 0.004 | | 0.76 | | 0.78 | | 0.98 | |
| Menstrual  Problem  domain | 0.28 (-0.03, 0.59) | | 0.07 | | 0.45 (0.15, 0.75) | 0.003 | | 0.67 (0.37, 0.97) | <.001 | | 0.45 | | 0.08 | | 0.30 | |
| Acne  domain | 0.37 (0.07, 0.66) | | 0.01 | | 0.40 (0.11, 0.68) | 0.006 | | 0.78 (0.50, 1.07) | <.001 | | 0.88 | | 0.05 | | 0.06 | |
| CES-D Total Score | -1.92 (-4.45, 0.61) | | 0.14 | | -6.09 (-8.50, -3.69) | <.001 | | -0.45 (-2.89, 1.99) | 0.72 | | 0.02 | | 0.41 | | 0.001 | |
| **Lipoprotein Analysis** | | | | | | | | | | | | | | | | |
| Total TRLP (nmol/L) | -2.69 (-15.17, 9.79) | 0.67 | | 6.33 (-5.38, 18.03) | | 0.29 | 10.17 (-1.79, 22.13) | | 0.10 | 0.30 | | 0.14 | | 0.65 | |  |
| *Very Large TRLP*  *(nmol/L)* | -0.01 (-0.12, 0.10) | 0.83 | | -0.02 (-0.12, 0.09) | | 0.73 | 0.14 (0.04, 0.25) | | 0.009 | 0.94 | | 0.05 | | 0.04 | |  |
| *Large TRLP (nmol/L)* | 0.77 (-0.28, 1.83) | 0.15 | | 0.87 (-0.13, 1.86) | | 0.09 | 2.09 (1.08, 3.11) | | <.001 | 0.90 | | 0.08 | | 0.09 | |  |
| *Medium TRLP (nmol/L)* | 0.80 (-2.62, 4.23) | 0.64 | | 3.98 (0.76, 7.20) | | 0.02 | 4.58 (1.30, 7.86) | | 0.007 | 0.18 | | 0.12 | | 0.80 | |  |
| *Small TRLP (nmol/L)* | -18.19 (-27.04, -9.33) | <.001 | | -1.81 (-10.22, 6.59) | | 0.67 | -16.31 (-24.89, -7.73) | | <.001 | 0.009 | | 0.76 | | 0.02 | |  |
| *Very Small TRLP*  *(nmol/L)* | 15.26 (2.96, 27.56) | 0.02 | | 2.88 (-8.70, 14.45) | | 0.62 | 19.19 (7.38, 31.00) | | 0.002 | 0.15 | | 0.65 | | 0.05 | |  |
| **^b^**  TRL Triglycerides | 1.05 (0.92, 1.21) | 0.45 | | 1.16 (1.02, 1.32) | | 0.03 | 1.20 (1.05, 1.37) | | 0.009 | 0.34 | | 0.20 | | 0.72 | |  |
| **^b^**  TRL Cholesterol | 1.21 (1.11, 1.31) | <.001 | | 1.11 (1.02, 1.20) | | 0.01 | 1.30 (1.20, 1.41) | | <.001 | 0.14 | | 0.21 | | 0.005 | |  |
| Total LDLP (nmol/L) | 148.04 (88.14, 207.93) | <.001 | | -6.46 (-62.75, 49.83) | | 0.82 | 135.22 (77.65, 192.80) | | <.001 | <.001 | | 0.76 | | <.001 | |  |
| *Large LDLP (nmol/L)* | -67.11 (-110.8, -23.44) | 0.003 | | -57.52 (-98.84, -16.20) | | 0.007 | -41.69 (-83.91, 0.54) | | 0.05 | 0.75 | | 0.41 | | 0.60 | |  |
| *Medium LDLP (nmol/L)* | 67.44 (-10.34, 145.21) | 0.09 | | -6.15 (-79.98, 67.68) | | 0.87 | 59.69 (-15.62, 135.00) | | 0.12 | 0.18 | | 0.89 | | 0.22 | |  |
| *Small LDLP (nmol/L)* | 163.96 (80.58, 247.33) | <.001 | | 60.04 (-18.44, 138.52) | | 0.13 | 114.17 (34.00, 194.34) | | 0.005 | 0.08 | | 0.40 | | 0.34 | |  |
| LDL Size (nm) | -0.19 (-0.28, -0.10) | <.001 | | -0.11 (-0.19, -0.03) | | 0.006 | -0.14 (-0.22, -0.06) | | 0.001 | 0.19 | | 0.39 | | 0.65 | |  |
| LDL-cholesterol (mg/dL) | -6.73 (-11.24, -2.22) | 0.004 | | -3.22 (-7.47, 1.03) | | 0.14 | -4.04 (-8.38, 0.31) | | 0.07 | 0.27 | | 0.40 | | 0.79 | |  |
| Total HDLP (µmol/L) | 1.79 (1.15, 2.44) | <.001 | | 0.59 (-0.01, 1.20) | | 0.05 | 2.22 (1.61, 2.84) | | <.001 | 0.008 | | 0.34 | | <.001 | |  |
| *Large HDLP (µmol/L)* | 0.36 (0.13, 0.60) | 0.003 | | 0.14 (-0.08, 0.36) | | 0.21 | 0.02 (-0.21, 0.25) | | 0.85 | 0.18 | | 0.04 | | 0.46 | |  |
| *Medium HDLP (µmol/L)* | 0.09 (-0.42, 0.60) | 0.73 | | -0.26 (-0.74, 0.22) | | 0.28 | 0.67 (0.19, 1.16) | | 0.007 | 0.32 | | 0.10 | | 0.007 | |  |
| *Small HDLP (µmol/L)* | 1.34 (0.64, 2.04) | <.001 | | 0.74 (0.08, 1.40) | | 0.03 | 1.53 (0.86, 2.20) | | <.001 | 0.22 | | 0.70 | | 0.10 | |  |
| HDL Size (nm) | -0.02 (-0.07, 0.04) | 0.49 | | -0.00 (-0.06, 0.05) | | 0.86 | -0.04 (-0.09, 0.01) | | 0.12 | 0.70 | | 0.55 | | 0.32 | |  |
| HDL-cholesterol (mg/dL) | 4.61 (2.75, 6.46) | <.001 | | 1.64 (-0.10, 3.38) | | 0.06 | 3.46 (1.68, 5.24) | | <.001 | 0.02 | | 0.38 | | 0.15 | |  |
| **^b^**  Triglycerides | 1.25 (1.14, 1.37) | <.001 | | 1.11 (1.02, 1.21) | | 0.02 | 1.35 (1.24, 1.48) | | <.001 | 0.07 | | 0.23 | | 0.002 | |  |
| Total Cholesterol (mg/dL) | 1.15 (-4.30, 6.59) | 0.68 | | 0.50 (-4.63, 5.62) | | 0.85 | 6.10 (0.86, 11.34) | | 0.02 | 0.86 | | 0.20 | | 0.13 | |  |

**^a^** change based on estimated marginal means

**^b^** data were log-transformed for analysis and changes reported as ratio of geometric marginal means

**^c^** comparison of the ORs between groups.
