# Supplementary material for: Impact of combined hormonal contraceptives and metformin on metabolic syndrome in women with hyperandrogenic polycystic ovary syndrome and obesity: The COMET-PCOS randomized clinical trial
Source: PLoS Med. 2025 Dec 8;22(12):e1004662. doi: 10.1371/journal.pmed.1004662 (PMC12697981; doi:10.1371/journal.pmed.1004662)
Supplement: S8 Table — (DOCX) [file pmed.1004662.s013.docx]

**S8 Table. Serious Adverse Events**

|  | **Arm** | **Brief Description of Event** | **Reason it was serious** | **Resolution** | **Study Status** |
| --- | --- | --- | --- | --- | --- |
| **1** | COCP | Gastroenteritis resulting in dehydration. | Hospitalization required | Resolved/  recovered | terminated |
| **2** | COCP | Exploratory laparoscopy for severe lower abdominal pain | Surgical intervention | Resolved/  recovered | terminated |
| **3** | Metformin | Participant hospitalized for severe depression. | Hospitalization required | Improving after discharge | terminated |
| **4** | Metformin | Pregnancy during study, miscarriage at 20 weeks 5 days gestation. | Hospitalization required | Resolved/  recovered | terminated |
| **5** | Metformin | Ongoing bleeding despite medical management. Admitted for blood transfusion and D&C | Hospitalization required | Resolved/  recovered | Completed study |
| **6** | Combined | Gall bladder attack and had surgery. | Hospitalization required | Resolved/  recovered | terminated |
| **7** | Combined | Headache/vision problems, went to ER, diagnosed with idiopathic intracranial hypertension | Medical intervention | Resolved/  recovered | terminated |
| **8** | Combined | Gastrointestinal symptoms and had cholecystectomy | Hospitalization required | Resolved/  recovered | Completed study |
