# Supplementary material for: Impact of combined hormonal contraceptives and metformin on metabolic syndrome in women with hyperandrogenic polycystic ovary syndrome and obesity: The COMET-PCOS randomized clinical trial
Source: PLoS Med. 2025 Dec 8;22(12):e1004662. doi: 10.1371/journal.pmed.1004662 (PMC12697981; doi:10.1371/journal.pmed.1004662)
Supplement: S9 Table — (DOCX) [file pmed.1004662.s014.docx]

**S9 Table. Change in MetS Prevalence Based on Presence or Absence of MetS at Baseline**

**Group – Metformin**

| **MetS**  **baseline** | **MetS**  **end of study** | **Frequency** | **Percent** | **Cumulative Frequency** | **Cumulative Percent** |
| --- | --- | --- | --- | --- | --- |
| **no** | **no** | 39 | 60.00 | 39 | 60.00 |
| **no** | **yes** | 6 | 9.23 | 45 | 69.23 |
| **yes** | **no** | 9 | 13.85 | 54 | 83.08 |
| **yes** | **yes** | 11 | 16.92 | 65 | 100.00 |

**Group - OCP + Metformin**

| **MetS**  **baseline** | **MetS**  **end of study** | **Frequency** | **Percent** | **Cumulative Frequency** | **Cumulative Percent** |
| --- | --- | --- | --- | --- | --- |
| **no** | **no** | 36 | 57.14 | 36 | 57.14 |
| **no** | **yes** | 6 | 9.52 | 42 | 66.67 |
| **yes** | **no** | 9 | 14.29 | 51 | 80.95 |
| **yes** | **yes** | 12 | 19.05 | 63 | 100.00 |

**Group - OCP**

| **MetS**  **baseline** | **MetS**  **end of study** | **Frequency** | **Percent** | **Cumulative Frequency** | **Cumulative Percent** |
| --- | --- | --- | --- | --- | --- |
| **no** | **no** | 39 | 66.10 | 39 | 66.10 |
| **no** | **yes** | 5 | 8.47 | 44 | 74.58 |
| **yes** | **no** | 3 | 5.08 | 47 | 79.66 |
| **yes** | **yes** | 12 | 20.34 | 59 | 100.00 |
